# A Single Cohesin Complex Performs Mitotic and Meiotic Functions in the Protist *Tetrahymena*

**DOI:** 10.1371/journal.pgen.1003418

**Published:** 2013-03-28

**Authors:** Rachel A. Howard-Till, Agnieszka Lukaszewicz, Maria Novatchkova, Josef Loidl

**Affiliations:** 1 Department of Chromosome Biology and Max F. Perutz Laboratories, Center for Molecular Biology, University of Vienna, Vienna, Austria; 2 Research Institute of Molecular Pathology (IMP), Vienna, Austria; 3 IMBA, Institute of Molecular Biotechnology of the Austrian Academy of Sciences, Vienna, Austria; Imperial College London, United Kingdom

## Abstract

The cohesion of sister chromatids in the interval between chromosome replication and anaphase is important for preventing the precocious separation, and hence nondisjunction, of chromatids. Cohesion is accomplished by a ring-shaped protein complex, cohesin; and its release at anaphase occurs when separase cleaves the complex's α-kleisin subunit. Cohesin has additional roles in facilitating DNA damage repair from the sister chromatid and in regulating gene expression. We tested the universality of the present model of cohesion by studying cohesin in the evolutionarily distant protist *Tetrahymena thermophila*. Localization of tagged cohesin components Smc1p and Rec8p (the α-kleisin) showed that cohesin is abundant in mitotic and meiotic nuclei. RNAi knockdown experiments demonstrated that cohesin is crucial for normal chromosome segregation and meiotic DSB repair. Unexpectedly, cohesin does not detach from chromosome arms in anaphase, yet chromosome segregation depends on the activity of separase (Esp1p). When Esp1p is depleted by RNAi, chromosomes become polytenic as they undergo multiple rounds of replication, but fail to separate. The cohesion of such bundles of numerous chromatids suggests that chromatids may be connected by factors in addition to topological linkage by cohesin rings. Although cohesin is not detected in transcriptionally active somatic nuclei, its loss causes a slight defect in their amitotic division. Notably, *Tetrahymena* uses a single version of α-kleisin for both mitosis and meiosis. Therefore, we propose that the differentiation of mitotic and meiotic cohesins found in most other model systems is not due to the need of a specialized meiotic cohesin, but due to additional roles of mitotic cohesin.

## Author Summary

During cell division, identical DNA molecules, packaged in the sister chromatids of a chromosome, must be distributed to daughter cells. The cohesion of sister chromatids in the interval between DNA replication and mitotic anaphase is important for preventing the precocious separation, and hence nondisjunction, of chromatids. Cohesion is accomplished by a ring-shaped protein complex, cohesin; and a popular model of cohesion holds that sister chromatids are encircled by cohesin rings and separate upon opening of the rings. During meiosis, cohesin, together with chiasmata, has the additional function of holding bivalents together. Cohesin also has functions in gene regulation and DNA damage repair, and has recently garnered attention as a factor involved in human congenital birth defects. We have studied cohesin in the protist *Tetrahymena*, which has mitosis/meiosis and transcription performed by different nuclei within the same cell. We exploited this unique feature to experimentally separate the functions of cohesin in chromosome segregation and gene regulation. While the cohesin machinery is generally conserved between eukaryotes, *Tetrahymena*'s phylogenetic distance from standard model organisms allowed us to discover some notable adaptations during the course of evolution.

## Introduction

Cohesin is a ring-shaped protein complex which holds sister chromatids together to prevent their untimely separation prior to anaphase (see [1]). It consists of four core components, Smc1 and Smc3, Scc3, and a member of the conserved α-kleisin family of proteins, Mdc1/Rad21/Scc1 in mitotic cells, or Rec8 in meiotic cells [1], [2].

In mitotic cells, newly synthesized sister chromatids are linked by cohesin. In some organisms, chromatids separate along their arms during prophase and at the centromeres during anaphase, while in other organisms separation occurs in a single step during anaphase. The resolution of cohesion during anaphase is initiated by the cleavage of the α-kleisin component of the cohesin, the opening of the ring structure, and the disappearance of the cohesin from the chromosomes. This allows the mitotic separation of the sister chromatids. The fact that ring opening releases the chromatids, together with other evidence (see [1]), led to the popular model that cohesin works by the enclosure of the two sisters by a single ring.

During meiosis, homologous chromosomes first pair and become connected by a protein structure, the synaptonemal complex (SC) (see [3]). At the same time, deliberate DNA double-strand breaks (DSBs) are formed and resolved in a way that leads to recombination [4]. Subsequent segregation of homologous chromosomes and sister chromatids is coordinated by the sequential loss of a specialized meiotic cohesin from the arms prior to the first meiotic division, and then from centromere regions prior to the second division. First, release of cohesin from regions distal to chiasmata allows the separation of homologous chromosomes, then release of centromeric or proximal regions separates sisters. Meiosis-specific regulation of cohesion works only when a specialized meiotic cohesin (containing the meiosis specific α-kleisin Rec8, and in some organisms also meiotic versions of other cohesin components) is loaded onto chromosomes during the premeiotic S-phase (for review see [5]). Kleisin cleavage in mitosis and meiosis is performed by a protein called separase, whose checkpoint-dependent activation relies upon the orderly association of chromosomes or bivalents with the division spindle, thus assuring their faithful disjunction (see [1], [6]). During meiotic prophase, cohesin is associated with the axial elements of SCs [7]–[11] and is involved in the repair and, in some organisms, the formation of meiotic DNA DSBs [12].

In addition to its well-established function in controlling chromatid and chromosome segregation, cohesin was found to play some additional roles, such as in DNA damage repair, where it promotes recombinational repair via sister DNA molecules [13], [14], in maintaining epigenetic inheritance states [15], in gene regulation [16] (and lit. cit. therein) and in chromosome conformation [17].

**Figure 1:**
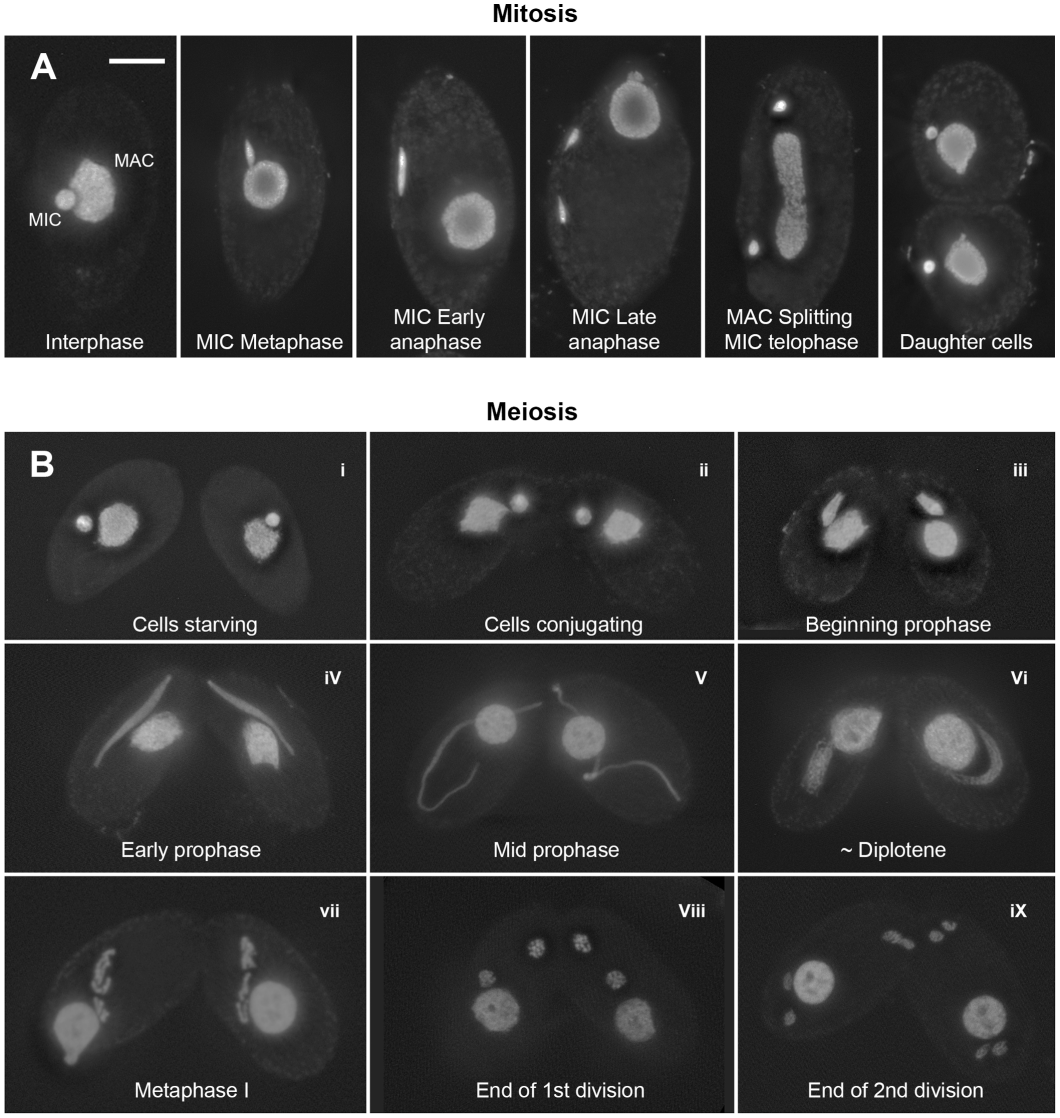
Mitotic and meiotic stages. (A) Mitosis. In nondividing vegetative cells, the diploid micronucleus (MIC) rests in a pocket in the polyploid macronucleus (MAC). During mitosis, it becomes stretched and divides. The MAC splits (by an amitotic process) only after separate daughter MICs have formed. (B) Meiosis. Cells of complementing mating types fuse (“conjugate” – i–ii) and their diploid MICs perform synchronous meioses. During prophase (i–vi), the MIC elongates to >2× the cell length (iii–v), before it shortens again (vi). In contracting MICs, chromatin condensation culminates in the formation of condensed metaphase bivalents (vii). Subsequently, homologs separate in a first (viii) and sisters in a second meiotic division (ix). DNA stained by DAPI. Bar: 10 µm.

*Tetrahymena thermophila* is a ciliated protist. Like the other ciliates, *Tetrahymena* possesses its soma and its germline organized as two nuclei within one cell (see [18]). The germline micronucleus (MIC) carries a diploid number of 10 chromosomes that undergo closed mitosis and meiosis (Figure 1). The MIC genome is not expressed, and so the MIC is largely dispensable for vegetative proliferation of the cell; its only function is the propagation of heritable information during sexual reproduction. Transcription takes place only in the somatic macronucleus (MAC). The MAC contains ∼45-fold amplified copies of the genome which are distributed in ∼180 minichromosomes that lack centromeres and remain uncondensed. It does not divide by a mitotic process, but splits into roughly equal parts prior to cell division.

*Tetrahymena* cells of complementing mating types join (“conjugate”) under starvation conditions and initiate synchronous meioses in their MICs (Figure 1). The MAC does not undergo meiosis but it degenerates, and a new MAC regenerates from the MIC in new sexual progeny. Meiosis is characterized by several unusual features: First, there is no dedicated premeiotic S-phase; cells at (micronuclear) G_2_ conjugate and enter the meiotic program. Moreover, *Tetrahymena* lacks an SC [19] and, most notably, the MIC undergoes an immense elongation, to up to 50 times its original diameter, during meiotic prophase [20]–[22]. Within the elongated MIC, chromosomes are arranged in parallel, which facilitates homologous pairing and recombination [23].

Here, we take advantage of *Tetrahymena*'s evolutionary distance from other commonly studied model systems to learn more about what features of cohesin are conserved, and what have been adapted to the needs of the particular organism. We also exploit the nuclear duality of *Tetrahymena* to separate functions of cohesin proteins that are important for chromosome division from functions related to gene expression and regulation. We demonstrate that *Tetrahymena* has evolved notable differences to the standard eukaryotic cohesion machinery. Investigation of these remarkable adaptations will lead to new insights into the flexibility of the chromosome cohesion and division processes.

## Results

### *Tetrahymena* possesses conserved components of the cohesion machinery, but only a single kleisin homolog

**Figure 2:**
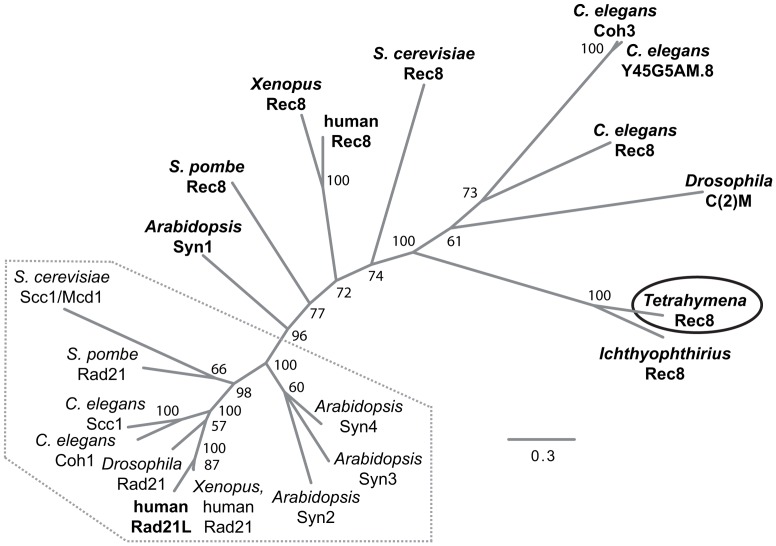
Phylogenetic relationships among eukaryotic α-kleisin family members. The unrooted phylogenetic tree was generated using MrBayes 3.2 [74] and visualized using FigTree. It shows experimentally confirmed meiotic (bold) and mitotic (regular) cohesins. Mitotic α-kleisins form a subgroup (dotted area) with closer sequence similarity, as indicated by the shorter branch lengths. Meiotic α-kleisins and *Tetrahymena* Rec8p show higher sequence diversity. The function of *Drosophila* C(2)M as the meiotic kleisin is still debated [62]. % Bayesian posterior probabilities are indicated. Bar: Number of amino acid substitutions per site.

We performed a bioinformatic search of the *T. thermophila* proteome [24] for the presence of cohesin components (see Text S1). The protein encoded by TTHERM_00245660 was the top and significant hit identified in searches for homologs to the Scc1(Mcd1)/Rad21/Rec8 family of α-kleisin proteins [2]. It produced a significant match in region aa 579–607 of the 619 amino acid-protein to the conserved C-terminal winged helix domain [25]. An additional weaker similarity was found for the N-terminal aa 30–130 (Figure S1). Based on the alignment of the conserved N- and C-terminal regions, we generated a phylogenetic tree that shows a relatively close sequence relationship of the group of mitotic α-kleisins, whereas meiotic members and TTHERM_00245660p show higher sequence divergence (Figure 2).

Another protein, TTHERM_00219160p, is characterized by a C-terminal winged helix domain with more distant similarity to kleisins. It was found as the second best and significant hit in profile searches with the winged helix domain that was used to identify TTHERM_00245660p. This similarity was also confirmed in reciprocal searches (see Text S1, Figure S1). However, sequence similarity beyond the C-terminal region could not be detected. TTHERM_00219160 mRNA has very low expression compared to the other cohesin gene homologs, and does not show significant up-regulation during conjugation, as the others do [26]. GFP tagging showed no detectable expression or localization of TTHERM_00219160p, even at high levels of replacement of the wildtype gene with the tagged gene (data not shown). All of this evidence suggests that this is not a true kleisin protein with cohesin function, leaving TTHERM_00245660p as the only likely candidate.

It is notable that *Tetrahymena* would have only a single α-kleisin homolog, because all eukaryotes studied to date in this respect have mitotic (Scc1/Rad21/Mcd1) and meiotic (Rec8) versions of this protein. Despite the lack of any closer relationship to the meiotic subgroup of α-kleisins (Figure 2), we will designate TTHERM_00245660p as Rec8p (and the gene as *REC8*) in the following, because of its function in mitosis and meiosis (see below). It shares this feature with the meiotic budding yeast Rec8 which, in principle, can take over the mitotic cohesion function [27]–[29].

Other core components of a putative cohesin complex show more conservation than Rec8. ORFs TTHERM_01048090 and TTHERM_00294810 encode clear Smc1 and Smc3 homologs, respectively [24], [30]. It is likely that the *SMC3* ORF prediction is incorrect [24], because cDNA coverage for *SMC3* in the *Tetrahymena* Functional Genomics Database (http://tfgd.ihb.ac.cn) suggests a protein of only 1187 amino acids. To confirm that putative cohesin proteins form a complex in *Tetrahymena*, an immunoprecipitation (IP) was performed using cells expressing Rec8-GFP. Wild type cells were used for a control IP. Mass spectrometry analysis of the precipitating proteins showed that Smc1 and Smc3 were enriched 13× and 9×, respectively, in the Rec8-GFP pulldown sample over the untagged Rec8 control sample. Of 1837 identified proteins, Smc1 and Smc3 ranked 1^st^ and 4^th^ with respect to the number of unique peptides identified in the pulldown sample but only at positions 521 and 703 in the control (Table S1). Sequence coverage was 56% vs. 3% for Smc1 and 47% vs. 8% for Smc3 in the pulldown and control samples (Figure S2). This is strong evidence for the involvement of Rec8, Smc1 and Smc3 in a protein complex and hence a confirmation of their correct identification as cohesin proteins.

An Scc3/Rec11 homolog candidate, encoded by TTHERM_00225630, was identified in profile searches versus the *T. thermophila* proteome with the region of best conservation from known Scc3/Rec11 orthologs, and was also confirmed in reciprocal searches (see Text S1). Although we did not find this homolog precipitating with Rec8-GFP, we will designate it as Scc3p. In addition to the core subunits of the cohesin complex, we were also able to identify the separase protein, another component of the cohesion/segregation machinery. TTHERM_00297160 is annotated in the *Tetrahymena* Genome database as the separase gene *ESP1* [24]. The Esp1 protein sequence produced best reciprocal BLASTp hits with *Saccharomyces cerevisiae* Esp1, *Schizosaccharomyces pombe* Cut1, *Caenorhabditis elegans* SEP-1, *Arabidopsis thaliana* AESP and human ESPL1. We did not yet find obvious candidates for accessory cohesion proteins such as Eco1, Pds5, Wapl, sororin or securin.

### Rec8p and Smc1p localize to both mitotic and meiotic nuclei and remain associated with chromosome arms during divisions

**Figure 3:**
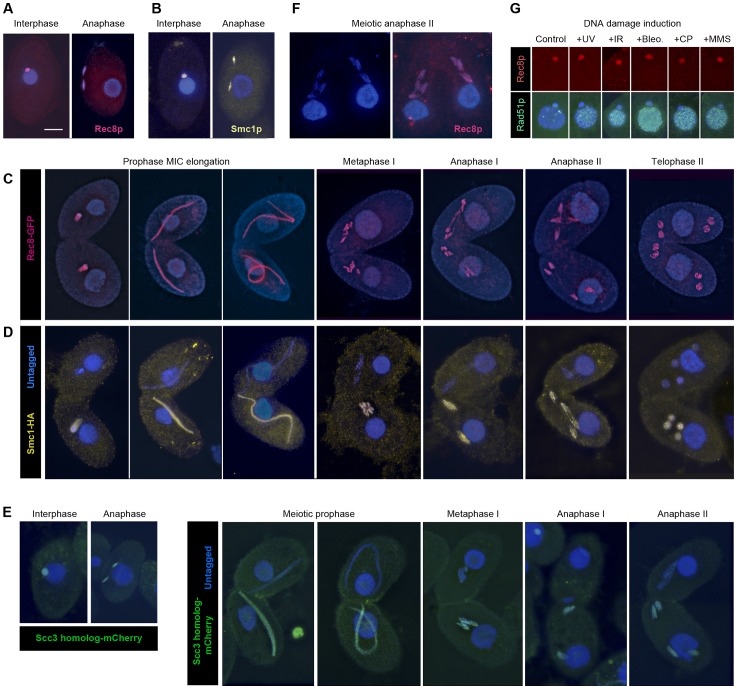
Localization of Rec8p, Smc1p, and Scc3p to mitotic and meiotic chromosomes. (A, B) Rec8-GFP (red) and Smc1-HA (yellow) localize to interphase and mitotic MICs. (C) Rec8p localizes to MIC chromosomes during all stages of meiosis (both mating partners express GFP-tagged Rec8). (D) Smc1p localizes to MIC chromosomes during all stages of meiosis (only one partner expresses HA-tagged Smc1). Note the absence of both cohesion proteins from the MACs. (E) The Scc3p homolog TTHERM_00225630p (green) localizes to mitotic and meiotic MICs of all stages. (F) Paired cells in meiotic anaphase II prepared by a spreading method to remove free nuclear proteins. Persistance of Rec8 staining (red) demonstrates that it remains attached to the arms of separating chromatids. (G) MAC localization of Rec8p is not increased, whereas repair protein Rad51 is strongly expressed in the MAC upon DNA damage. Bar: 10 µm in A–F.

In order to observe the localization of cohesin in *Tetrahymena*, we used strains expressing tagged fusion proteins Rec8-GFP and Smc1-HA. Immunostaining against GFP or HA revealed a micronuclear localization of both proteins, in both vegetatively growing and sexually reproducing cells. This localization is maintained throughout all stages of mitosis and meiosis (Figure 3A–3D, Figure S3A, Figure S4). In addition, we tagged the putative Scc3p with mCherry and found its localization to be the same (Figure 3E). In elongated meiotic nuclei, which correspond to the prophase stage of meiosis, Rec8p, Smc1p and Scc3p can be seen along the entire length of the chromosomes (Figure 3C, 3D, 3E, Figure S4). When only one of two conjugating meiotic cells carried the tagged proteins (as shown for Smc1-HA and Scc3-mCherry in Figure 3D, 3E and Figure S4), the protein was found retained in this cell. This is in contrast to the previously observed exchange of proteins between conjugating partners [31] and indicates a low turnover of cohesin proteins.

Notably, in mitotic anaphase as well as in anaphase I and II of meiosis, the proteins could be seen along the full length of the stretched chromosome arms (Figure 3A–3E). This is in contrast to the expected behavior shown by other organisms [7], [32]–[36], where cohesin disappears from chromosome arms during anaphase, but is similar to fission yeast mitosis [37], where the separation of sister chromatids is not accompanied by the removal of cohesin. It is possible that the anaphase signal could result from cleaved cohesin rings that remain in the nucleoplasm or loosely associated with chromosomes during closed divisions. In order to address this possibility, detergent-spread fixations were performed in which free proteins are washed out of the MIC [38]. Immunofluorescent stainings of these fixations still showed the continuous localization of Smc1-HA and Rec8-GFP on chromosome arms throughout anaphase (Figure 3F). This indicates that cohesin remains attached to chromatin during divisions.

It was shown by Feulgen microspectrophotometry that in the *Tetrahymena* vegetative cell cycle, MIC DNA replication starts during division, as early as late anaphase or telophase [39]. We confirmed this staging by BrdU incorporation (Figure S3B). This early replication may provide an explanation for the presence of cohesin along mitotic chromatids. Cohesin may be present in a non-cohesive form in order to establish cohesion as soon as replication starts.

### Cohesin is not detected in the amitotic macronucleus

*Tetrahymena* allows separate observation of different cohesin functions because mitosis and transcription are carried out in separate nuclei within one and the same cell. We did not observe Rec8p nor Smc1p in the MAC of vegetatively growing or meiotic cells (Figure 3A–3D, 3F). In principle, there should be no strict need for cohesin in the MAC, because the polyploid macronuclear chromosomes separate randomly by an amitotic process, and therefore it is not necessary to hold sister chromatids together after replication. However, an increasing number of non-cohesive functions of cohesin have been reported in studies of other model organisms (for reviews see [1], [6], [40]). If cohesin performs these functions in *Tetrahymena*, it could conceivably be needed in the MAC as well as the MIC. Because cohesin has previously been shown to localize to sites of broken DNA [41], [42], we hypothesized that macronuclear cohesin might be induced by DNA damage. It has been shown in *Tetrahymena* that DNA damage induces expression of Rad51p in the MAC [43]. This suggests that a recombinational repair pathway is triggered that might rely on the physical linkage of sister DNA molecules by cohesin. Cells expressing Rec8-GFP were treated with UV-C, γ-radiation, MMS, cisplatin, or bleomycin (see Materials and Methods), all of which are known to induce DNA damage in *Tetrahymena* [23], [44]. Cells were fixed 0.5 or 1.5 h after treatment. Immunostaining against GFP showed no macronuclear localization while the DNA repair protein Rad51 was abundant in the MAC in response to the DNA damage (Figure 3G). 200 cells with Rad51-positive MACs were evaluated per experiment. In all cases, Rec8p was detected in the MIC but never in the MAC. A similar experiment did not detect damage-induced Smc1-HA in the MAC, either (data not shown). These results seem to indicate that cohesin does not localize to the MAC. Therefore, if cohesin functions in the MAC, it must be at concentrations too low to observe cytologically.

### Loss of cohesin impairs vegetative propagation and causes MIC chromosome mis-segregation

To learn more about the function of cohesin in *Tetrahymena*, depletion of Rec8p or Smc1p was performed by RNA interference (RNAi). For this, cells were transformed with constructs to express hairpin (hp) RNA molecules from a cadmium-inducible promoter, and RNAi was elicited in *SMC1*hp and *REC8*hp cells by growth in medium with CdCl_2_.

**Figure 4:**
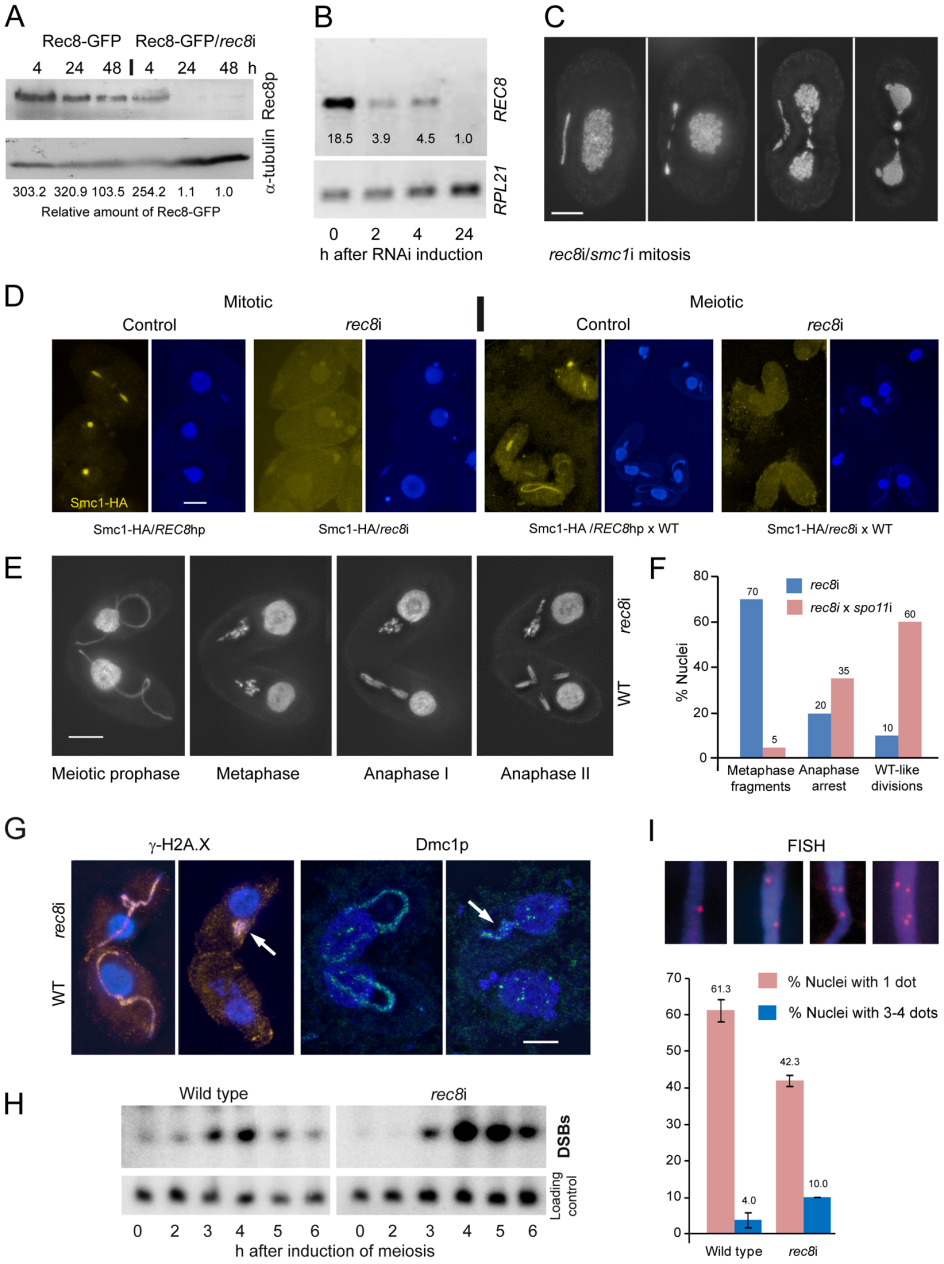
Phenotypes of cohesin-depleted cells. (A) Rec8p is depleted by cadmium (Cd)-induced expression of hairpin RNA and monitored by the detection of GFP-tagged Rec8p on a Western gel. RNAi for 24 h leads to virtually complete loss of Rec8p. GFP band intensities were determined using the “Analyze” tool of ImageJ (Wayne Rasband, N.I.H.; http://rsb.info.nih.gov/ij/), normalized to the α-tubulin loading control, and used to calculate relative protein amounts. (B) RT-PCR shows that *REC8* RNA is greatly reduced after 2 h of RNAi induction, and nearly absent after 24 h. (C) Mitotic division of the MIC shows delayed segregation and lagging chromosomes, and the MAC also splits abnormally in cells depleted of Rec8p and Smc1p. Bar: 10 µm. (D) Depletion of Rec8p causes the loss of Smc1p in mitotic and meiotic MICs. In the presence of Rec8p, Smc1-HA localizes to MICs in vegetatively propagating cells, but disappears when *rec8* RNAi is induced. When REC8hp/Smc1-HA cells are mated to a wild type (WT) partner, the former show micronuclear Smc1-HA localization, which disappears upon *rec8* RNAi. Bar: 10 µm. (E) *rec8*i cells (top) arrest at an abnormal metaphase-anaphase I stage, whereas the WT mating partners (bottom) show the normal progression of meiosis. In WT metaphase, 5 distinct bivalents can be seen (arrow points to one well-separated bivalent), whereas the *rec8*i partner shows fragmented chromosomes. Bar: 10 µm. (F) Quantitation of *rec8*i meiosis arrest 4,5 h after meiosis induction. Eliminating DSBs by mating *rec8*i cells with *spo11*i partners rescues the *rec8*i phenotype. Stages of *rec8*i cells were scored in cell pairs where their respective WT or *spo11*i mating partners had progressed beyond the stage indicated (n = 100 cells in both experiments). (G) DSB markers g-H2AX (orange) and Dmc1p (green) highlight elongated meiotic prophase MICs (left) in both *rec8*i and WT cells of a mating pair. Later in meiosis (right), when they have disappeared from the WT partner, they are still present in the arrested MICs of the *rec8*i partner (arrows). (H) Detection of DSBs by PFGE in WT and *rec8*i meiosis. The lower panel shows a control hybridization to the same membrane as a test for equal DNA loading and Southern transfer. (I) Evaluation of pairing (scored as the presence of a single FISH signal) and loss of cohesion (scored as the presence of 3 or 4 signals) in elongated meiotic prophase MICs, displayed by different genotypes. Examples of MICs with different numbers of FISH signals are shown on top. Values are means of three repeats with 50 nuclei evaluated, each. Error bars indicate standard deviation.

Induction of *rec8*RNAi (*rec8*i) in growing cells resulted in a very slow depletion of Rec8p, as can be seen by Western blotting in cells expressing both the Rec8-GFP and the *REC8*hp construct (Figure 4A). After 4 h of RNAi induction (approximately 1–2 cell divisions), large amounts of Rec8p remained in the cells, whereas *REC8* mRNA was substantially reduced even after 2 h, as seen by RT-PCR (Figure 4B). Only after 24 hours of RNAi, was the protein almost completely depleted (Figure 4A). This data also confirms that Rec8p has a very low turnover in the cell (see above), in contrast to RNAi targets studied previously, which needed only short periods of RNAi induction to produce phenotypes [38], [45].

Depletion of Rec8p and/or Smc1p impaired the growth of cells. Viability was tested by isolating single cells of wild type (WT), *REC8*hp, *SMC1*hp or *REC8*hp/*SMC1*hp strains (n = 47 each) in drops of medium containing 0.1 µg/ml CdCl_2_ to induce RNAi. After 48 h of growth, 100% of WT clones had more than 100 cells, whereas 0% of *rec8*i, 4.2% of *smc1*i and 6.4% of *rec8*i/*smc1*i clones had grown to the same density.

Cells depleted of cohesins for at least 24 hours often showed lagging chromosomes in mitotic anaphase and, unlike in wild-type controls (Figure 1A), the MACs began to elongate before mitosis was complete (Figure 4C). This suggests that a lack of cohesion causes abnormal spindle attachments that result in a delay in chromatid segregation. *rec8*i/*smc1*i cells displayed 45% (n = 100) abnormal anaphases whereas the phenotype was less prevalent in the *smc1*i line (13% (n = 100) of anaphases were defective) and absent in the *rec8*i line. Thus, the additive effect of the double RNAi is necessary to strongly affect segregation. Notably, telophase MICs appeared normal, presumably because lagging or misoriented chromatids eventually manage to migrate to the poles. MACs also had problems in splitting (Figure 4C, Figure S5A), presumably as a consequence of delayed MIC mitosis. Cells often contained large amounts of DNA left between newly divided MACs, which is a less prevalent phenomenon in the wild type [46].

We next tested if depletion of Rec8p influences Smc1p localization. We constructed a strain that carries both the *REC8*hp and Smc1-HA. This strain showed the normal micronuclear localization of HA-tagged Smc1p (in 200 of 200 evaluted nuclei) when *REC8* RNAi was not induced (Figure 4D). However, when RNAi was induced (*rec8*i), Smc1p staining was lost (Figure 4D). In quantitative terms, in 96% of nuclei, the Smc1 signal was completely lost, and in 4% it was strongly reduced (n = 200 nuclei). When this strain was mated to wild type, Smc1-HA localized to meiotic MICs (in 200 of 200 evaluted nuclei), but mostly failed to do so upon RNAi depletion of Rec8 (Figure 4D). The Smc1 signal was reduced or completely lost, in 8% and 85% of nuclei, respectively (n = 200 nuclei). The dependency of Smc1 localization on Rec8 confirms a strong interdependence of Rec8 and Smc1 protein expression or stability, as would be expected for members of a protein complex such as cohesin. Moreover, it indicates that Rec8p is the predominant, if not only, kleisin partner of Smc1p, otherwise more Smc1p, which was forming a complex with another kleisin, would persist.

### Meiotic divisions are arrested upon the depletion of cohesin components

To evaluate the meiotic phenotype of *rec8*i and *smc1*i cells, RNAi was performed on growing cells for 24 hours to deplete cohesin prior to inducing meiosis. The cohesin-depleted cells were then mated with a WT strain expressing Rec8-GFP. Due to the low turnover rate of the cohesion proteins (see above), RNAi affected only the hairpin-carrying partner of mating pairs and allowed direct comparison of defective stages with the corresponding meiotic stages in the WT partner. DAPI staining of these mating cells showed that *rec8*i and *smc1*i cells begin meiosis normally and elongate the MIC, as in WT. However, whereas in WT cells, the MIC contracts and chromosomes condense to form bivalents in metaphase, in *rec8*i or *smc1*i cells, the chromosomes never fully condense (Figure 4E, Figure S5). In these cells, meiosis arrests at an abnormal metaphase-anaphase-like state, with clumps of fragmented and stretched chromatin, instead of distinct chromosomes, while the WT partner progresses normally through the meiotic divisions (Figure 4E). Quantitation of the *rec8*i meiotic arrest showed that in pairs where the WT partner had progressed to metaphase or beyond, 70% of the *rec8*i partners were arrested in the fragmented metaphase state, 20% attempted to undergo an abnormal anaphase, and 10% showed a normal looking division (Figure 4F). To determine if the *rec8*i arrest phenotype was dependent on meiotic DSBs, we depleted the Spo11 DSB nuclease by RNAi. *spo11* RNAi of one cell inhibits or reduces DSB formation in the partner cell as well (data not shown). When we mated *rec8*i cells with *spo11*i partners, the *rec8*i arrest phenotype was partially rescued. 60% of cells completed anaphase I, 35% showed abnormal divisions, and only 5% arrested with fragmented metaphase chromosomes (Figure 4F). This suggests that *rec8*i cells have a defect in DSB repair.

### Depletion of cohesin impairs DNA repair in MICs

To further test if meiotic DSB repair was affected, matings of *rec8*i and WT cells were stained with antibodies against γ-H2A.X or the recombination protein Dmc1. These DSB markers are normally seen primarily in the elongated pachytene MIC, and they disappear in later stages after DSBs are repaired [38], as can be seen in the WT partner (Figure 4G). However, γ-H2A.X and Dmc1 staining can still be seen in the *rec8*i cells, after their WT partners have completed DSB repair. This suggests that DSBs are not completely repaired in the absence of cohesin. The persistence of DSBs was also confirmed by electrophoretic detection of DSB-dependent chromosome fragments in mating *rec8*i cells (Figure 4H). Whole cell DNA preparations of mating cells were separated by pulsed field gel electrophoresis, blotted, and probed with a repetitive sequence found only in the MIC [44]. Under the conditions used, unbroken MIC chromosomes do not enter the gel, but chromosomes fragmented by DSBs migrate as a mass, creating a distinct band. In WT cells, DSBs appeared at 3 hours after induction of meiosis, and disappeared at 5–6 h as breaks were repaired and meiosis was completed (Figure 4H). In *rec8*i matings, however, the band representing meiotic DSBs appeared normally at 3 h, but seemed to accumulate to a higher level at 4 and 5 h, and did not disappear by 6 h after induction of meiosis. Therefore, we can conclude that cohesin is required for the repair of meiotic DSBs.

### Meiotic pairing and sister chromatid cohesion are reduced in cells depleted of Rec8p

Because defective DSB repair causes reduced meiotic pairing [38], we wondered if pairing was affected by the absence of Rec8. In meiosis of *Tetrahymena*, close pairing of homologous chromosomes is established in elongated MICs. Fluorescence in situ hybridization (FISH) can be used to monitor meiotic pairing of a chromosomal locus, which is indicated by the presence of a single FISH signal instead of two (Figure 4I). While a single signal could also result from mitotic nondisjunction (see above), *rec8*i did not show a mitotic phenotype. Therefore we were able to use *rec8*i cells to score pairing, using a probe to an intercalary chromosomal locus. In WT cells, 34.7% of meiotic prophase nuclei showed two FISH signals, corresponding to the unpaired homologous loci, and 61.3% showed only one signal, indicating pairing of that locus had occurred. In *rec8*i cells, pairing was reduced to 42.3%. (Figure 4I). At the same time, FISH was also used to evaluate cohesion. 10.0% of *rec8*i elongated nuclei showed 3 or 4 signals, as compared to 4.0% in WT (Figure 4I). Altogether, the depletion of cohesin results in a reduction of meiotic sister chromatid cohesion and homologous pairing.

### Separase (Esp1p) knockdown prevents chromosome segregation and causes polyploidization

**Figure 5:**
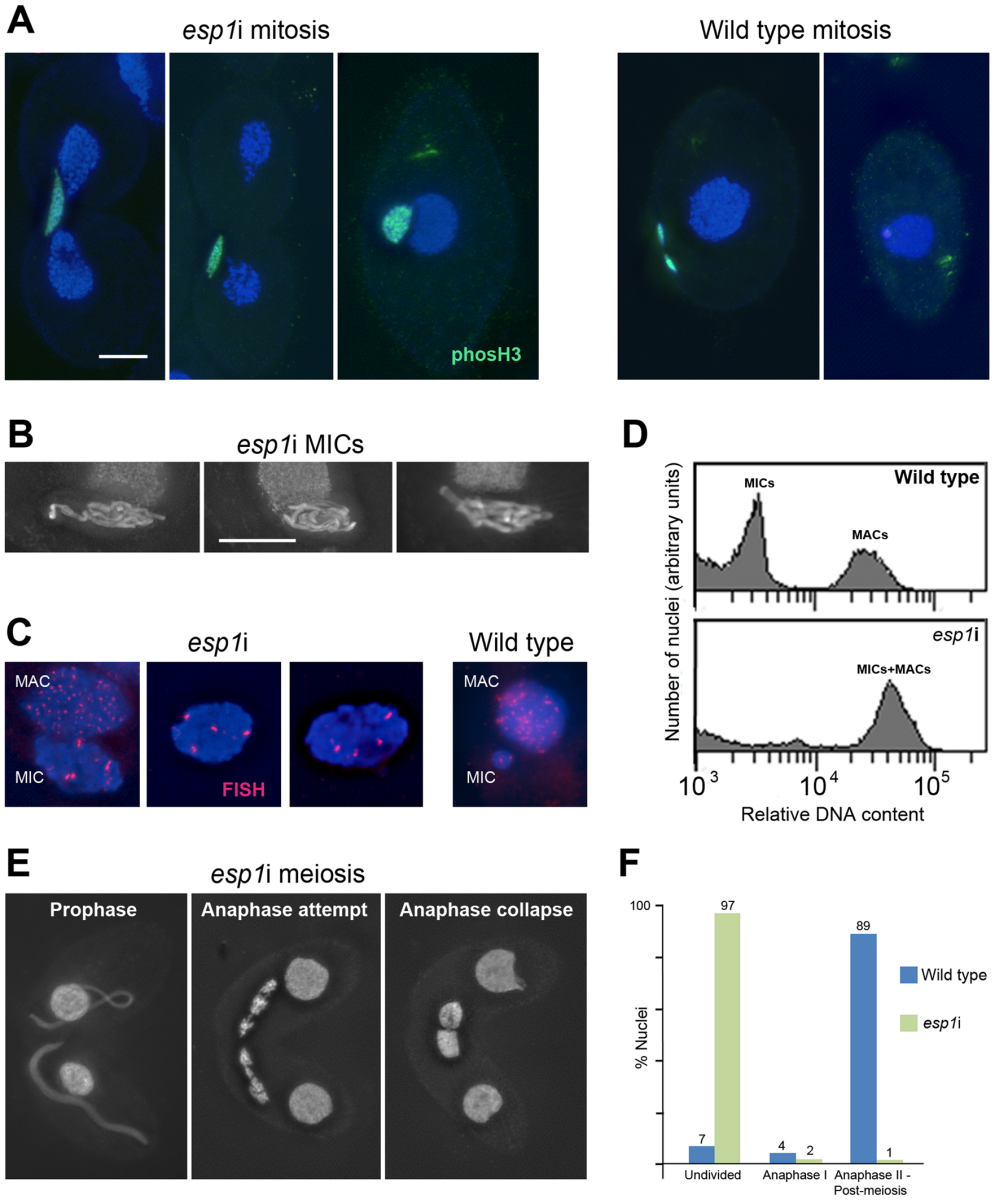
RNAi depletion of separase Esp1p prevents mitotic and meiotic division and causes MIC polyploidization. (A) Chromosomes fail to segregate during mitosis. Whereas in the WT MIC, division precedes the splitting of the MAC, the *esp1*i MIC unsuccessfully attempts division and eventually remains as a single unreduced MIC in one of the two daughter cells. Staining for phosH3(Ser10) (green) highlights only condensed mitotic chromosomes in the WT, while undivided *esp1i* nuclei remain in a permanently condensed state. (B) After 48 hours of *esp1*i induction, MICs become very large and contain ropy structures, suggesting bundles of unseparated chromatids. (C) FISH with a probe against a unique chromosomal locus shows that, whereas the WT MIC contains two copies of the locus, *esp1*i MICs are polyploid. Unlike in the MAC, multiple copies of the locus are not dispersed, but form clusters. This suggests that replicated chromatids do not fall apart but remain in bundles resembling polytenic chromosomes. (D) FACScan analysis after 48 hours of *esp1*i induction confirms that MICs become polyploid due to failing segregation. While MICs and MACs form peaks of different DNA contents in the WT, in the *esp1*i sample they coalesce into a single peak roughly equivalent to the WT MAC DNA content. (E) Meiotic prophase stages are normal but bivalents fail to separate at anaphase I and coalesce back to a single MIC in the absence of Esp1p (compare with the WT situation in Figure 1B). (F) Quantitation of the *esp1*i meiotic arrest 5,5 h after meiosis induction. Bar in (A) represents 10 µm in (A) and (E), bar in (B) represents 10 µm in (B) and (C).

The continuous association of cohesin with mitotic and meiotic chromosomes during and after anaphase led us to ask if the separase cleavage mechanism used in other organisms was conserved in *Tetrahymena*. We therefore designed an RNAi construct to target *Tetrahymena*'s separase homolog, *ESP1*. After induction of the *esp1*i construct in vegetative growth, cells immediately showed problems dividing the MICs. Normally the MIC divides before the MAC (Figure 1A and Figure S3), whereas in *esp1*i cells, MICs arrested in an anaphase-like state by the time the MAC divided (Figure 5A). As a result, the cleavage furrow formed while the MIC was still attempting division, leaving one daughter cell containing the undivided MIC, and one cell without a MIC (Figure 5A). Staining for phosphorylated (Ser10) histone H3, which is a marker for chromosome condensation [47], showed that the MICs stay in a mitosis-like condensation state (Figure 5A). After 24 h of *esp1*i induction, only 10.5% of cells contained MICs, as opposed to 98.5% of uninduced cells. This decreased to 7% after 48 h, and 3% after 72 h of *esp1*i induction (n = 200 cells per genotype and timepoint). After 48 h of *esp1*i induction, the remaining MICs were very large, presumably as a result of polyploidization by numerous rounds of replication and failed separation. Many of these large MICs had a ropy appearance, suggesting a bundled, polytenic state of chromosomes (Figure 5B). FISH staining of a unique chromosomal locus showed signal clusters whose strength indicated that they contained multiple copies, again suggesting a polytenic organization of unseparated chromatids (Figure 5C). To obtain a rough estimate of the degree of polyploidization, FACScan was performed of cultures 48 h after *esp1*i induction. While nuclei from WT cells sorted into two clear peaks corresponding to MICs and MACs, *esp1*i cells showed only one peak for both MICs and MACs (Figure 5D). This indicates that aberrant MICs may reach roughly the same DNA content as MACs and suggests that they may become 16–32-ploid.

When two *ESP1*hp cells were mated and RNAi was induced during meiosis, MICs elongated normally and chromosomes condensed at metaphase I. However, most cells arrested in anaphase I, and chromosomes coalesced back to enlarged undivided MICs (Figure 5E, 5F). Together, these observations suggest that cohesin cleavage does not occur in the absence of Esp1p, which prevents sister chromatid separation in mitosis and the separation of homologs in meiosis I.

## Discussion

### The role of cohesin in chromosome organization

Efficient depletion of cohesin proteins caused problems with MIC chromosome segregation in mitosis, which is suggestive of loss of cohesion. Loss of Rec8p also led to a reduction of cohesion in meiosis. It has been shown in other organisms that cohesion is relatively insensitive to reduced amounts of cohesin [48], [49] whereas even small decreases in the levels of cohesin drastically affect meiotic DNA repair [7], [48], [50], [51]. This explains why meiosis is more affected than mitosis in the *rec8*i and *smc1*i single knockdown strains. Also, cohesin-independent mechanisms, such as relational coiling or catenation of the sister chromatids, protein-protein interactions, or locally delayed replication could provide additional forces to hold sister chromatids together and contribute to proper segregation [52]–[54]. After long periods of cohesin depletion, mitotic cultures deteriorated. However, this was not due to MIC aneuploidy caused by segregation problems, because *Tetrahymena* can propagate with a defective or severely aneuploid MIC for numerous generations [55].

In contrast to MICs, MACs did not display cytologically detectable cohesin. Not even upon artificial DNA damage could we induce notable cohesin loading, although the Rad51 DNA damage response was robust. Nevertheless, depletion of cohesin results in a defect in MAC division. Dividing MACs in cohesin-depleted cells leave behind substantial amounts of DNA in the cleavage furrow, and this DNA remains in the cells as extra-nuclear bodies. This defect, along with the growth defect observed in cohesin depleted cells, may indicate a role of cohesin in normal cell division, either through a direct function in the MAC, or through cross-talk between the MIC and the MAC [56].

### Rec8p and Smc1p are required for meiotic DSB repair and pairing

We have shown that meiotic cells depleted of either Rec8p or Smc1p arrest prior to anaphase I. Similar to the situation in budding yeast [7], [57], [58] and *C. elegans* [8], meiotic DSBs are formed normally in the absence of Rec8p. However, these breaks are not repaired, which likely causes the prophase arrest.

Cohesion has been previously found to be crucial for meiotic DSB repair, to the extent that even a partial loss of cohesin function can result in unrepaired breaks [7], [48], [50], [51]. Exactly how cohesin participates in DNA repair is not known. It is thought that cohesin facilitates repair directed by the sister chromatid by keeping sisters tightly bound together (see [59]). However, one could speculate that close access to the sister chromatid would be less crucial for meiotic DSB repair, which can, and often prefers, to use the homologous chromosome as a template. Therefore, it seems likely that cohesin plays a more direct role in DNA repair than simply providing cohesion, because the repair function is much more sensitive to slight perturbations in cohesin dosage [48]; see [14].

We have also shown that depletion of cohesin in *Tetrahymena* results in a moderate reduction in meiotic pairing. In other organisms, cohesin forms the backbone of the SC, a structure that is central to meiotic recombination (see Introduction). Although *Tetrahymena* does not form an SC, it is quite likely that cohesin plays a role in defining chromosome axes and providing the necessary architecture to support homologous recombination. Precise pairing of homologs in meiosis of *Tetrahymena* is dependent on homologous recombination [23], and so the reduction of pairing in the absence of cohesin may be a result of the defect in DSB repair.

### Cohesin persists on anaphase chromosomes

The persistence of cohesin on anaphase chromosome arms might suggest that dissolution of cohesion works by a mechanism different from kleisin cleavage. However, we have shown that depletion of Esp1p prevents the segregation of chromosomes in both mitosis and meiosis. This phenotype is consistent with the canonical separase-dependent loss of cohesion. There are several possible explanations for the maintenance of Rec8p and Smc1p on chromosome arms: First, it may be related to the absence of an apparent G_1_ interval in *Tetrahymena* and the early onset of MIC DNA replication during late anaphase or telophase [39] (see also [60]). Thus, the permanent presence of cohesins could be due to the temporal overlap of their removal from separating sisters in anaphase and loading to chromatin in preparation for cohesion establishment during replication, shortly after. This explanation may seem less applicable to the association of cohesins with anaphase I chromosome arms, since this division is not followed by DNA replication. However, the two meiotic divisions occur in rapid succession and are followed quickly by post-meiotic replication [60]. Therefore, carrying non-cohesive cohesin along on the chromosomes may allow a more rapid establishment of cohesion. To maintain this “dormant” population of cohesin on chromosomes, there could be a mechanism in which all cohesin rings are opened by separase cleavage of Rec8p and yet continue to bind to chromatin. Because this would require replacement of the cleaved Rec8, it seems more likely that only a small subset of cohesin complexes connect sisters, and only the intersister subpopulation is removed by cleavage. This interpretation was preferred by Tomonaga et al. (2000) [37] for their similar observation in fission yeast mitosis.

### Polytene MIC chromosomes suggest cohesion between multiple sisters

Prolonged depletion of Esp1p in vegetative cells results in many cells without MICs and a few cells with grossly enlarged MICs, presumably as a result of multiple rounds of replication without division. Polyploidization was also found after inactivation of separase in several mouse tissues, in *D. melanogaster* and in non-yeast fungi, whereas budding yeast chromosomes break during attempted separation and daughter cells die as a result [61] (and lit. cit. therein). In *Tetrahymena*, the chromosomes of polyploid MICs appear to be polytenic, because FISH against one chromosomal arm locus shows clustered or banded signals. The embrace model of cohesion limits the number of 10-nm chromatin fibers that can be encircled by a cohesin ring of an estimated diameter of ca. 40–45 nm to about four to six. This is hardly consistent with our observation of ∼4 chromatid bundles and the estimation of 16–32-ploid chromatid content. Thus, chromatid bundles may be formed by cohesin rings randomly encircling sisters as they are replicated within the bundle, so that a large, networked, multi-sister bunch is created, instead of individual pairs of sisters. Alternatively, some form of non-embrace-type of cohesion could be employed (see [1]).

### *Tetrahymena* possesses a variant of the canonical cohesion-segregation machinery, with a single kleisin

We have found that *Tetrahymena* possesses components of the canonical cohesion-segregation machinery characterized in other model systems such as *Saccharomyces cerevisiae*, *Schizosaccharomyces pombe*, *Arabidopsis* and mice. Interestingly, only one α-kleisin homolog was found in *Tetrahymena*, which functions in both mitosis and meiosis. In contrast, most other organisms (with the possible exception of *Drosophila* [62]) have two paralogs, e.g. the mitotic Scc1/Mcd1 and meiotic Rec8 of yeast. Mammalian systems even have three homologs: Rad21, Rec8, and Rad21L, with the latter two being meiosis specific [63]–[65]. One reason for having only one multipurpose α-kleisin in *Tetrahymena* is its lack of a dedicated pre-meiotic S phase [66], [67]. Cells go from G_2_ alternatively into mitosis or meiosis, and therefore there is no opportunity to load a specific cohesin.

Special features in the meiotic kleisin have been considered important for maintaining cohesion of sister centromeres in meiosis I so that they segregate together. This is in contrast to the mitotic cohesin, which ensures sisters segregate to opposite poles (for review see [5]). It will be interesting to learn how *Tetrahymena* Rec8p performs both functions. On the other hand, meiotic Rec8 can take over most mitotic cohesin function in budding yeast [27]–[29]. Thus, the primary question may not be how *Tetrahymena* can make with a single α-kleisin, but rather why budding yeast and the others require a mitotic and a meiotic version. It was found that Scc1, but not Rec8, can induce DSB-dependent cohesion in mitosis [68], and it is conceivable that Scc1 also performs better in non-canonical cohesin roles such as gene regulation. Thus, a specialized kleisin may have diverged during evolution to be optimized for functions that may be of subordinate importance in *Tetrahymena*, due to its allocation of gene expression and propagation to different nuclei.

Clearly, the function of cohesin in *Tetrahymena* has many parallels with previously studied organisms. However, future studies are needed to explore the numerous differences that we have found, including *Tetrahymena*'s use of a single α-kleisin and the continuous association of cohesin with chromatin throughout anaphase.

## Materials and Methods

### Strains and cell culture

*Tetrahymena thermophila* strains B2086 and Cu428 served as wild types and as the source strains for transformation with RNAi constructs. Cells were propagated vegetatively at 30°C according to standard methods (see [69]). For meiosis experiments, cells were grown to a density of ∼2×10^5^ cells/ml and made competent for conjugation by starvation in 10 mM Tris–Cl (pH 7.4) for at least 16 h. Conjugation and meiosis were induced by mixing starved cultures of different mating types.

### RNAi gene knockdown

The *ESP1*hp construct was created by amplifying a ∼500 bp region of the *ESP1* ORF and cloning it into the rDNA based RNAi vector to create a hairpin expression cassette [45] (see Table S2 for primer sequences). Transformation and selection was performed as previously described [38].

For *REC8* RNAi, the transformation strategy was changed slightly. A new vector was created using pBS-CHX, a vector targeting knock-ins to the *RPL29* gene which also confers cycloheximide resistance (gift of Chad Pearson). The hairpin expression cassette was released from the rDNA vector using NotI digestion and cloned into the NotI site of the pBS-CHX multiple cloning site (MCS). Then the XmaI site in the MCS was destroyed by blunting and religation in order to allow direct cutting and pasting of hairpin fragments into the PmeI/XmaI and ApaI/XhoI halves of the expression cassette. An ∼500 bp fragment of the *REC*8 gene was then amplified from genomic DNA using primers that added the appropriate restriction sites, and these fragments were cloned into the two sides of the hairpin cassette. For a map of vector REC8hpCYH see Figure S6. This construct was digested with BlpI and introduced into vegetatively growing *Tetrahymen*a by biolistic transformation. Cells were grown overnight, then selected in 7.5 µg/ml cycloheximide. Transformants were successively grown in higher concentrations of cycloheximide up to 20 µg/ml, to increase the macronuclear copy number of the hairpin containing chromosome.

In order to create a strain for double RNAi of both *REC*8 and *SMC*1, it was necessary to use a different selection marker for the *SMC1*hp, therefore another RNAi vector was created using pMNMM3 (gift of Kazufumi Mochizuki), which carries the *NEO5* (paromomycin resistance) gene and targets knock-ins to the *MTT1* locus, utilizing the *MTT1* cadmium-inducible metallothionein promoter for expression. To facilitate cloning into this vector, a new hairpin linker was amplified from genomic DNA using primers that introduced BamHI and PmeI restriction sites on one end, and XmaI and PstI sites on the other end. This was cloned into the MCS of pMNMM3, and then the ∼500 base pair fragment of *SMC1* was amplified and cloned into either end of the linker as in previous constructs. For a map of vector SMC1hpNEO see Figure S6. The construct was digested with NotI and XhoI and introduced into vegetatively growing *Tetrahymena* (either WT strains or *REC8*hp strains) by biolistic transformation [70]. Transformants were selected in 120 µg/ml paromomycin, then grown in successively higher concentrations of drug, up to 2 mg/ml.

The *SPO11*hp construct was prepared in the same way as the *REC8*hp, using the primers listed (Table S2). Transformation and selection was also performed as for *rec8*i. In all cases, RNAi was induced by expression of dsRNA from the *MTT1* promoter by the addition of 0.2 µg/ml of CdCl_2_ (final concentration:) to cells carrying the hairpin construct.

### Protein tagging

Rec8-GFP, Smc1-HA and Scc3-mCherry expressing cells were created using a knock-in approach to fuse the tag to the C-terminus of each gene at its native genomic locus. Tagging constructs were created by amplifying the last ∼500 bp of the ORF as well as a ∼500 bp region downstream of the gene, and fusing these two products with the tagging cassette using overlapping PCR. (See Table S2 for primer sequences). The tagging cassettes were amplified from pHA-Neo4 or pEGFP-Neo4 (gifts of Kazufumi Mochizuki). Tagging constructs were introduced into the MAC of vegetatively growing B2086 and Cu428 cells by biolistic transformation. Transformants were selected in media containing paromomycin in increasing concentrations up to 50 mg/ml. For detecting Rec8-GFP by Western blotting, protein extracts were prepared by trichloroacetic acid precipitation, run on 10% SDS-PAGE gels and blotted. Membranes were incubated with anti-GFP antiserum and with appropriate HRP-conjugated secondary antibody, and the protein bands were detected by chemiluminescence.

### Protein immunoprecipitation and mass spectrometry

Immunoprecipitation against GFP tag was performed using magnetic GFP-trap beads (ChromoTec, Martinsried, Ger), according to the manufacturer's protocol. Lysates were created by sonicating 5×10^7^ mating cells (5 h after mixing) in 2 ml of lysis buffer +1 mM PMSF and protease inhibitors (1× cOmplete mini, Roche, Indianapolis, IN), 4×25 sec at 37% power, duty cycle 5. After 2 h incubation with lysate, the beads were washed 7×5 min with wash buffer (10 mM Tris/Cl pH7.5, 150 mM NaCl, 0.5 mM EDTA), and 2×5 min with 150 mM NaCl. The bead bound proteins were trypsinized, and peptides were loaded on a Dionex UltiMate 3000 HPLC system (Thermo Scientific, San Jose, CA). Peptides separated in a 0.1% formic acid/0% acetonitrile – 0.08% formic acid/80% acetonitrile gradient in water were injected into the mass spectrometer via an electrospray-interface. MS/MS analysis was carried out with a Q Exactive mass spectrometer (Thermo Scientific), and peptide spectra were recorded over a mass range of 350–2000 m/z (for details see [71]).

For peptide identification, the .RAW-files were loaded into Proteome Discoverer 1.4.0.282 (Thermo Scientific). MS/MS spectra created were searched using Mascot 2.2.07 (Matrix Science, London, UK) against the NCBI non-redundant protein sequence database, using the taxonomy group Alveolata.

### DNA damage assay

For induction of DNA damage by UV irradiation, 5 ml aliquots of cells were placed in a 90 mm plastic Petri dish. Open dishes were placed in a Stratalinker crosslinker and treated with 254 nm UV (UV-C) at a dosage of 150 Joules/m^2^. Treatment with ionizing radiation was performed by exposure to 5000 rads of γ-radiation from a ^137^Cs source. For chemical induction of DNA damage, cells were treated with 100 µg/ml cisplatin (from a 2 mg/ml stock solution in 10 mM Tris-HCl), 50 µg/ml bleomycin (from a 10 mg/ml stock solution in 10 mM Tris-HCl) or 4 mM methyl methane sulfonate (MMS, from a 100 mM stock solution in 10 mM Tris-HCl). Cells were fixed and prepared for immunostaining (see below). Immunostaining of DNA repair protein Rad51 allowed cytological detection of DNA damage in the MAC.

### Cell preparation for FACScan analysis

Cell suspensions were centrifuged and Carnoy's fixative (methanol, chloroform, acetic acid 6∶3∶2) was quickly added to the pellet. This disrupts the cells and separates MICs and MACs. Carnoy's fixative was replaced by 70% ethanol after 1 h at room temperature, and the fixations were stored in the freezer. Shortly before measuring, the nuclei were pelleted by centrifugation, resuspended in 1× PBS and stained by the addition of DAPI (4′,6-diamidino-2-phenylindole; final concentration: 0.2 µg/ml).

### Cytological preparation, immunostaining, and fluorescence in situ hybridization (FISH)

Different preparation methods were applied for subsequent immunostaining and FISH. A combined formaldehyde fixation and detergent permeabilization treatment [72] was used for subsequent chromosome staining with DAPI or for immunostaining of Dmc1p/Rad51p, PhosH3, and GFP and HA tags. An enforced detergent spreading method for the removal of free nuclear proteins [44] was applied for probing chromatin associated proteins. For immunostaining of γ-H2A.X, cells were fixed with Schaudinn's fixative, washed and resuspended in methanol [73]. For subsequent FISH, cells were fixed with Carnoy's fixative (methanol, chloroform, acetic acid 6∶3∶2). Drops of fixed cell suspensions were dried down on slides.

For immunostaining, slides were washed with 1×PBS and 1×PBS+0.05% Triton, incubated with primary antibodies over night at 4°C, washed again, incubated with appropriate FITC- or Cy3-labeled secondary antibodies for 2 h, and washed and mounted with anti-fading buffer supplemented with 0.5 ìg/ml DAPI. The following primary antibodies were applied: Mouse monoclonal antibody against the related DNA repair proteins Dmc1 and Rad51 (1∶50, Clone 51RAD01, NeoMarkers, Fremont, CA), mouse anti-γ-H2A.X antibody (1∶200, BioLegend, San Diego, CA), rabbit anti-phosphorylated H3Ser10 (1∶500, Upstate Biotechnology, Charlottesville, VA), rabbit anti-GFP (1∶100, Molecular Probes, Eugene, OR), monoclonal mouse anti-HA (1∶50, Roche Diagnostics GmbH, Mannheim, GER) and rabbit anti-HA (1∶100, Sigma St. Louis, MO).

For FISH, a probe against an intercalary chromosomal region, scaffold scf_8254686 (http://ciliate.org), the same as described in ref. [23], was used. DNA on slides and the Cy3-labeled hybridization probe were denatured and hybridized for 36–48 h at 37°C [72].

In all cases, chromatin was counterstained with DAPI, and z stacks of pictures were taken under a fluorescence microscope equipped with the appropriate filters. Picture stacks were deconvolved, projected, assigned false colors, and multicolor images were merged.

### Detection of DSBs by pulsed-field gel electrophoresis (PFGE)

For details of the detection of DSB-generated fragments by PFGE, see [44]. In short, chromosome-sized DNA was prepared in agarose plugs. The run was performed in 1% agarose with 0.5× TBE buffer at 200 V, 6°C for 14 h with 60-s pulses, 10 h with 90-s pulses, and 1 h with 120-s pulses in a Bio Rad Chef-DR III system. Under these conditions, intact MIC chromosomes do not enter the gel, whereas fragments migrate as a single band. Since numerous small MAC chromosomes are distributed along the entire gel and cover the DSB-generated signal, MIC-borne DNA fragments were highlighted by Southern detection of a MIC-specific DNA [44]. The membrane was stripped and re-hybridized with a probe against a 121-kb MAC chromosome as a marker to test equal DNA loading and Southern transfer for different time points.

## Supporting Information

Figure S1 Alignment of the conserved N-terminal and C-terminal domains of α-kleisins. A, B: ciliate members of the family, C-G and I-M: mitotic kleisins, N-V: meiotic kleisins, W: bacterial kleisin. Mammalian Rad21L (H) is exceptional because it is very similar the mitotic kleisin Rad21, but functions in meiosis (see main text).
(PDF)

Figure S2 Smc1 and Smc3 peptides identified by mass spectrometry.
(PDF)

Figure S3 A. Complete series of MIC mitotic stages stained for Rec8-GFP and Smc1-HA. At no time during mitosis is the MIC devoid of Rec8 (red) or Smc1 (green). Also, notice that cohesins are not detectable in the MAC. B. Detection of MIC DNA replication by BrdU incorporation. MIC DNA synthesis starts immediately after the separation of daughter nuclei and prior to the splitting of the MAC. No BrdU signal was present in cells with one or two-spindle-shaped MICs (n = 30), whereas weak BrdU signal (orange) appeared when daughter MICs were drop-shaped. BrdU signal was present in 100% (n = 50) of dividing cells with round daughter MICs. BrdU was added 15 min prior to fixation. Incorporated BrdU was detected with an anti-BrdU antibody [72]. Mitotic stages were enriched by the synchronization of vegetative cultures. Cells were starved over night, re-fed and fixed 4 h after feeding.
(PDF)

Figure S4 Grayscale images of Rec8-GFP and Smc1-HA of Figure 3C and 3D. This presentation allows better visualization of protein present along the arms of meiotic anaphase chromosomes.
(PDF)

Figure S5 *smc1*i cells display mitotic and meiotic phenotypes. (A) In vegetatively dividing cells, MIC mitosis shows delays. Daughters are not separated when the MAC splits, which is not the case in the WT (compare Figure 1A). Also, DNA masses are left between newly split MACs. (B) In meiosis, the *smc1*i cell arrests at an abnormal metaphase-anaphase I stage, whereas the WT partner progresses normally through the meiotic divisions. (C) To confirm that it was indeed the knockdown partner displaying the cytological defect, matings were performed between *smc1*i cells and Rec8-GFP cells (serving as internal WT controls). Always, the Rec8-GFP cells were the ones that showed normal meiotic divisions. The same control was made for *rec8*i×Rec8-GFP matings (not shown). Bar: 10 µm.
(PDF)

Figure S6 Vector constructs for *rec8* RNAi and *smc1* RNAi.
(PDF)

Table S1 Proteins identified by mass spectrometry analysis of Rec8-GFP immunoprecipitation.
(PDF)

Table S2 List of oligonucleotides.
(PDF)

Text S1 Search for α-kleisin homologs and Scc3 homologs. Description of the bioinformatic methods used to identify cohesin homologs in *Tetrahymena*.
(PDF)
