# Supplementary material for: A Single Cohesin Complex Performs Mitotic and Meiotic Functions in the Protist Tetrahymena
Source: PLoS Genet. 2013 Mar 28;9(3):e1003418. doi: 10.1371/journal.pgen.1003418 (PMC3610610; doi:10.1371/journal.pgen.1003418)
Supplement: Figure S1 — Alignment of the conserved N-terminal and C-terminal domains of α-kleisins. A, B: ciliate members of the family, C-G and I-M: mitotic kleisins, N-V: meiotic kleisins, W: bacterial kleisin. Mammalian Rad21L (H) is exceptional because it is very similar the mitotic kleisin Rad21, but functions in meiosis (see main text). (PDF) [file pgen.1003418.s001.pdf]

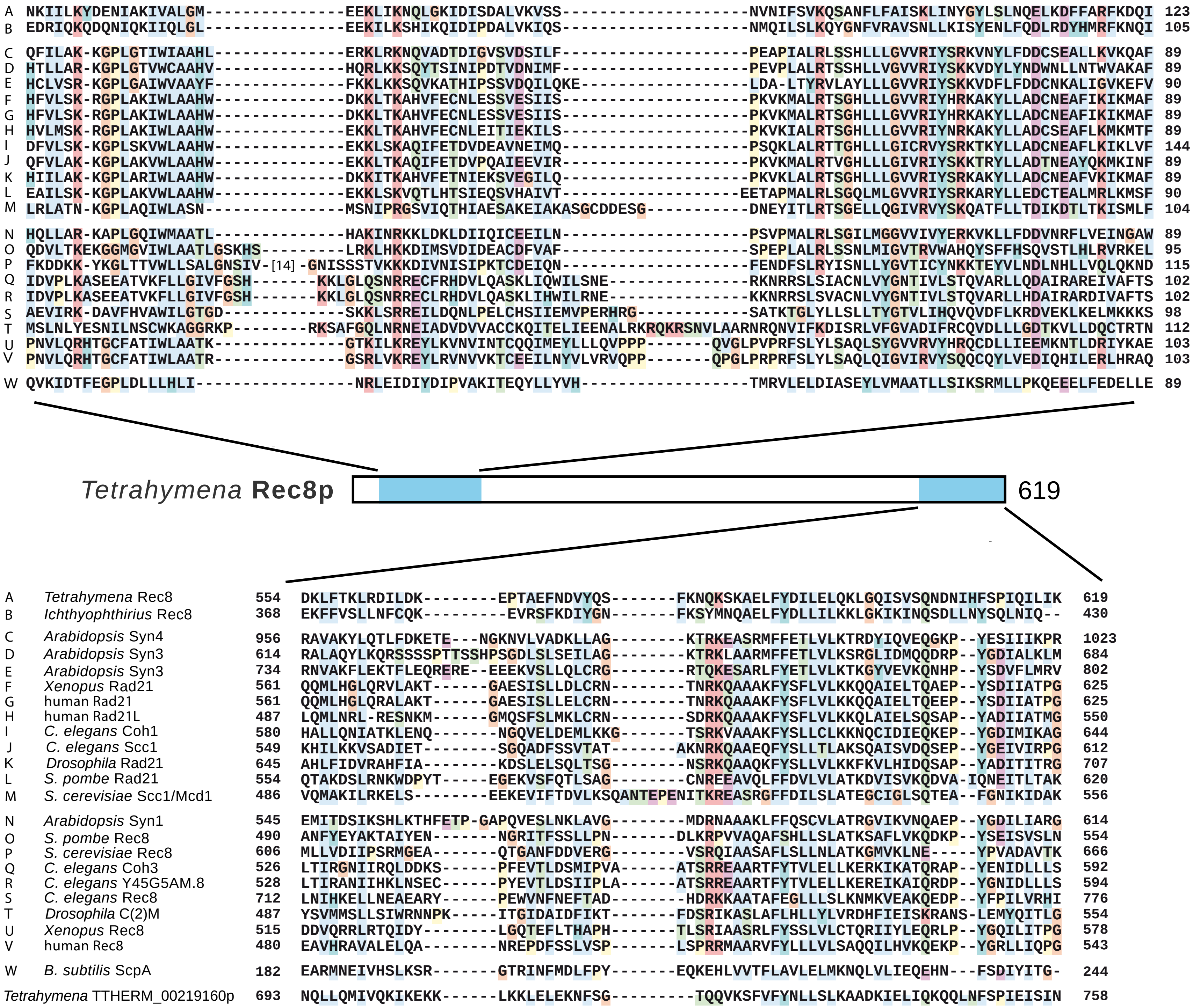
Tetrahymena Rec8p
619
A Tetrahymena Rec8 554 619
B Ichthyophthirius Rec8 368 430
C Arabidopsis Syn4 956 1023
D Arabidopsis Syn3 614 684
E Arabidopsis Syn3 734 802
F Xenopus Rad21 561 625
G human Rad21 561 625
H human Rad21L 487 550
I C. elegans Coh1 580 644
J C. elegans Scc1 549 612
K Drosophila Rad21 645 707
L S. pombe Rad21 554 620
M S. cerevisiae Scc1/Mcd1 486 556
N Arabidopsis Syn1 545 614
O S. pombe Rec8 490 554
P S. cerevisiae Rec8 606 666
Q C. elegans Coh3 526 592
R C. elegans Y45G5AM.8 528 594
S C. elegans Rec8 712 776
T Drosophila C(2)M 487 554
U Xenopus Rec8 515 578
V human Rec8 480 543
W B. subtilis ScpA 182 244
Tetrahymena TTHERM_00219160p 693 758
