# Supplementary material for: A Single Cohesin Complex Performs Mitotic and Meiotic Functions in the Protist Tetrahymena
Source: PLoS Genet. 2013 Mar 28;9(3):e1003418. doi: 10.1371/journal.pgen.1003418 (PMC3610610; doi:10.1371/journal.pgen.1003418)
Supplement: Figure S2 — Smc1 and Smc3 peptides identified by mass spectrometry. (PDF) [file pgen.1003418.s002.pdf]

Rec8 Sequence Coverage: **23%**

Matched peptides shown in **Bold Red**

1 MVRQQNNQNT QSQSNITTSV SQYTSEDSQN SQVQRENRNK IILK**YDENIA**
51 **KIVALGMEEK** LIKNQLGK**ID** **ISDALVKVSS** **NVNIFSVK**QS ANFLFAISKL
101 INYGYLSLNQ ELKDFFARFK DQIMKQQQQQ QQKEKQHRSS RKNQTKKRLR
151 DAAK**EIADNL** **SNSIDERSNT** **SIQQPR**HIST EERLNMQEQN LISPLAMMIN
201 QRSDLDSDNE NSNTGFSDNL PDPNQQRLMM LGEDNLAGIE FDGGLGMDTG
251 FDYDGFQYDF SEQDSQSQGF NKVNGNAINI SQELNQVGDI SERYIRKITN
301 YANQQSSKKK KKTKQKQQGH NKEGEHAILN ETVDMDNQEF SKAMDEKLNQ
351 FYSSQSQRVN KNISHISDIS RLSTTLQQPS ILSQNFNIYE ESISEAPFQQ
401 INISNYLNAS TLVDQPILNL TKKFDEAISL KENDNNVLLS EELISKLEK**A**
451 **MQNINIDLVK** QDQGGVDMMD FNQDNQGGYM DYPDDFGSLD QYNQQKEEQD
501 GELEIPVQIP EIQKSKLEKD K**KIDEIDSLQ** **DFDITPLDKK** **ILTEEEQRVL**
551 **QQYDK**LFTK**L** **RDILDK**EPTA EFNDVYQSFK NQK**SKAELFY** **DILELQK**LGQ
601 ISVSQNDNIH FSPIQILIKM VSKGEELFTG VVPILVELDG DVNGHK**FSVS**
651 **GEGEGDATYG** **K**LTLKFICTT GKLPVPWPTL VTTLTYGVQC FSRYPDHMKQ
701 HDFFK**SAMPE** **GYVQERTIFF** **KDDGNYK**TRA **EVKFEGDTLV** **NR**IELK**GIDF**
751 **KEDGNILGHK** **LEYNYNSHNV** **YIMADK**QKNG IKVNFKIRHN IEDGSVQLAD
801 HYQQNTPIGD GPVLLPDNHY LSTQSALSKD PNEKRDHMVL LEFVTAAGIT
851 LGMDELYKTS

Smc1 Sequence Coverage: **56%**

Matched peptides shown in **Bold Red**

1 MNIYFIEVEN FKSFRGK**HQI** **GPFTQMTGII** **GPNGCGK**SNI VDALTFAFNI
51 ENARNHHPIS SITQQSKPDM CSVEVVLQDK RQKISFKKTQ NRKKQITFYM
101 NNNILNQDQY LEQLK**KYNIG** **PQSFMLQGET** **DK**LIK**KSPEE** **LSEIIEKACG**
151 **SLQYKKEYDE** **LNQQIKSIND** **ETIK**IISEQK NLKKEDQKLR SISEQNEK**YE**
201 **SLNEEIK**QIE VK**IEQANFFQ** **IDSSILQEMK** ALSTTQNKLD ERKTEEGAIK
251 QK**IQQNNIK**I KNLQKEQSKK EKEREQIKQQ IR**ELQEQLEQ** **EEDQVK**NAKS
301 FSENAKLQK**S** **FQDQIKDIND** **SLAKLNGQLR** **KEESILNDMQ** **SK**ESQKSKIE
351 NKK**LKDNYEE** **YQELVRVCNS** **QTLNLELESK** **KLANDMNNAL** **SQLKVSIEAQ**
401 **SKQNNHIEEL** **ETK**INEAKTN QISKQGELQQ K**QQEISSIKQ** **ELIELENQR**K
451 DNVLK**MENIQ** **KDLSVLLSNK** **LSLEYELEQM** **EEIR**NQNKVY RELHKFK**GFY**
501 **GQFSELIR**NN SNK**SYDLSIK** IALGSFLSCL VVDSPQTASL VNVTLASQGL
551 AR**DVMILQNL** **PNSDDDLVK**A R**IGNLGELAT** **SLVDIDERSS** **KYEENPELK**A
601 KIADTLKYLL RGK**VICENTE** **K**AFQLRNRKI K**EVYQIITKD** **GDIVTSGSIQ**
651 **ISSSDK**EKYS K**KDLGTEQNI** **QNLQK**KIQNE EKQIDK**KMVE** **LESIK**NSDTE
701 KKYNK**LIEQL** **QVRESSIEIT** **NTELTSIQEK** IKKLEEKKQQ IKNVIAK**SLE**
751 **IINLENTVK**E IEK**RLQDTQE** **K**IDKK**RSEVY** **DPFCK**KYKID IK**ELISVNAD**
801 **TVER**IFKNIE KQQTK**VQDIN** **NSIK**SHQEKL **DNIIK**AQEKY NENK**KNFEEL**
851 **LEQFK**NVSTK IQNTKNELDN KYK**KAISEVQ** **QFEKEIQEIN** **QENESSYQLL**
901 **IQQPNQLRK**D IIQIK**HSIGQ** **LIDKKETLWQ** **ECQLK**QIEFK DSSKMNNK**AQ**
951 **NDSMMEELDS** **K**SK**SQSSQQD** **DIESQDEKEQ** **IQTLSK**DFTK SLK**QYTQALS**
1001 **QINDVKLQES** **LNKFSEIEML** **NYFMLFK**DKS QFQKKRTVR**Q** **QQMVDETK**ES
1051 LLTQLLETVD IGSLLNYQNR **LVDRLEELR**K **KIEEYASKDL** **AEGLMGATVT**
1101 **SEEMK**VKKEN LKNR**QAELVS** **EKKKAEEDLK** NIKKLRK**EKF** **DEFFK**EVQSQ
1151 VK**IYYGELTK** LKGKHQGQK**G** **NADLTVSDEE** **EK**YK**GGINYF** **CCPPKKAYAL**

1201 **NPEK**ELSGGE KTLAQIALFL AIAKDSPFLI LDESEAALDS SNTQNVIETF
1251 RNLITKQQK**L IVSHNQEVYS QCDSLIGTTF NR**YTDTSFTL SLNLKA

Smc3 Sequence Coverage: **47%**

Matched peptides shown in **Bold Red**

1 MSIKSISIEK FKSYSNCK**IE GLSDNINILY GK**NGSGKSNL LAGTQKILLR
51 NLAFNIFFLL KLALSFISSD QYDRADRAQL MKNYK**EGDPK VEIELDNLVK**
101 IDKKDMEDEP KAQKTSGDSV LLSRTLEQRE NVYRVDDRVV NLNQFNNIFE
151 CFGLSRK**NPF TIVAQNRIQE ISNMSDSEIY KLLQQVAGIE AFMNK**QEK**AE**
201 **ELFEK**AQREK **EGIDQSLQEI EDK**ISQLSGE K**EQYAQNQEQ EIIIQSLQSI**
251 **YYDKLKEELQ K**K**KLECNK**EI NNQKLQEEKQ K**LQDLQNQK**E AK**NEELLR**FK
301 EKLQR**LTVIE EQVKSDQMNL QNKVNSLEQN IQK**LKSIKTN KDEKEIEARL
351 K**EVNIQCTK**L QK**DLDEVIDK** ITNEKDGIDT K**QQEYEQLVL R**EKFKTQQEK
401 **QSYFNQR**IQD IKQSISEKSQ TQQNLKEKVK KSEQR**LNLVN QEIEKLQNQI**
451 **NSFQTESNK**A QKTINDKKNQ IQKLRQEQLE KRKTINDK**KI ELEDLNNQIK**
501 **KRVDDIQK**QL K**SGLIQAIEQ IEESIQKEGI QGYK**GLLIDH LQFNSALTYA
551 YDIAQKVDNQ NTASLIIDIV KKLKIK**TSNL NIIPLDWVED DIDDEKENQI**
601 **AGFGFQK**LFD TRYVK**AKGDH ADDIKR**LAKN IFEKYGVIK**E YEHALEVASQ**
651 **FK**VNCITEKR QIVYSGGFIS HVGFCTVAKV DK**ITLYNEYK** SFKAKKDAIA
701 KEIEQLEK**SM NDIQLQQNDI NSNVSK**EQAS LAK**QSNTIFM NR**QLIKSSEV
751 EK**KVLTDELN ETNQLLQSFS QQLK**DLEQEK **IGLENTSK**SS K**FEPLNQEDK**
801 QKMYK**LQEEI K**SKTTVLQKS LKEKQNVEKE YNK**YRNEEQQ LTNHLSQLSE**
851 **GGGMKDGEVL FQSIDQRISS SEQDLKHQES ILK**AQKAIVE KATQSK**KTLE**
901 **EQIK**KIEGES QDKLKEQIQS QQELCNNLEQ QISALLLRKQ QYISK**EYEID**
951 **QK**K**ELLVVSP DFIQK**NSHLS KDNLKDKLNR **ITQQTTIKK**Y TAKDKILFEK
1001 LK**DLDEKYEE YK**SK**YTELTK** NEEQAKNLID QFK**SKCEQTI K**MAFSKFEHY
1051 MKNYFK**QISS AGTIDVK**LMH NHEISISVAF KQTTNGIFKA SKKKTKKTNR
1101 KELSGGEKSC LAILILMALQ RCDPAPYYIF DEFDAALDAG YIRPIAK**IIS**
1151 **ENSQKSQFLI VSHKFEFLQA LQQENCK**CFQ ISLKDKISEI EEKTLSAAIN
1201 FVKNG
