# Supplementary figures and images for: A Single Cohesin Complex Performs Mitotic and Meiotic Functions in the Protist Tetrahymena
Source: PLoS Genet. 2013 Mar 28;9(3):e1003418. doi: 10.1371/journal.pgen.1003418 (PMC3610610; doi:10.1371/journal.pgen.1003418)

A

Rec8

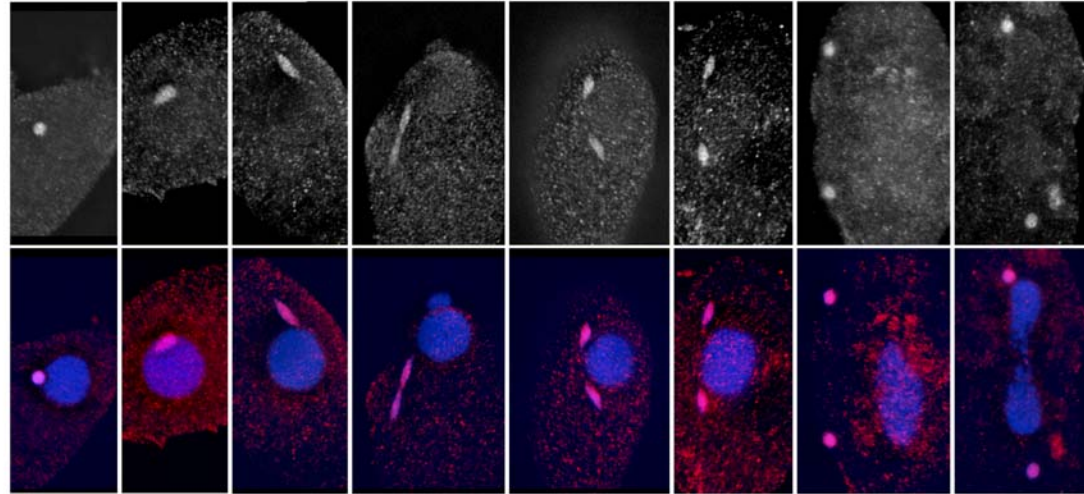

Smc1

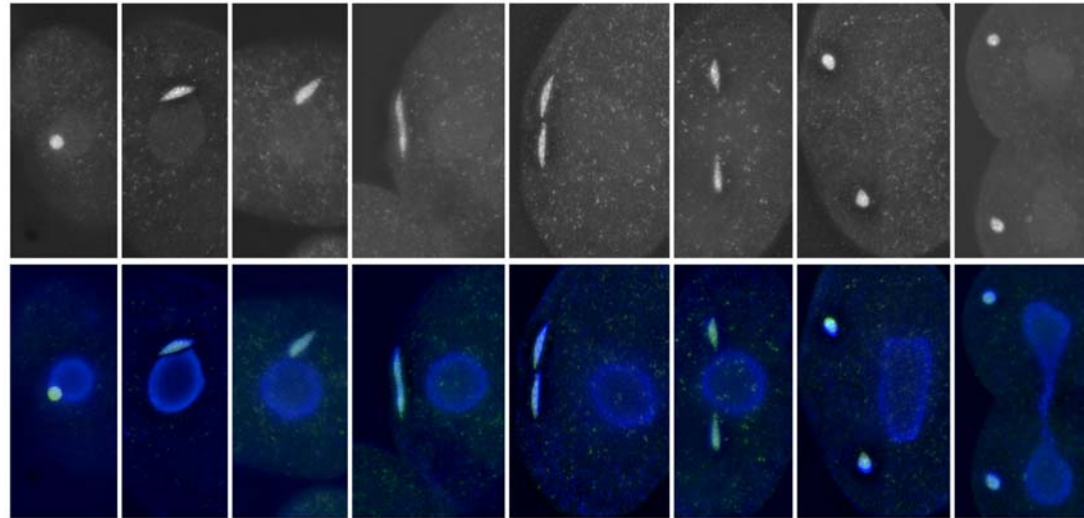

B

BrdU

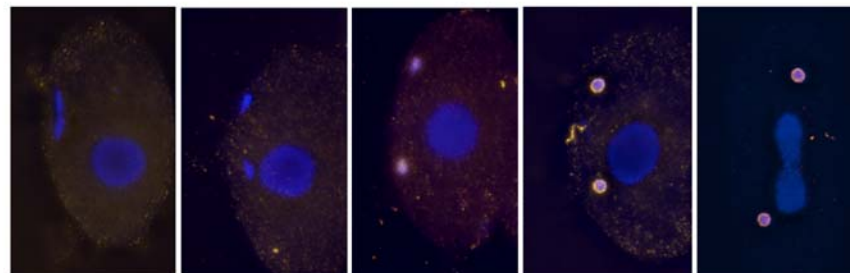

Supplement: Figure S3 — A. Complete series of MIC mitotic stages stained for Rec8-GFP and Smc1-HA. At no time during mitosis is the MIC devoid of Rec8 (red) or Smc1 (green). Also, notice that cohesins are not detectable in the MAC. B. Detection of MIC DNA replication by BrdU incorporation. MIC DNA synthesis starts immediately after the separation of daughter nuclei and prior to the splitting of the MAC. No BrdU signal was present in cells with one or two-spindle-shaped MICs (n = 30), whereas weak BrdU signal (orange) appeared when daughter MICs were drop-shaped. BrdU signal was present in 100% (n = 50) of dividing cells with round daughter MICs. BrdU was added 15 min prior to fixation. Incorporated BrdU was detected with an anti-BrdU antibody [72]. Mitotic stages were enriched by the synchronization of vegetative cultures. Cells were starved over night, re-fed and fixed 4 h after feeding. (PDF) [file pgen.1003418.s003.pdf]

*smc1i* vegetative division

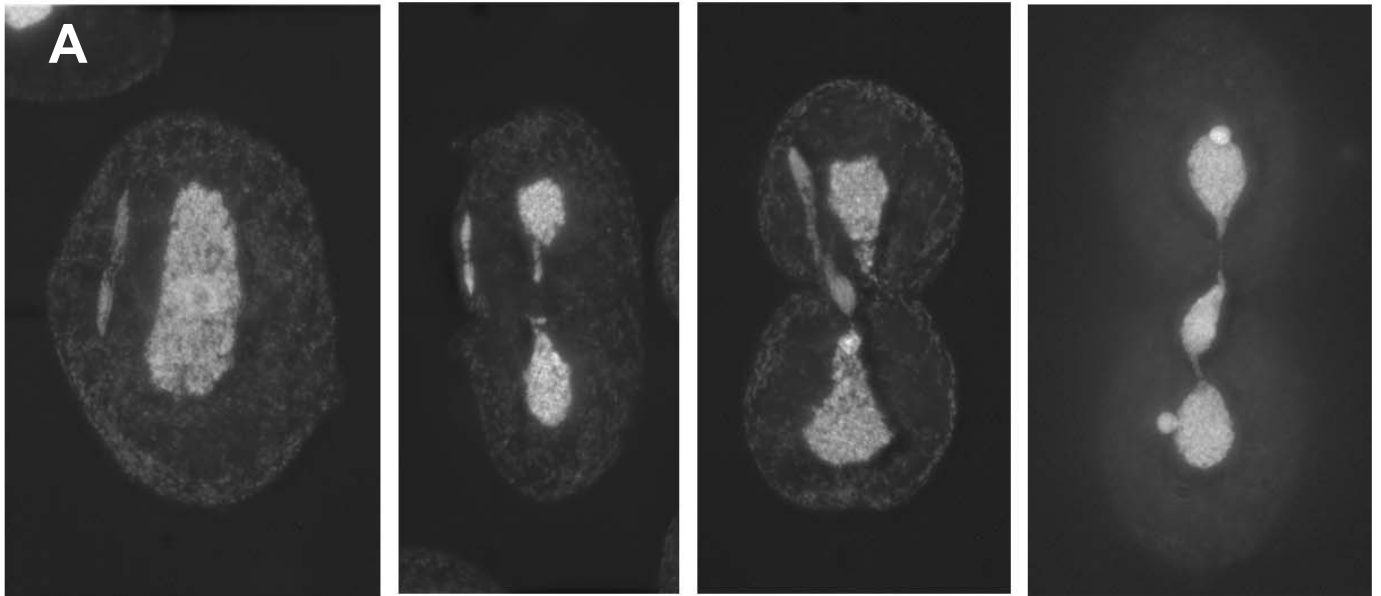

*smc1i* meiosis

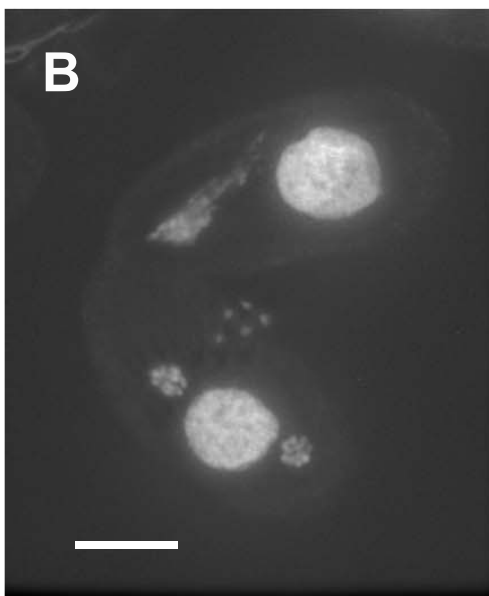

WT

*smc1i*

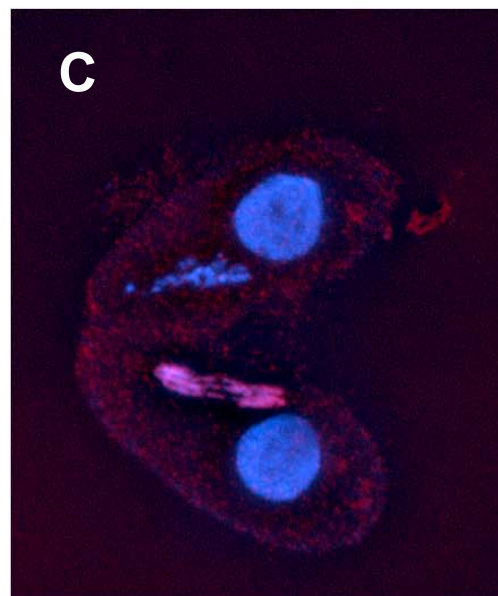

Rec8-GFP

*smc1i*

Supplement: Figure S5 — smc1i cells display mitotic and meiotic phenotypes. (A) In vegetatively dividing cells, MIC mitosis shows delays. Daughters are not separated when the MAC splits, which is not the case in the WT (compare Figure 1A). Also, DNA masses are left between newly split MACs. (B) In meiosis, the smc1i cell arrests at an abnormal metaphase-anaphase I stage, whereas the WT partner progresses normally through the meiotic divisions. (C) To confirm that it was indeed the knockdown partner displaying the cytological defect, matings were performed between smc1i cells and Rec8-GFP cells (serving as internal WT controls). Always, the Rec8-GFP cells were the ones that showed normal meiotic divisions. The same control was made for rec8i×Rec8-GFP matings (not shown). Bar: 10 µm. (PDF) [file pgen.1003418.s005.pdf]

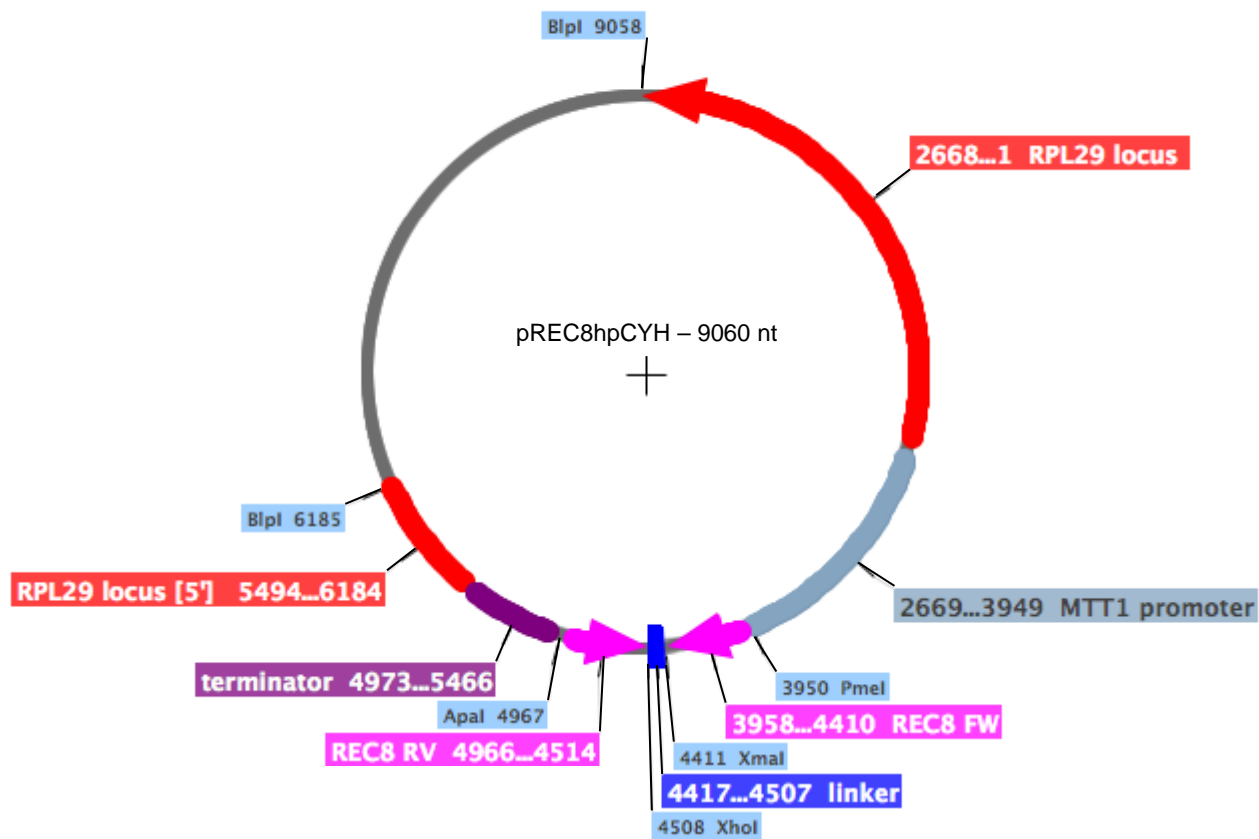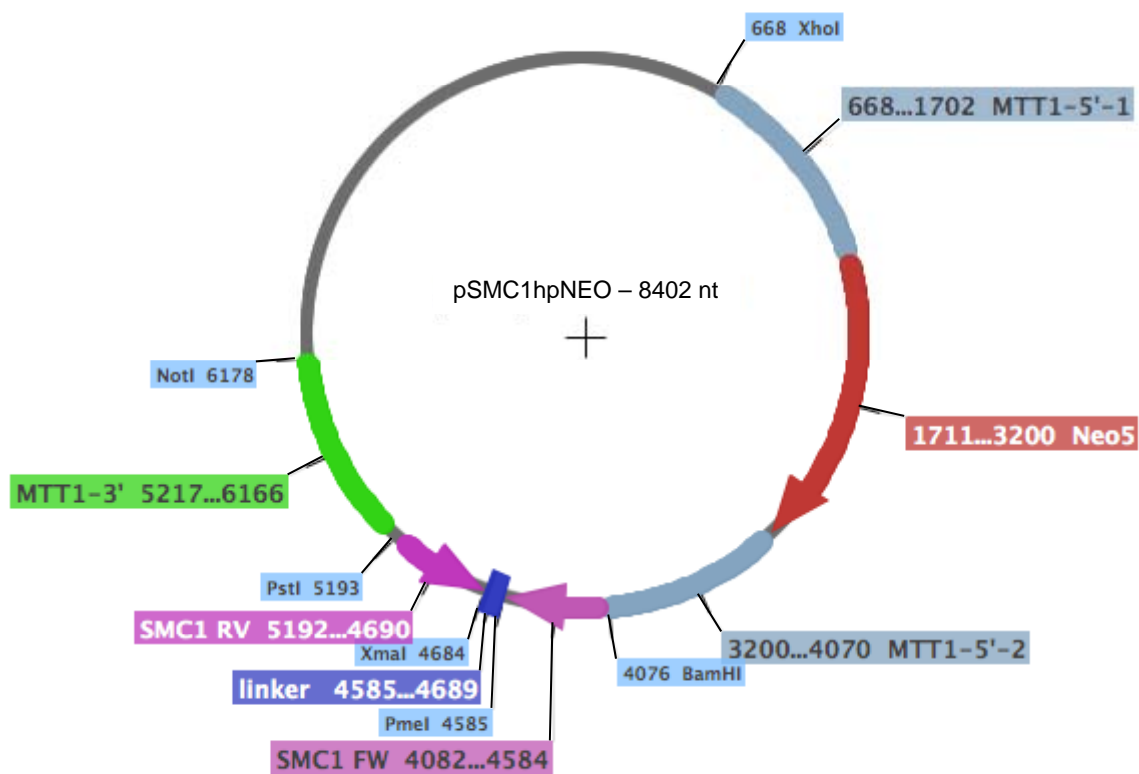

Supplement: Figure S6 — Vector constructs for rec8 RNAi and smc1 RNAi. (PDF) [file pgen.1003418.s006.pdf]
