# Supplementary material for: A Single Cohesin Complex Performs Mitotic and Meiotic Functions in the Protist Tetrahymena
Source: PLoS Genet. 2013 Mar 28;9(3):e1003418. doi: 10.1371/journal.pgen.1003418 (PMC3610610; doi:10.1371/journal.pgen.1003418)
Supplement: Figure S4 — Grayscale images of Rec8-GFP and Smc1-HA of Figure 3C and 3D. This presentation allows better visualization of protein present along the arms of meiotic anaphase chromosomes. (PDF) [file pgen.1003418.s004.pdf]

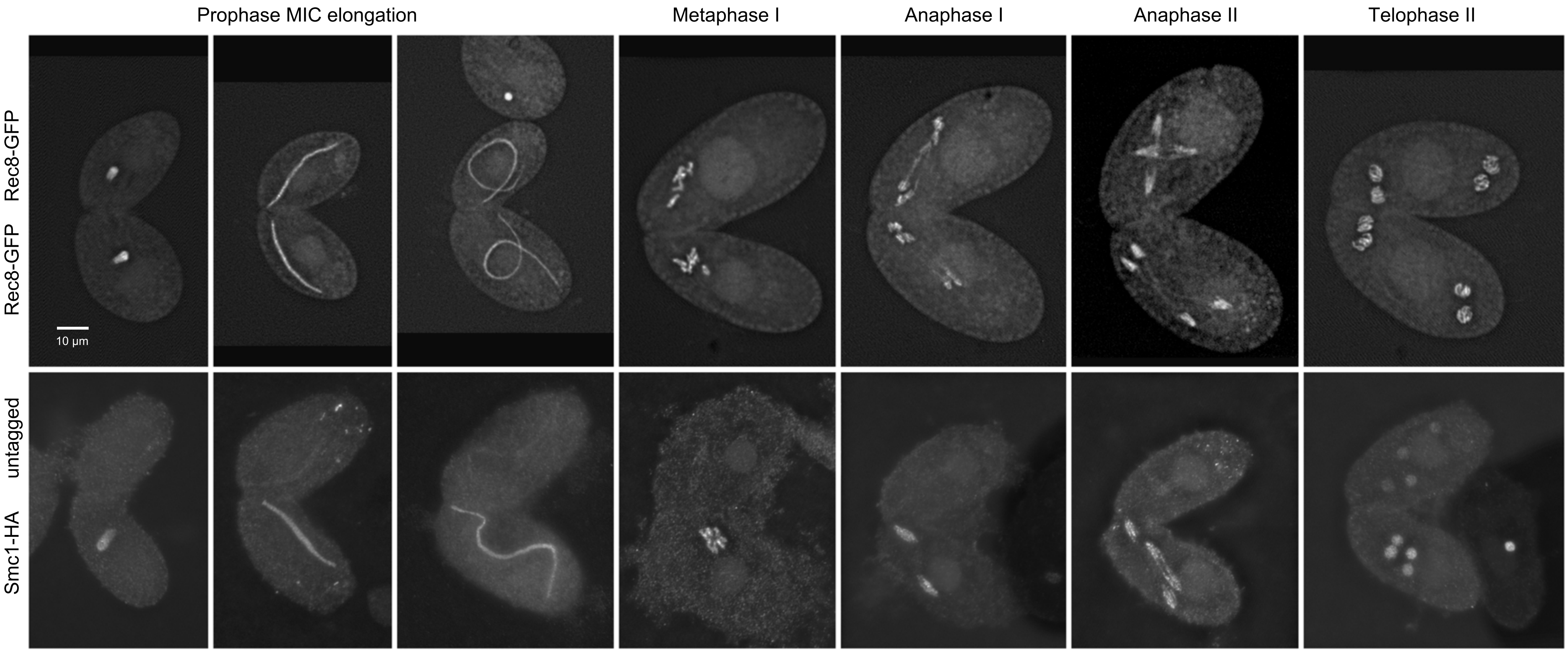
Prophase MIC elongation
Metaphase I
Anaphase I
Anaphase II
Telophase II
Rec8-GFP
Rec8-GFP
10 µm
untagged
Smc1-HA
