# Supplementary material for: A Single Cohesin Complex Performs Mitotic and Meiotic Functions in the Protist Tetrahymena
Source: PLoS Genet. 2013 Mar 28;9(3):e1003418. doi: 10.1371/journal.pgen.1003418 (PMC3610610; doi:10.1371/journal.pgen.1003418)
Supplement: Table S1 — Proteins identified by mass spectrometry analysis of Rec8-GFP immunoprecipitation. (PDF) [file pgen.1003418.s007.pdf]

Probability Legend

- over 95%
- 80% to 94%
- 50% to 79%
- 20% to 49%
- 0% to 19%

#	Visible?	Starred?	Bio View: Identified Proteins (1837)	Accession Number	Molecular Weight	Protein Grouping Ambiguity	01 Control	02 Sample
1	☑	☆	SMC family, C-terminal domain containing protein [Tetrahymena thermophila]	gi|118384729	151 kDa		3	69
2	☑	☆	Dynein heavy chain family protein [Tetrahymena thermophila]	gi|118378024	530 kDa	★	106	55
3	☑	☆	Dynein heavy chain family protein [Tetrahymena thermophila]	gi|118394992	534 kDa		100	45
4	☑	☆	SMC family, C-terminal domain containing protein [Tetrahymena thermophila]	gi|118359750	226 kDa	★	5	44
5	☑	☆	Hsp90 protein [Tetrahymena thermophila]	gi|118353019 (+1)	82 kDa	★	43	43
6	☑	☆	hypothetical protein TTHERM_00427560 [Tetrahymena thermophila]	gi|118361209	111 kDa		27	40
7	☑	☆	Piwi domain containing protein [Tetrahymena thermophila]	gi|118370188	90 kDa		29	39
8	☑	☆	Dynein heavy chain family protein [Tetrahymena thermophila]	gi|118401102	482 kDa	★	62	36
9	☑	☆	Hsp90 protein [Tetrahymena thermophila]	gi|118380332	92 kDa		34	34
10	☑	☆	Region in Clathrin and VPS family protein [Tetrahymena thermophila]	gi|118365589	203 kDa		57	33
11	☑	☆	hypothetical protein TTHERM_00833620 [Tetrahymena thermophila]	gi|118360876	297 kDa	★	42	33
12	☑	☆	AAA family ATPase, CDC48 subfamily protein [Tetrahymena thermophila]	gi|118347942	93 kDa	★	10	33
13	☑	☆	Elongation factor G, domain IV family protein [Tetrahymena thermophila]	gi|146185140	93 kDa	★	20	32
14	☑	☆	hypothetical protein TTHERM_00444610 [Tetrahymena thermophila]	gi|118380320	288 kDa			30
15	☑	☆	hypothetical protein TTHERM_00145010 [Tetrahymena thermophila]	gi|118355764	210 kDa		10	29
16	☑	☆	Tubulin beta chain, putative [Tetrahymena thermophila]	gi|118379681 (+2)	50 kDa	★	27	28
17	☑	☆	hypothetical protein TTHERM_00499420 [Tetrahymena thermophila]	gi|146181127	83 kDa		35	27
18	☑	☆	ribosomal protein L4/L1 family protein [Tetrahymena thermophila]	gi|146170339	45 kDa		27	27
19	☑	☆	dnaK protein [Tetrahymena thermophila]	gi|118358030	71 kDa	★	26	26
20	☑	☆	hypothetical protein TTHERM_01345750 [Tetrahymena thermophila]	gi|146184590	89 kDa		15	26
21	☑	☆	phosphoglycerate kinase family protein [Tetrahymena thermophila]	gi|118385141	46 kDa			25
22	☑	☆	WGR domain containing protein [Tetrahymena thermophila]	gi|118378276	299 kDa		58	24
23	☑	☆	DNA double-strand break repair rad50 ATPase, putative [Tetrahymena thermophila]	gi|146181867	51 kDa	★	22	24
24	☑	☆	V-type ATPase, A subunit family protein [Tetrahymena thermophila]	gi|146170652	69 kDa		18	24
25	☑	☆	TCP-1/cpn60 chaperonin family protein [Tetrahymena thermophila]	gi|146182577	62 kDa		17	24
26	☑	☆	alanyl-tRNA synthetase family protein [Tetrahymena thermophila]	gi|118374997	121 kDa		13	24
27	☑	☆	hypothetical protein TTHERM_00473320 [Tetrahymena thermophila]	gi|118381509	101 kDa		7	24
28	☑	☆	RNA polymerase Rpb1, domain 2 family protein [Tetrahymena thermophila]	gi|118348890	202 kDa		6	24
29	☑	☆	glycosyl transferase, group 1 family protein [Tetrahymena thermophila]	gi|146183813	214 kDa		6	24
30	☑	☆	calcium-translocating P-type ATPase, SERCA-type family protein [Tetrahymena thermophila]	gi|118396179	121 kDa		28	23
31	☑	☆	Leucine Rich Repeat family protein [Tetrahymena thermophila]	gi|118376294	70 kDa		12	23
32	☑	☆	Proteasome/cyclosome repeat family protein [Tetrahymena thermophila]	gi|118368838	99 kDa		27	22
33	☑	☆	SnoRNA binding domain containing protein [Tetrahymena thermophila]	gi|118363018	51 kDa	★	20	22
34	☑	☆	hypothetical protein TTHERM_00971830 [Tetrahymena thermophila]	gi|118385961	60 kDa		13	22
35	☑	☆	hypothetical protein TTHERM_00857910 [Tetrahymena thermophila]	gi|118377510	55 kDa		13	22
36	☑	☆	DNA-directed RNA polymerase, beta subunit family protein [Tetrahymena thermophila]	gi|118364886	140 kDa		12	22
37	☑	☆	Pyridine nucleotide-disulphide oxidoreductase family protein [Tetrahymena thermophila]	gi|118378644	70 kDa		6	22

Probability Legend

- over 95%
- 80% to 94%
- 50% to 79%
- 20% to 49%
- 0% to 19%

#	Visible?	Starred?	Bio View: Identified Proteins (1837)	Accession Number	Molecular Weight	Protein Grouping Ambiguity	01 Control	02 Sample
38	☑	☆	hypothetical protein TTHERM_00530680 [Tetrahymena thermophila]	gi|118400903	259 kDa		5	22
39	☑	☆	SnoRNA binding domain containing protein [Tetrahymena thermophila]	gi|118378385	58 kDa	★	21	21
40	☑	☆	hypothetical protein TTHERM_00550810 [Tetrahymena thermophila]	gi|118351795	121 kDa		11	21
41	☑	☆	enoyl-CoA hydratase/isomerase family protein [Tetrahymena thermophila]	gi|146161460	171 kDa		10	21
42	☑	☆	Glutamate/Leucine/Phenylalanine/Valine dehydrogenase family protein [Tetrahymena thermophila]	gi|118384751	68 kDa		4	21
43	☑	☆	ubiquitin-activating enzyme E1 family protein [Tetrahymena thermophila]	gi|118376746	720 kDa	★	38	20
44	☑	☆	tetrin C protein, putative [Tetrahymena thermophila]	gi|118345618	89 kDa		34	20
45	☑	☆	Viral A-type inclusion protein repeat containing protein [Tetrahymena thermophila]	gi|146165611	133 kDa		31	20
46	☑	☆	hypothetical protein TTHERM_00221120 [Tetrahymena thermophila]	gi|146179848	85 kDa		28	20
47	☑	☆	prolyl-tRNA synthetase family protein [Tetrahymena thermophila]	gi|118401186	80 kDa		24	20
48	☑	☆	hypothetical protein TTHERM_00426260 [Tetrahymena thermophila]	gi|146164672	70 kDa		22	20
49	☑	☆	hypothetical protein TTHERM_00625950 [Tetrahymena thermophila]	gi|118372568	45 kDa		21	20
50	☑	☆	Tubulin/FtsZ family, GTPase domain containing protein [Tetrahymena thermophila]	gi|118378497	50 kDa	★	18	20
51	☑	☆	hypothetical protein TTHERM_00471040 [Tetrahymena thermophila]	gi|118401347	126 kDa		35	19
52	☑	☆	hypothetical protein TTHERM_00194540 [Tetrahymena thermophila]	gi|118367995	145 kDa		27	19
53	☑	☆	hypothetical protein TTHERM_00086780 [Tetrahymena thermophila]	gi|118358970	70 kDa		22	19
54	☑	☆	ADP , ATP carrier protein 1, mitochondrial precursor [Tetrahymena thermophila]	gi|118363396	34 kDa		21	19
55	☑	☆	glyceraldehyde-3-phosphate dehydrogenase, type I family protein [Tetrahymena thermophila]	gi|118351857	37 kDa		20	19
56	☑	☆	Fibrillarin, putative [Tetrahymena thermophila]	gi|118373192	31 kDa		19	19
57	☑	☆	Chain W, Crystal Structure Of The Eukaryotic 40s Ribosomal Subunit In Complex With Initiation Factor 1. This File Contains The 40s Sub...	gi|319443378	30 kDa		18	19
58	☑	☆	acetyl-CoA acyltransferases family protein [Tetrahymena thermophila]	gi|118397550	41 kDa		18	19
59	☑	☆	conserved hypothetical protein [Tetrahymena thermophila]	gi|146175330	56 kDa		17	19
60	☑	☆	acyl carrier protein reductase [Tetrahymena thermophila]	gi|118376486	49 kDa		16	19
61	☑	☆	HMG box family protein [Tetrahymena thermophila]	gi|229596407	76 kDa		15	19
62	☑	☆	TCP-1/cpn60 chaperonin family protein [Tetrahymena thermophila]	gi|146175771	59 kDa		8	19
63	☑	☆	threonyl-tRNA synthetase family protein [Tetrahymena thermophila]	gi|146169598	101 kDa		6	19
64	☑	☆	Zinc knuckle family protein [Tetrahymena thermophila]	gi|146182859 (+1)	200 kDa		51	18
65	☑	☆	Protein kinase domain containing protein [Tetrahymena thermophila]	gi|118401762	616 kDa		23	18
66	☑	☆	ATP synthase F1, alpha subunit family protein [Tetrahymena thermophila]	gi|146184059	60 kDa		19	18
67	☑	☆	ATP synthase beta chain, mitochondrial precursor, putative [Tetrahymena thermophila]	gi|146185860	53 kDa		18	18
68	☑	☆	hypothetical protein TTHERM_00721790 [Tetrahymena thermophila]	gi|229594797	53 kDa		17	18
69	☑	☆	hypothetical protein TTHERM_00437360 [Tetrahymena thermophila]	gi|118369054	87 kDa	★	16	18
70	☑	☆	conserved hypothetical protein [Tetrahymena thermophila]	gi|118352310	44 kDa		15	18
71	☑	☆	TCP-1/cpn60 chaperonin family protein [Tetrahymena thermophila]	gi|118376776	59 kDa		12	18
72	☑	☆	EF hand family protein [Tetrahymena thermophila]	gi|146185558	80 kDa		22	17
73	☑	☆	conserved hypothetical protein [Tetrahymena thermophila]	gi|118359722	86 kDa		20	17
74	☑	☆	Viral A-type inclusion protein repeat containing protein [Tetrahymena thermophila]	gi|118379374	311 kDa		20	17

Probability Legend

- over 95%
- 80% to 94%
- 50% to 79%
- 20% to 49%
- 0% to 19%

#	Visible?	Starred?	Bio View: Identified Proteins (1837)	Accession Number	Molecular Weight	Protein Grouping Ambiguity	01 Control	02 Sample
75	☑	☆	hypothetical protein TTHERM_00361490 [Tetrahymena thermophila]	gi|146161516	58 kDa	★	17	17
76	☑	☆	D-hydantoinase family protein [Tetrahymena thermophila]	gi|118361640	166 kDa		17	17
77	☑	☆	NADH-ubiquinone oxidoreductase 75 kDa subunit, mitochondrial precursor, putative [Tetrahymena thermophila]	gi|229595475	80 kDa		13	17
78	☑	☆	V-type ATPase, B subunit family protein [Tetrahymena thermophila]	gi|118400751	55 kDa		11	17
79	☑	☆	hypothetical protein TTHERM_01207720 [Tetrahymena thermophila]	gi|118377477	81 kDa		11	17
80	☑	☆	dnaK protein [Tetrahymena thermophila]	gi|118396970	71 kDa	★	10	17
81	☑	☆	Ubiquitin carboxyl-terminal hydrolase family protein [Tetrahymena thermophila]	gi|118362860	391 kDa		9	17
82	☑	☆	Adenylate kinase family protein [Tetrahymena thermophila]	gi|118376438	118 kDa	★	7	17
83	☑	☆	glutamyl-tRNA synthetase [Tetrahymena thermophila]	gi|118402007	133 kDa			17
84	☑	☆	RNA polymerase Rpb1, domain 2 family protein [Tetrahymena thermophila]	gi|118363248	198 kDa			17
85	☑	☆	Peptidase family M1 containing protein [Tetrahymena thermophila]	gi|118379875	136 kDa			17
86	☑	☆	SMC family, C-terminal domain containing protein [Tetrahymena thermophila]	gi|229594500	159 kDa		30	16
87	☑	☆	tetrin A protein, putative [Tetrahymena thermophila]	gi|118349664	99 kDa		21	16
88	☑	☆	hypothetical protein TTHERM_00550700 [Tetrahymena thermophila]	gi|146162278	127 kDa		20	16
89	☑	☆	methionyl-tRNA synthetase [Tetrahymena thermophila]	gi|118386032	96 kDa		19	16
90	☑	☆	tetrin B protein [Tetrahymena thermophila]	gi|10045220 (+1)	87 kDa		18	16
91	☑	☆	translation elongation factor EF-1, subunit alpha [Tetrahymena thermophila]	gi|118399778	48 kDa		17	16
92	☑	☆	RecName: Full=60S ribosomal protein L7a	gi|353678103	29 kDa		15	16
93	☑	☆	Peptidase M16 inactive domain containing protein [Tetrahymena thermophila]	gi|229594165	53 kDa		14	16
94	☑	☆	Cullin family protein [Tetrahymena thermophila]	gi|118362587	104 kDa		14	16
95	☑	☆	hypothetical protein TTHERM_00971710 [Tetrahymena thermophila]	gi|118385937	83 kDa		4	16
96	☑	☆	26S proteasome subunit P45 family protein [Tetrahymena thermophila]	gi|146171839	48 kDa	★	18	15
97	☑	☆	peptidase M16 inactive domain containing protein [Tetrahymena thermophila]	gi|146181190	58 kDa		18	15
98	☑	☆	DnaJ domain containing protein [Tetrahymena thermophila]	gi|229596294	49 kDa		17	15
99	☑	☆	KH domain containing protein [Tetrahymena thermophila]	gi|229594319	28 kDa		14	15
100	☑	☆	Phosphofructokinase family protein [Tetrahymena thermophila]	gi|118350827	60 kDa		14	15
101	☑	☆	TCP-1/cpn60 chaperonin family protein [Tetrahymena thermophila]	gi|146161291	59 kDa		13	15
102	☑	☆	succinyl-CoA synthetase, beta subunit family protein [Tetrahymena thermophila]	gi|146181817	47 kDa		13	15
103	☑	☆	Growth-arrest-specific protein 8 [Tetrahymena thermophila]	gi|146185685	56 kDa		10	15
104	☑	☆	hypothetical protein TTHERM_00052510 [Tetrahymena thermophila]	gi|118362900	43 kDa		4	15
105	☑	☆	TPR Domain containing protein [Tetrahymena thermophila]	gi|118374703	101 kDa			15
106	☑	☆	Ubiquitin carboxyl-terminal hydrolase family protein [Tetrahymena thermophila]	gi|118401690	121 kDa			15
107	☑	☆	hypothetical protein TTHERM_00474830 [Tetrahymena thermophila]	gi|118381609	87 kDa		35	14
108	☑	☆	SMC family, C-terminal domain containing protein [Tetrahymena thermophila]	gi|118350688	146 kDa		26	14
109	☑	☆	hypothetical protein TTHERM_00688340 [Tetrahymena thermophila]	gi|118387667	39 kDa		18	14
110	☑	☆	TCP-1/cpn60 chaperonin family protein [Tetrahymena thermophila]	gi|146163136	60 kDa		17	14
111	☑	☆	hypothetical protein TTHERM_00777250 [Tetrahymena thermophila]	gi|229594893	35 kDa	★	16	14

Probability Legend

- over 95%
- 80% to 94%
- 50% to 79%
- 20% to 49%
- 0% to 19%

#	Visible?	Starred?	Bio View: Identified Proteins (1837)	Accession Number	Molecular Weight	Protein Grouping Ambiguity	01 Control	02 Sample
112	☑	☆	HEAT repeat family protein [Tetrahymena thermophila]	gi|146182494	66 kDa		16	14
113	☑	☆	hypothetical protein TTHERM_00586620 [Tetrahymena thermophila]	gi|118373347	92 kDa	★	16	14
114	☑	☆	EF hand family protein [Tetrahymena thermophila]	gi|118345736	24 kDa		14	14
115	☑	☆	hypothetical protein TTHERM_00131180 [Tetrahymena thermophila]	gi|229595140	97 kDa		14	14
116	☑	☆	Leucine Rich Repeat family protein [Tetrahymena thermophila]	gi|118368860	58 kDa		13	14
117	☑	☆	Ribosomal S3Ae family protein [Tetrahymena thermophila]	gi|146165293	30 kDa		12	14
118	☑	☆	hypothetical protein TTHERM_00382330 [Tetrahymena thermophila]	gi|146165634	39 kDa		12	14
119	☑	☆	hypothetical protein TTHERM_01528510 [Tetrahymena thermophila]	gi|229594147	32 kDa		11	14
120	☑	☆	14-3-3 protein [Tetrahymena thermophila]	gi|118374567	28 kDa	★	11	14
121	☑	☆	Calpain family cysteine protease containing protein [Tetrahymena thermophila]	gi|118399213	95 kDa		11	14
122	☑	☆	TCP-1/cpn60 chaperonin family protein [Tetrahymena thermophila]	gi|146161289	60 kDa	★	10	14
123	☑	☆	hypothetical protein TTHERM_00499440 [Tetrahymena thermophila]	gi|118378052	41 kDa		10	14
124	☑	☆	Protein kinase domain containing protein [Tetrahymena thermophila]	gi|118353531	56 kDa		6	14
125	☑	☆	hypothetical protein TTHERM_00773160 [Tetrahymena thermophila]	gi|118398482	86 kDa		6	14
126	☑	☆	hypothetical protein TTHERM_00813040 [Tetrahymena thermophila]	gi|118350706	130 kDa		4	14
127	☑	☆	hypothetical protein TTHERM_00443100 [Tetrahymena thermophila]	gi|146186230	86 kDa		3	14
128	☑	☆	hypothetical protein TTHERM_00245660 [Tetrahymena thermophila]	gi|118381268	71 kDa			14
129	☑	☆	hypothetical protein TTHERM_00926960 [Tetrahymena thermophila]	gi|118397546	70 kDa		22	13
130	☑	☆	actin, macronuclear [Tetrahymena thermophila]	gi|118366913	42 kDa	★	20	13
131	☑	☆	TPR Domain containing protein [Tetrahymena thermophila]	gi|118365491	391 kDa		19	13
132	☑	☆	conserved hypothetical protein [Tetrahymena thermophila]	gi|118373588	65 kDa		19	13
133	☑	☆	hypothetical protein TTHERM_00527180 [Tetrahymena thermophila]	gi|229593556	45 kDa		17	13
134	☑	☆	ribosomal protein S5 containing protein [Tetrahymena thermophila]	gi|229595466	34 kDa		16	13
135	☑	☆	hypothetical protein TTHERM_00149798 [Tetrahymena thermophila]	gi|146180853	33 kDa		15	13
136	☑	☆	hypothetical protein TTHERM_00378620 [Tetrahymena thermophila]	gi|146165583	69 kDa		15	13
137	☑	☆	hypothetical protein TTHERM_00312820 [Tetrahymena thermophila]	gi|118349173	35 kDa		15	13
138	☑	☆	hypothetical protein TTHERM_00117590 [Tetrahymena thermophila]	gi|146163103	35 kDa		14	13
139	☑	☆	hypothetical protein TTHERM_00522980 [Tetrahymena thermophila]	gi|118362498	186 kDa		13	13
140	☑	☆	Ribosomal protein L13e containing protein [Tetrahymena thermophila]	gi|118379398	23 kDa		13	13
141	☑	☆	L1P family of ribosomal proteins containing protein [Tetrahymena thermophila]	gi|146162404	24 kDa		13	13
142	☑	☆	hypothetical protein TTHERM_00136440 [Tetrahymena thermophila]	gi|118373062	78 kDa		13	13
143	☑	☆	Coatomer WD associated domain containing protein [Tetrahymena thermophila]	gi|146170021	103 kDa		13	13
144	☑	☆	ribosomal protein L3 containing protein [Tetrahymena thermophila]	gi|118389862	44 kDa	★	12	13
145	☑	☆	Regulator of chromosome condensation [Tetrahymena thermophila]	gi|146162003	51 kDa		12	13
146	☑	☆	TCP-1/cpn60 chaperonin family protein [Tetrahymena thermophila]	gi|118386505	59 kDa		10	13
147	☑	☆	Na,H/K antiporter P-type ATPase, alpha subunit family protein [Tetrahymena thermophila]	gi|118402051	133 kDa	★	10	13
148	☑	☆	isoleucyl-tRNA synthetase family protein [Tetrahymena thermophila]	gi|118380025 (+1)	128 kDa		7	13

Probability Legend: over 95% | 80% to 94% | 50% to 79% | 20% to 49% | 0% to 19%

#	Visible?	Starred?	Bio View: Identified Proteins (1837)	Accession Number	Molecular Weight	Protein Grouping Ambiguity	01 Control	02 Sample
149	☑	☆	hypothetical protein TTHERM_00160960 [Tetrahymena thermophila]	gi|118353111	42 kDa		7	13
150	☑	☆	hypothetical protein TTHERM_00582070 [Tetrahymena thermophila]	gi|118371498	58 kDa		5	13
151	☑	☆	Type III restriction enzyme, res subunit family protein [Tetrahymena thermophila]	gi|118382948	264 kDa		4	13
152	☑	☆	glutaminyl-tRNA synthetase family protein [Tetrahymena thermophila]	gi|146161699	89 kDa		3	13
153	☑	☆	U-box domain containing protein [Tetrahymena thermophila]	gi|229595209	130 kDa			13
154	☑	☆	phenylalanyl-tRNA synthetase, alpha subunit family protein [Tetrahymena thermophila]	gi|118372908	59 kDa			13
155	☑	☆	Vacuolar ATP synthase subunit C [Tetrahymena thermophila]	gi|146163772	47 kDa			13
156	☑	☆	Dynein heavy chain family protein [Tetrahymena thermophila]	gi|118387693	509 kDa	★	37	12
157	☑	☆	Zinc knuckle family protein [Tetrahymena thermophila]	gi|146184871	178 kDa		30	12
158	☑	☆	Kinesin motor domain containing protein [Tetrahymena thermophila]	gi|118400729	150 kDa		29	12
159	☑	☆	TIP49 C-terminus family protein [Tetrahymena thermophila]	gi|146170108	50 kDa		23	12
160	☑	☆	TATA box-binding protein [Tetrahymena thermophila]	gi|118363406	52 kDa		19	12
161	☑	☆	hypothetical protein TTHERM_00128280 [Tetrahymena thermophila]	gi|118366181	48 kDa		18	12
162	☑	☆	hypothetical protein TTHERM_00051730 [Tetrahymena thermophila]	gi|118363064	87 kDa		18	12
163	☑	☆	hypothetical protein TTHERM_01002870 [Tetrahymena thermophila]	gi|229594034	48 kDa	★	16	12
164	☑	☆	Eukaryotic aspartyl protease family protein [Tetrahymena thermophila]	gi|229595292	57 kDa		16	12
165	☑	☆	hypothetical protein TTHERM_00688560 [Tetrahymena thermophila]	gi|118387711	29 kDa		16	12
166	☑	☆	inorganic pyrophosphatase [Tetrahymena thermophila]	gi|118374727	79 kDa	★	15	12
167	☑	☆	hypothetical protein TTHERM_00059330 [Tetrahymena thermophila]	gi|118348380	42 kDa		15	12
168	☑	☆	dnak protein BiP [Tetrahymena thermophila]	gi|118350929	72 kDa	★	15	12
169	☑	☆	hypothetical protein TTHERM_01002860 [Tetrahymena thermophila]	gi|229594032	48 kDa	★	14	12
170	☑	☆	hypothetical protein TTHERM_00136120 [Tetrahymena thermophila]	gi|146175708	22 kDa		13	12
171	☑	☆	60s Acidic ribosomal protein [Tetrahymena thermophila]	gi|146185707	35 kDa		13	12
172	☑	☆	cytochrome c oxidase subunit 2 [Tetrahymena thermophila]	gi|15027663	72 kDa		13	12
173	☑	☆	Thioredoxin family protein [Tetrahymena thermophila]	gi|118345734	56 kDa		12	12
174	☑	☆	acetyl-CoA acyltransferases family protein [Tetrahymena thermophila]	gi|146180950	44 kDa		12	12
175	☑	☆	hypothetical protein TTHERM_01513260 [Tetrahymena thermophila]	gi|118390336	49 kDa		12	12
176	☑	☆	Ras family protein [Tetrahymena thermophila]	gi|146162031	26 kDa	★	10	12
177	☑	☆	Ribosomal S17 family protein [Tetrahymena thermophila]	gi|118384398	15 kDa		10	12
178	☑	☆	hypothetical protein TTHERM_00046440 [Tetrahymena thermophila]	gi|118362736	62 kDa		10	12
179	☑	☆	catalase family protein [Tetrahymena thermophila]	gi|146183614 (+1)	56 kDa		10	12
180	☑	☆	Citrate synthase family protein [Tetrahymena thermophila]	gi|118364649	68 kDa	★	10	12
181	☑	☆	eukaryotic translation initiation factor 3 subunit 8 [Tetrahymena thermophila]	gi|118364431	117 kDa		9	12
182	☑	☆	polyadenylate-binding protein 2 [Tetrahymena thermophila]	gi|118359856	70 kDa		9	12
183	☑	☆	leucyl-tRNA synthetase family protein [Tetrahymena thermophila]	gi|118386473	120 kDa		7	12
184	☑	☆	enolase family protein [Tetrahymena thermophila]	gi|118362946	50 kDa		4	12
185	☑	☆	hypothetical protein TTHERM_00424720 [Tetrahymena thermophila]	gi|118361037	84 kDa		3	12

Probability Legend

- over 95%
- 80% to 94%
- 50% to 79%
- 20% to 49%
- 0% to 19%

#	Visible?	Starred?	Bio View: Identified Proteins (1837)	Accession Number	Molecular Weight	Protein Grouping Ambiguity	01 Control	02 Sample
186	☑	☆	PH domain containing protein [Tetrahymena thermophila]	gi|118355218 (+1)	444 kDa		50	11
187	☑	☆	Kinesin motor domain containing protein [Tetrahymena thermophila]	gi|118356524	153 kDa		31	11
188	☑	☆	BNR/Asp-box repeat family protein [Tetrahymena thermophila]	gi|146161811	99 kDa		22	11
189	☑	☆	hypothetical protein TTHERM_00204140 [Tetrahymena thermophila]	gi|118347246	51 kDa		22	11
190	☑	☆	hypothetical protein TTHERM_01091280 [Tetrahymena thermophila]	gi|118384580	56 kDa		18	11
191	☑	☆	SPFH domain / Band 7 family protein [Tetrahymena thermophila]	gi|118357195	43 kDa	★	17	11
192	☑	☆	hypothetical protein TTHERM_00373830 [Tetrahymena thermophila]	gi|118352680	54 kDa	★	16	11
193	☑	☆	hypothetical protein TTHERM_00780850 [Tetrahymena thermophila]	gi|229593882	75 kDa		16	11
194	☑	☆	ATP-dependent metalloprotease FtsH family protein [Tetrahymena thermophila]	gi|118347625	101 kDa	★	14	11
195	☑	☆	TCP-1/cpn60 chaperonin family protein [Tetrahymena thermophila]	gi|146184205	62 kDa		14	11
196	☑	☆	PCI domain containing protein [Tetrahymena thermophila]	gi|146181768	57 kDa		13	11
197	☑	☆	granule lattice protein 4 precursor, putative [Tetrahymena thermophila]	gi|229594682	43 kDa		12	11
198	☑	☆	hypothetical protein TTHERM_00557840 [Tetrahymena thermophila]	gi|146181221	36 kDa		12	11
199	☑	☆	hypothetical protein TTHERM_00051840 [Tetrahymena thermophila]	gi|118363272	40 kDa		11	11
200	☑	☆	hypothetical protein TTHERM_00558360 [Tetrahymena thermophila]	gi|146181242	25 kDa		11	11
201	☑	☆	lipid A-disaccharide synthase [Tetrahymena thermophila]	gi|118397639	59 kDa		10	11
202	☑	☆	hypothetical protein TTHERM_00079230 [Tetrahymena thermophila]	gi|118365086	77 kDa		10	11
203	☑	☆	hypothetical protein TTHERM_01220370 [Tetrahymena thermophila]	gi|118385250	41 kDa		10	11
204	☑	☆	calcium-translocating P-type ATPase, PMCA-type family protein [Tetrahymena thermophila]	gi|118365447	124 kDa	★	10	11
205	☑	☆	ribose-phosphate pyrophosphokinase family protein [Tetrahymena thermophila]	gi|118380753	44 kDa		9	11
206	☑	☆	Chain I, Crystal Structure Of The Eukaryotic 40s Ribosomal Subunit In Complex With Initiation Factor 1. This File Contains The 40s Subu...	gi|319443364	16 kDa		8	11
207	☑	☆	40S ribosomal protein S8 [Tetrahymena thermophila]	gi|118395488	24 kDa		8	11
208	☑	☆	hypothetical protein TTHERM_00561490 [Tetrahymena thermophila]	gi|118353876	71 kDa	★	8	11
209	☑	☆	hypothetical protein TTHERM_00218840 [Tetrahymena thermophila]	gi|229594991	20 kDa		8	11
210	☑	☆	PX domain containing protein [Tetrahymena thermophila]	gi|118378200	48 kDa		8	11
211	☑	☆	Proline dehydrogenase family protein [Tetrahymena thermophila]	gi|118359866	66 kDa		7	11
212	☑	☆	hypothetical protein TTHERM_00647339 [Tetrahymena thermophila]	gi|146183955	127 kDa		6	11
213	☑	☆	Calpain family cysteine protease containing protein [Tetrahymena thermophila]	gi|118368219	79 kDa		5	11
214	☑	☆	Elongation factor Tu, mitochondrial precursor, putative [Tetrahymena thermophila]	gi|118377064	41 kDa	★	5	11
215	☑	☆	hypothetical protein TTHERM_01289160 [Tetrahymena thermophila]	gi|146184650	46 kDa		4	11
216	☑	☆	ribosomal protein S9 containing protein [Tetrahymena thermophila]	gi|146183499	90 kDa		4	11
217	☑	☆	alcohol dehydrogenase family protein [Tetrahymena thermophila]	gi|118359928	76 kDa			11
218	☑	☆	Protein kinase domain containing protein [Tetrahymena thermophila]	gi|118358108	96 kDa			11
219	☑	☆	Dynein heavy chain family protein [Tetrahymena thermophila]	gi|118374012	562 kDa		42	10
220	☑	☆	Dynein heavy chain family protein [Tetrahymena thermophila]	gi|118363224	529 kDa		38	10
221	☑	☆	Viral A-type inclusion protein repeat containing protein [Tetrahymena thermophila]	gi|118400078	145 kDa		29	10
222	☑	☆	AMP-binding enzyme family protein [Tetrahymena thermophila]	gi|118364601	75 kDa	★	19	10

Probability Legend

- over 95%
- 80% to 94%
- 50% to 79%
- 20% to 49%
- 0% to 19%

#	Visible?	Starred?	Bio View: Identified Proteins (1837)	Accession Number	Molecular Weight	Protein Grouping Ambiguity	01 Control	02 Sample
223	☑	☆	E1-E2 ATPase family protein [Tetrahymena thermophila]	gi|118376028 (+1)	128 kDa		16	10
224	☑	☆	hypothetical protein TTHERM_00446190 [Tetrahymena thermophila]	gi|118380434	38 kDa		15	10
225	☑	☆	26S proteasome subunit P45 family protein [Tetrahymena thermophila]	gi|118373046	49 kDa	★	15	10
226	☑	☆	hypothetical protein TTHERM_00685980 [Tetrahymena thermophila]	gi|118384094	38 kDa		14	10
227	☑	☆	hypothetical protein TTHERM_00941400 [Tetrahymena thermophila]	gi|146183721	31 kDa		13	10
228	☑	☆	hypothetical protein TTHERM_00070850 [Tetrahymena thermophila]	gi|146161569	36 kDa		13	10
229	☑	☆	cytidyltransferase-related domain containing protein [Tetrahymena thermophila]	gi|118356113	54 kDa	★	12	10
230	☑	☆	D-isomer specific 2-hydroxyacid dehydrogenase, NAD binding domain containing protein [Tetrahymena thermophila]	gi|146185050	41 kDa		11	10
231	☑	☆	hypothetical protein TTHERM_00997710 [Tetrahymena thermophila]	gi|118372027	15 kDa	★	10	10
232	☑	☆	ribosomal protein S4 containing protein [Tetrahymena thermophila]	gi|146163443	21 kDa		10	10
233	☑	☆	hypothetical protein TTHERM_00399360 [Tetrahymena thermophila]	gi|146164764	33 kDa	★	10	10
234	☑	☆	RecName: Full=60S ribosomal protein L7	gi|353678104	28 kDa		9	10
235	☑	☆	DNA-directed rna polymerase II subunit [Tetrahymena thermophila]	gi|146169387	36 kDa		9	10
236	☑	☆	Ribosomal protein L11, RNA binding domain containing protein [Tetrahymena thermophila]	gi|146161229	18 kDa		8	10
237	☑	☆	Mitochondrial carrier protein [Tetrahymena thermophila]	gi|118347912	34 kDa		7	10
238	☑	☆	CTP synthase family protein [Tetrahymena thermophila]	gi|118382175	69 kDa		7	10
239	☑	☆	tryptophanyl-tRNA synthetase family protein [Tetrahymena thermophila]	gi|118378144	48 kDa		7	10
240	☑	☆	Pyridine nucleotide-disulphide oxidoreductase family protein [Tetrahymena thermophila]	gi|118382672	70 kDa		6	10
241	☑	☆	3-hydroxyacyl-CoA dehydrogenase, NAD binding domain [Tetrahymena thermophila]	gi|118373680	33 kDa		4	10
242	☑	☆	Adaptor complexes medium subunit family protein [Tetrahymena thermophila]	gi|118395754	52 kDa		4	10
243	☑	☆	Acyl-CoA dehydrogenase, C-terminal domain containing protein [Tetrahymena thermophila]	gi|146161808	47 kDa		3	10
244	☑	☆	Glutamate/Leucine/Phenylalanine/Valine dehydrogenase family protein [Tetrahymena thermophila]	gi|118377975	56 kDa		3	10
245	☑	☆	ribosomal protein L3 containing protein [Tetrahymena thermophila]	gi|229594830	50 kDa		3	10
246	☑	☆	RecName: Full=Citrate synthase, mitochondrial; AltName: Full=14 nm filament-forming protein; AltName: Full=49 kDa protein; Flags: Pr...	gi|116475	53 kDa		2	10
247	☑	☆	hypothetical protein TTHERM_00194530 [Tetrahymena thermophila]	gi|118367993	86 kDa		2	10
248	☑	☆	citrate synthase I family protein [Tetrahymena thermophila]	gi|118363330 (+1)	55 kDa	★	2	10
249	☑	☆	citrate synthase [Tetrahymena thermophila]	gi|118376514	55 kDa			10
250	☑	☆	Vacuolar ATP synthase subunit H [Tetrahymena thermophila]	gi|146162669	53 kDa	★		10
251	☑	☆	hypothetical protein TTHERM_00621160 [Tetrahymena thermophila]	gi|118369474	78 kDa			10
252	☑	☆	Dynein heavy chain family protein [Tetrahymena thermophila]	gi|118356293	477 kDa	★	34	9
253	☑	☆	Pumilio-family RNA binding repeat containing protein [Tetrahymena thermophila]	gi|229594997	103 kDa		25	9
254	☑	☆	mRNA capping enzyme, large subunit family protein [Tetrahymena thermophila]	gi|118357970	80 kDa	★	19	9
255	☑	☆	Protein kinase domain containing protein [Tetrahymena thermophila]	gi|146168000	70 kDa	★	18	9
256	☑	☆	succinate dehydrogenase, flavoprotein subunit containing protein [Tetrahymena thermophila]	gi|146165264	70 kDa		15	9
257	☑	☆	hypothetical protein TTHERM_00777270 [Tetrahymena thermophila]	gi|118376159	36 kDa		15	9
258	☑	☆	AMP-binding enzyme family protein [Tetrahymena thermophila]	gi|146170344	77 kDa		15	9
259	☑	☆	Adenylate kinase family protein [Tetrahymena thermophila]	gi|146164901	50 kDa		14	9

Probability Legend: over 95% | 80% to 94% | 50% to 79% | 20% to 49% | 0% to 19%

#	Visible?	Starred?	Bio View: Identified Proteins (1837)	Accession Number	Molecular Weight	Protein Grouping Ambiguity	01 Control	02 Sample
260	☑	☆	hypothetical protein TTHERM_00473020 [Tetrahymena thermophila]	gi|229594355	48 kDa		12	9
261	☑	☆	kinase domain containing protein [Tetrahymena thermophila]	gi|229594519	153 kDa		11	9
262	☑	☆	hypothetical protein TTHERM_00571650 [Tetrahymena thermophila]	gi|118389158	36 kDa		11	9
263	☑	☆	vacuolar ATP synthase [Tetrahymena thermophila]	gi|146163407	44 kDa		11	9
264	☑	☆	DEAD/DEAH box helicase family protein [Tetrahymena thermophila]	gi|146182736	55 kDa		11	9
265	☑	☆	hypothetical protein TTHERM_00127260 [Tetrahymena thermophila]	gi|118366175	67 kDa		10	9
266	☑	☆	hypothetical protein TTHERM_00046550 [Tetrahymena thermophila]	gi|118362940	55 kDa		10	9
267	☑	☆	ribosomal protein S7 containing protein [Tetrahymena thermophila]	gi|118353446	22 kDa		9	9
268	☑	☆	hypothetical protein TTHERM_00474920 [Tetrahymena thermophila]	gi|118381627	51 kDa		9	9
269	☑	☆	ribosomal protein S13p/S18e containing protein [Tetrahymena thermophila]	gi|118372788	18 kDa		8	9
270	☑	☆	Ribosomal protein S24e containing protein [Tetrahymena thermophila]	gi|146182694	17 kDa		8	9
271	☑	☆	hypothetical protein TTHERM_00481300 [Tetrahymena thermophila]	gi|118368469	33 kDa	★	8	9
272	☑	☆	hypothetical protein TTHERM_00641250 [Tetrahymena thermophila]	gi|118364240	22 kDa		8	9
273	☑	☆	ATP synthase F1, gamma subunit family protein [Tetrahymena thermophila]	gi|118355000	33 kDa		8	9
274	☑	☆	Elongation factor G, domain IV family protein [Tetrahymena thermophila]	gi|118365220	112 kDa		8	9
275	☑	☆	hypothetical protein TTHERM_00486310 [Tetrahymena thermophila]	gi|229594811	28 kDa		7	9
276	☑	☆	hypothetical protein TTHERM_00433830 [Tetrahymena thermophila]	gi|146163428	20 kDa		7	9
277	☑	☆	Mitochondrial carrier protein [Tetrahymena thermophila]	gi|146183332	33 kDa		7	9
278	☑	☆	hypothetical protein TTHERM_00302110 [Tetrahymena thermophila]	gi|118382958	29 kDa		7	9
279	☑	☆	Ribosomal protein L7Ae containing protein [Tetrahymena thermophila]	gi|118353131	14 kDa		6	9
280	☑	☆	DEAD/DEAH box helicase family protein [Tetrahymena thermophila]	gi|118374174	54 kDa	★	6	9
281	☑	☆	hypothetical protein TTHERM_00013070 [Tetrahymena thermophila]	gi|118350012	89 kDa		6	9
282	☑	☆	asparaginyl-tRNA synthetase family protein [Tetrahymena thermophila]	gi|118399659	70 kDa		5	9
283	☑	☆	Protein kinase domain containing protein [Tetrahymena thermophila]	gi|146185241	60 kDa		5	9
284	☑	☆	DnaJ domain containing protein [Tetrahymena thermophila]	gi|118380591	59 kDa		5	9
285	☑	☆	FAD binding domain containing protein [Tetrahymena thermophila]	gi|118386939	88 kDa		5	9
286	☑	☆	succinyl-CoA synthetase, alpha subunit family protein [Tetrahymena thermophila]	gi|229596684	33 kDa		5	9
287	☑	☆	hypothetical protein TTHERM_01345770 [Tetrahymena thermophila]	gi|118394661	75 kDa		4	9
288	☑	☆	aldehyde dehydrogenase (NAD) family protein [Tetrahymena thermophila]	gi|146181428	55 kDa		3	9
289	☑	☆	hypothetical protein TTHERM_00214830 [Tetrahymena thermophila]	gi|118347404	157 kDa		2	9
290	☑	☆	Tudor domain containing protein [Tetrahymena thermophila]	gi|118400319	66 kDa			9
291	☑	☆	arginyl-tRNA synthetase family protein [Tetrahymena thermophila]	gi|118381734	71 kDa			9
292	☑	☆	hypothetical protein TTHERM_00420290 [Tetrahymena thermophila]	gi|118401929	103 kDa			9
293	☑	☆	dihydrolipoamide dehydrogenase family protein [Tetrahymena thermophila]	gi|146165187	52 kDa			9
294	☑	☆	polyA polymerase family protein [Tetrahymena thermophila]	gi|118378166	60 kDa			9
295	☑	☆	Malic enzyme, NAD binding domain containing protein [Tetrahymena thermophila]	gi|118389106	80 kDa			9
296	☑	☆	histidyl-tRNA synthetase family protein [Tetrahymena thermophila]	gi|118399178	105 kDa			9

Probability Legend

- over 95%
- 80% to 94%
- 50% to 79%
- 20% to 49%
- 0% to 19%

#	Visible?	Starred?	Bio View: Identified Proteins (1837)	Accession Number	Molecular Weight	Protein Grouping Ambiguity	01 Control	02 Sample
297	☑	☆	NAD-dependent glycerol-3-phosphate dehydrogenase C-terminus family protein [Tetrahymena thermophila]	gi|118400807	108 kDa			9
298	☑	☆	hypothetical protein TTHERM_00137780 [Tetrahymena thermophila]	gi|118373132	233 kDa			9
299	☑	☆	rRNA pseudouridine synthase, putative family protein [Tetrahymena thermophila]	gi|118357710	48 kDa	★	17	8
300	☑	☆	hypothetical protein TTHERM_00588900 [Tetrahymena thermophila]	gi|146176274	32 kDa		15	8
301	☑	☆	hypothetical protein TTHERM_00475310 [Tetrahymena thermophila]	gi|118381705	61 kDa		15	8
302	☑	☆	Polyprenyl synthetase family protein [Tetrahymena thermophila]	gi|146166126	48 kDa		15	8
303	☑	☆	Na,H/K antiporter P-type ATPase, alpha subunit family protein [Tetrahymena thermophila]	gi|118363110	153 kDa	★	15	8
304	☑	☆	hypothetical protein TTHERM_00469070 [Tetrahymena thermophila]	gi|118383898	138 kDa		15	8
305	☑	☆	hypothetical protein TTHERM_00188980 [Tetrahymena thermophila]	gi|118366717	30 kDa		13	8
306	☑	☆	hypothetical protein TTHERM_00434000 [Tetrahymena thermophila]	gi|146163439	56 kDa		13	8
307	☑	☆	TPR Domain containing protein [Tetrahymena thermophila]	gi|118384921	88 kDa		12	8
308	☑	☆	PCI domain containing protein [Tetrahymena thermophila]	gi|146165608	50 kDa		12	8
309	☑	☆	hypothetical protein TTHERM_00543590 [Tetrahymena thermophila]	gi|118345568	82 kDa		11	8
310	☑	☆	hypothetical protein TTHERM_00537380 [Tetrahymena thermophila]	gi|229594449	44 kDa		11	8
311	☑	☆	Sec23/Sec24 trunk domain containing protein [Tetrahymena thermophila]	gi|229593697	63 kDa		11	8
312	☑	☆	hypothetical protein TTHERM_01298560 [Tetrahymena thermophila]	gi|146184636	87 kDa		10	8
313	☑	☆	Protein kinase domain containing protein [Tetrahymena thermophila]	gi|118374967	53 kDa		10	8
314	☑	☆	MHCK/EF2 kinase domain family protein [Tetrahymena thermophila]	gi|118356595	104 kDa		10	8
315	☑	☆	RecName: Full=Histone H2B	gi|108885312 (+5)	14 kDa		9	8
316	☑	☆	Histone H4, minor, putative [Tetrahymena thermophila]	gi|118366755 (+5)	11 kDa	★	9	8
317	☑	☆	hypothetical protein TTHERM_01474510 [Tetrahymena thermophila]	gi|118394246	46 kDa		9	8
318	☑	☆	conserved hypothetical protein [Tetrahymena thermophila]	gi|146182176	143 kDa		9	8
319	☑	☆	Ribosomal protein S19e containing protein [Tetrahymena thermophila]	gi|118398280	18 kDa		8	8
320	☑	☆	ribosomal protein L10.e containing protein [Tetrahymena thermophila]	gi|118358504	24 kDa		8	8
321	☑	☆	ribosomal protein L6 containing protein [Tetrahymena thermophila]	gi|118395507	21 kDa		8	8
322	☑	☆	Cytochrome C1 family protein [Tetrahymena thermophila]	gi|118389517	25 kDa		8	8
323	☑	☆	hypothetical protein TTHERM_00530650 [Tetrahymena thermophila]	gi|146185942	25 kDa		8	8
324	☑	☆	hypothetical protein TTHERM_00283850 [Tetrahymena thermophila]	gi|146172036	24 kDa		8	8
325	☑	☆	hypothetical protein TTHERM_00047230 [Tetrahymena thermophila]	gi|146165274	41 kDa		8	8
326	☑	☆	hypothetical protein TTHERM_00591660 [Tetrahymena thermophila]	gi|146176574	108 kDa		8	8
327	☑	☆	hypothetical protein TTHERM_00649060 [Tetrahymena thermophila]	gi|118399953	39 kDa		7	8
328	☑	☆	hypothetical protein TTHERM_00312800 [Tetrahymena thermophila]	gi|118349169	35 kDa		7	8
329	☑	☆	DnaJ domain containing protein [Tetrahymena thermophila]	gi|118397435	37 kDa		7	8
330	☑	☆	ribosomal protein S8 containing protein [Tetrahymena thermophila]	gi|118360340	15 kDa		7	8
331	☑	☆	dehydrogenase, isocitrate/isopropylmalate family protein [Tetrahymena thermophila]	gi|146185448	72 kDa		7	8
332	☑	☆	rieske iron-sulfur protein, Ubiquinol-cytochrome c reductase iron-sulfur subunit [Tetrahymena thermophila]	gi|146164447	31 kDa		7	8
333	☑	☆	Ribosomal protein L13 containing protein [Tetrahymena thermophila]	gi|118377465	90 kDa		7	8

Probability Legend

- over 95%
- 80% to 94%
- 50% to 79%
- 20% to 49%
- 0% to 19%

#	Visible?	Starred?	Bio View: Identified Proteins (1837)	Accession Number	Molecular Weight	Protein Grouping Ambiguity	01 Control	02 Sample
334	✓	☆	Acyl-CoA dehydrogenase, C-terminal domain containing protein [Tetrahymena thermophila]	gi|118389496	71 kDa		7	8
335	✓	☆	ribosomal protein L22 [Tetrahymena thermophila]	gi|229595912	14 kDa		6	8
336	✓	☆	electron transfer flavoprotein, alpha subunit containing protein [Tetrahymena thermophila]	gi|118365094	37 kDa		6	8
337	✓	☆	hypothetical protein TTHERM_00487150 [Tetrahymena thermophila]	gi|118401212	84 kDa		6	8
338	✓	☆	MIF4G domain containing protein [Tetrahymena thermophila]	gi|118384672	95 kDa		6	8
339	✓	☆	ymf67 [Tetrahymena thermophila]	gi|15027665	54 kDa		6	8
340	✓	☆	TPR Domain containing protein [Tetrahymena thermophila]	gi|118349353	75 kDa	★	5	8
341	✓	☆	hypothetical protein TTHERM_00569310 [Tetrahymena thermophila]	gi|118389090	31 kDa		5	8
342	✓	☆	ribosomal protein L4/L1 family protein [Tetrahymena thermophila]	gi|146181037	42 kDa		5	8
343	✓	☆	Vacuolar protein sorting-associated protein 35 containing protein [Tetrahymena thermophila]	gi|118359812	157 kDa		5	8
344	✓	☆	Protein kinase domain containing protein [Tetrahymena thermophila]	gi|146186037	28 kDa		4	8
345	✓	☆	Protein kinase domain containing protein [Tetrahymena thermophila]	gi|118367727	74 kDa		4	8
346	✓	☆	ubiquitin-activating enzyme E1 family protein [Tetrahymena thermophila]	gi|118372041	124 kDa	★	4	8
347	✓	☆	hypothetical protein TTHERM_01207550 [Tetrahymena thermophila]	gi|146180995	51 kDa		4	8
348	✓	☆	Acyl-CoA oxidase family protein [Tetrahymena thermophila]	gi|118379591	77 kDa		4	8
349	✓	☆	hypothetical protein TTHERM_00668060 [Tetrahymena thermophila]	gi|118373764	67 kDa		4	8
350	✓	☆	Glutamate/Leucine/Phenylalanine/Valine dehydrogenase family protein [Tetrahymena thermophila]	gi|118384733	55 kDa		2	8
351	✓	☆	phosphoglycerate mutase 1 family protein [Tetrahymena thermophila]	gi|229595656	32 kDa			8
352	✓	☆	phenylalanyl-tRNA synthetase, beta subunit family protein [Tetrahymena thermophila]	gi|146179081	70 kDa			8
353	✓	☆	NAD synthase family protein [Tetrahymena thermophila]	gi|118381599	80 kDa			8
354	✓	☆	lysyl-tRNA synthetase family protein [Tetrahymena thermophila]	gi|118399585	76 kDa			8
355	✓	☆	Carbamoyl-phosphate synthase L chain, ATP binding domain containing protein [Tetrahymena thermophila]	gi|118378204	76 kDa			8
356	✓	☆	KH domain containing protein [Tetrahymena thermophila]	gi|146165238	86 kDa		25	7
357	✓	☆	hypothetical protein TTHERM_00299879 [Tetrahymena thermophila]	gi|146182399	123 kDa		24	7
358	✓	☆	hypothetical protein TTHERM_00919590 [Tetrahymena thermophila]	gi|118389537	72 kDa		22	7
359	✓	☆	hypothetical protein TTHERM_00013530 [Tetrahymena thermophila]	gi|118350102	94 kDa		20	7
360	✓	☆	DnaJ domain containing protein [Tetrahymena thermophila]	gi|229593791	47 kDa		17	7
361	✓	☆	DNA gyrase/topoisomerase IV, A subunit family protein [Tetrahymena thermophila]	gi|118398165	164 kDa		16	7
362	✓	☆	protein disulfide-isomerase domain containing protein [Tetrahymena thermophila]	gi|146182108	54 kDa		16	7
363	✓	☆	hypothetical protein TTHERM_00947420 [Tetrahymena thermophila]	gi|118397289	41 kDa		15	7
364	✓	☆	hypothetical protein TTHERM_00388620 [Tetrahymena thermophila]	gi|229595185	31 kDa		15	7
365	✓	☆	hypothetical protein TTHERM_00013110 [Tetrahymena thermophila]	gi|146161951	108 kDa		14	7
366	✓	☆	HEAT repeat family protein [Tetrahymena thermophila]	gi|146182851	66 kDa	★	14	7
367	✓	☆	calcium-translocating P-type ATPase, PMCA-type family protein [Tetrahymena thermophila]	gi|118387699	116 kDa	★	13	7
368	✓	☆	hypothetical protein TTHERM_00372420 [Tetrahymena thermophila]	gi|229596412	38 kDa		13	7
369	✓	☆	hypothetical protein TTHERM_00498220 [Tetrahymena thermophila]	gi|118389930	262 kDa		13	7
370	✓	☆	hypothetical protein TTHERM_01040830 [Tetrahymena thermophila]	gi|118385721	68 kDa	★	13	7

Probability Legend

- over 95%
- 80% to 94%
- 50% to 79%
- 20% to 49%
- 0% to 19%

#	Visible?	Starred?	Bio View: Identified Proteins (1837)	Accession Number	Molecular Weight	Protein Grouping Ambiguity	01 Control	02 Sample
371	☑	☆	Amidase family protein [Tetrahymena thermophila]	gi|118347742	73 kDa	★	13	7
372	☑	☆	26S proteasome subunit P45 family protein [Tetrahymena thermophila]	gi|146185054	142 kDa		12	7
373	☑	☆	26S proteasome subunit P45 family protein [Tetrahymena thermophila]	gi|118348546	51 kDa		12	7
374	☑	☆	hypothetical protein TTHERM_01027490 [Tetrahymena thermophila]	gi|146185003	38 kDa		12	7
375	☑	☆	Dynamin central region family protein [Tetrahymena thermophila]	gi|118401140	74 kDa		12	7
376	☑	☆	26S proteasome subunit P45 family protein [Tetrahymena thermophila]	gi|146162295	44 kDa	★	11	7
377	☑	☆	hypothetical protein TTHERM_01093510 [Tetrahymena thermophila]	gi|229593988	50 kDa		11	7
378	☑	☆	hypothetical protein TTHERM_00355820 [Tetrahymena thermophila]	gi|118354435	53 kDa		11	7
379	☑	☆	Protein phosphatase 2C containing protein [Tetrahymena thermophila]	gi|118388502	47 kDa		10	7
380	☑	☆	aspartyl-tRNA synthetase family protein [Tetrahymena thermophila]	gi|118383039	70 kDa		10	7
381	☑	☆	hypothetical protein TTHERM_00584850 [Tetrahymena thermophila]	gi|118400538	61 kDa		10	7
382	☑	☆	hypothetical protein TTHERM_00939150 [Tetrahymena thermophila]	gi|118397513	27 kDa		9	7
383	☑	☆	hypothetical protein TTHERM_00038880 [Tetrahymena thermophila]	gi|118346459 (+1)	44 kDa		9	7
384	☑	☆	Protein kinase domain containing protein [Tetrahymena thermophila]	gi|118385913	102 kDa		9	7
385	☑	☆	26S proteasome non-ATPase regulatory subunit Nin1/mts3 family protein [Tetrahymena thermophila]	gi|146185674	33 kDa		9	7
386	☑	☆	hypothetical protein TTHERM_00853120 [Tetrahymena thermophila]	gi|229593876	49 kDa		9	7
387	☑	☆	Mitochondrial carrier protein [Tetrahymena thermophila]	gi|118380639	33 kDa		8	7
388	☑	☆	RecName: Full=60S ribosomal protein L5	gi|353678153	34 kDa		8	7
389	☑	☆	hypothetical protein TTHERM_00686220 [Tetrahymena thermophila]	gi|229594186	15 kDa		8	7
390	☑	☆	hypothetical protein TTHERM_00193750 [Tetrahymena thermophila]	gi|146169365	22 kDa		8	7
391	☑	☆	ribosomal protein S17 containing protein [Tetrahymena thermophila]	gi|118395679	18 kDa		7	7
392	☑	☆	hypothetical protein TTHERM_00136220 [Tetrahymena thermophila]	gi|118373018	36 kDa		7	7
393	☑	☆	Thioredoxin family protein [Tetrahymena thermophila]	gi|118389048	24 kDa		7	7
394	☑	☆	hypothetical protein TTHERM_00804710 [Tetrahymena thermophila]	gi|118358348	248 kDa		7	7
395	☑	☆	hypothetical protein TTHERM_00624630 [Tetrahymena thermophila]	gi|229594675	42 kDa		7	7
396	☑	☆	hypothetical protein TTHERM_00481310 [Tetrahymena thermophila]	gi|118368471	43 kDa	★	6	7
397	☑	☆	Plectin/S10 domain containing protein [Tetrahymena thermophila]	gi|118387069	19 kDa		6	7
398	☑	☆	Mitochondrial carrier protein [Tetrahymena thermophila]	gi|146163822	32 kDa		6	7
399	☑	☆	Ras family protein [Tetrahymena thermophila]	gi|146180701	24 kDa	★	6	7
400	☑	☆	hypothetical protein TTHERM_00006120 [Tetrahymena thermophila]	gi|146161872	16 kDa		6	7
401	☑	☆	Adaptin N-terminal region family protein [Tetrahymena thermophila]	gi|146168466	112 kDa		5	7
402	☑	☆	electron transfer flavoprotein, beta subunit containing protein [Tetrahymena thermophila]	gi|118365591	28 kDa		5	7
403	☑	☆	SPFH domain / Band 7 family protein [Tetrahymena thermophila]	gi|229593978	31 kDa		5	7
404	☑	☆	Ribosomal protein L7/L12 C-terminal domain containing protein [Tetrahymena thermophila]	gi|146184118	31 kDa		5	7
405	☑	☆	IBR domain containing protein [Tetrahymena thermophila]	gi|146184580	138 kDa		5	7
406	☑	☆	Coatomer WD associated domain containing protein [Tetrahymena thermophila]	gi|118394671	141 kDa		5	7
407	☑	☆	Ubiquitin carboxyl-terminal hydrolase family protein [Tetrahymena thermophila]	gi|118375166	92 kDa		5	7

Probability Legend

- over 95%
- 80% to 94%
- 50% to 79%
- 20% to 49%
- 0% to 19%

#	Visible?	Starred?	Bio View: Identified Proteins (1837)	Accession Number	Molecular Weight	Protein Grouping Ambiguity	01 Control	02 Sample
408	☑	☆	conserved hypothetical protein [Tetrahymena thermophila]	gi|118373688	84 kDa		5	7
409	☑	☆	conserved hypothetical protein [Tetrahymena thermophila]	gi|146163301	45 kDa		5	7
410	☑	☆	hypothetical protein TTHERM_00437740 [Tetrahymena thermophila]	gi|118369130	107 kDa		4	7
411	☑	☆	PCI domain containing protein [Tetrahymena thermophila]	gi|146162579	52 kDa		4	7
412	☑	☆	hypothetical protein TTHERM_01000190 [Tetrahymena thermophila]	gi|118388745	43 kDa		3	7
413	☑	☆	hypothetical protein TTHERM_00050600 [Tetrahymena thermophila]	gi|118362690	448 kDa		3	7
414	☑	☆	hypothetical protein TTHERM_00840040 [Tetrahymena thermophila]	gi|118384255	59 kDa		3	7
415	☑	☆	hypothetical protein TTHERM_00471090 [Tetrahymena thermophila]	gi|146186089	23 kDa			7
416	☑	☆	MIF4G domain containing protein [Tetrahymena thermophila]	gi|118361742	105 kDa			7
417	☑	☆	ribosomal protein L24 containing protein [Tetrahymena thermophila]	gi|118385021	27 kDa			7
418	☑	☆	ribosomal protein L28 containing protein [Tetrahymena thermophila]	gi|146163214	35 kDa			7
419	☑	☆	U-box domain containing protein [Tetrahymena thermophila]	gi|118348690	89 kDa			7
420	☑	☆	Kinesin motor domain containing protein [Tetrahymena thermophila]	gi|146161602	143 kDa		30	7
421	☑	☆	eukaryotic peptide chain release factor subunit 1 [Tetrahymena thermophila]	gi|118371070	50 kDa			7
422	☑	☆	intraflagellar transport protein IFT172 [Tetrahymena thermophila]	gi|152206039	197 kDa		26	6
423	☑	☆	hypothetical protein TTHERM_00637030 [Tetrahymena thermophila]	gi|146185711	110 kDa		22	6
424	☑	☆	ThiF family protein [Tetrahymena thermophila]	gi|146175236	76 kDa		17	6
425	☑	☆	hypothetical protein TTHERM_00531890 [Tetrahymena thermophila]	gi|118382313	117 kDa		17	6
426	☑	☆	hypothetical protein TTHERM_00758980 [Tetrahymena thermophila]	gi|118367535	257 kDa		16	6
427	☑	☆	isochorismatase family protein [Tetrahymena thermophila]	gi|118355340	106 kDa		11	6
428	☑	☆	hypothetical protein TTHERM_00394410 [Tetrahymena thermophila]	gi|118357327	75 kDa		11	6
429	☑	☆	oxidoreductase, FAD/FMN-binding family protein [Tetrahymena thermophila]	gi|118353081	63 kDa		10	6
430	☑	☆	hypothetical protein TTHERM_00348410 [Tetrahymena thermophila]	gi|146181590	219 kDa		10	6
431	☑	☆	TCP-1/cpn60 chaperonin family protein [Tetrahymena thermophila]	gi|146169799	62 kDa		10	6
432	☑	☆	PRP38 family protein [Tetrahymena thermophila]	gi|118384163	332 kDa		9	6
433	☑	☆	26S proteasome subunit P45 family protein [Tetrahymena thermophila]	gi|118383904	44 kDa		9	6
434	☑	☆	hypothetical protein TTHERM_00891190 [Tetrahymena thermophila]	gi|118374242	84 kDa		9	6
435	☑	☆	Electron transfer flavoprotein-ubiquinone oxidoreductase containing protein [Tetrahymena thermophila]	gi|146181753	135 kDa		9	6
436	☑	☆	Ribosomal protein S30 containing protein [Tetrahymena thermophila]	gi|146184240	9 kDa		8	6
437	☑	☆	granule lattice protein 3 precursor, putative [Tetrahymena thermophila]	gi|146181358 (+1)	41 kDa		8	6
438	☑	☆	Na,H/K antiporter P-type ATPase, alpha subunit family protein [Tetrahymena thermophila]	gi|118381158	135 kDa	★	8	6
439	☑	☆	hypothetical protein TTHERM_00557760 [Tetrahymena thermophila]	gi|118378329	46 kDa		8	6
440	☑	☆	hypothetical protein TTHERM_01055600 [Tetrahymena thermophila]	gi|229593639	44 kDa		7	6
441	☑	☆	Ribosomal protein L32 containing protein [Tetrahymena thermophila]	gi|118358848	15 kDa		7	6
442	☑	☆	hypothetical protein TTHERM_01005010 [Tetrahymena thermophila]	gi|118354116	31 kDa		7	6
443	☑	☆	succinate dehydrogenase and fumarate reductase iron-sulfur protein [Tetrahymena thermophila]	gi|146182596	36 kDa		7	6
444	☑	☆	hypothetical protein TTHERM_00194499 [Tetrahymena thermophila]	gi|146169553	23 kDa		7	6

Probability Legend

- over 95%
- 80% to 94%
- 50% to 79%
- 20% to 49%
- 0% to 19%

#	Visible?	Starred?	Bio View: Identified Proteins (1837)	Accession Number	Molecular Weight	Protein Grouping Ambiguity	01 Control	02 Sample
445	☑	☆	hypothetical protein TTHERM_00338280 [Tetrahymena thermophila]	gi|146170576	47 kDa		7	6
446	☑	☆	splicing factor, CC1-like family protein [Tetrahymena thermophila]	gi|118357832	66 kDa		7	6
447	☑	☆	Phosphofructokinase family protein [Tetrahymena thermophila]	gi|146170620	61 kDa		7	6
448	☑	☆	V-type ATPase 116kDa subunit family protein [Tetrahymena thermophila]	gi|118354156	99 kDa		7	6
449	☑	☆	hypothetical protein TTHERM_01161000 [Tetrahymena thermophila]	gi|118370180	24 kDa		7	6
450	☑	☆	hypothetical protein TTHERM_00266390 [Tetrahymena thermophila]	gi|118365307	16 kDa		6	6
451	☑	☆	hypothetical protein TTHERM_00382350 [Tetrahymena thermophila]	gi|146165636	15 kDa		6	6
452	☑	☆	ribosomal protein L15 [Tetrahymena thermophila]	gi|118394925	24 kDa		6	6
453	☑	☆	hypothetical protein TTHERM_00075670 [Tetrahymena thermophila]	gi|118364772	11 kDa		6	6
454	☑	☆	hypothetical protein TTHERM_00522600 [Tetrahymena thermophila]	gi|229595893	42 kDa		6	6
455	☑	☆	Thioredoxin-dependent peroxide reductase, mitochondrial precursor, putative [Tetrahymena thermophila]	gi|118359822	23 kDa		6	6
456	☑	☆	hypothetical protein TTHERM_00561680 [Tetrahymena thermophila]	gi|146162841	14 kDa		6	6
457	☑	☆	ribosomal protein L15 containing protein [Tetrahymena thermophila]	gi|146168461 (+1)	25 kDa		6	6
458	☑	☆	hypothetical protein TTHERM_00268000 [Tetrahymena thermophila]	gi|118365429	40 kDa		6	6
459	☑	☆	hypothetical protein TTHERM_00295430 [Tetrahymena thermophila]	gi|118359874	25 kDa		6	6
460	☑	☆	succinyl-CoA synthetase, beta subunit family protein [Tetrahymena thermophila]	gi|118369102	50 kDa		6	6
461	☑	☆	emp24/gp25L/p24 family protein [Tetrahymena thermophila]	gi|146184833	24 kDa		6	6
462	☑	☆	V-type ATPase 116kDa subunit family protein [Tetrahymena thermophila]	gi|146166001	100 kDa		6	6
463	☑	☆	ribosomal protein L13 containing protein [Tetrahymena thermophila]	gi|146181945 (+1)	31 kDa		5	6
464	☑	☆	RNA binding protein [Tetrahymena thermophila]	gi|118381832	35 kDa		5	6
465	☑	☆	hypothetical protein TTHERM_00068120 [Tetrahymena thermophila]	gi|146161614	25 kDa		5	6
466	☑	☆	zinc finger protein [Tetrahymena thermophila]	gi|118399961	46 kDa		5	6
467	☑	☆	hypothetical protein TTHERM_00532090 [Tetrahymena thermophila]	gi|118382349	26 kDa		5	6
468	☑	☆	hypothetical protein TTHERM_00571670 [Tetrahymena thermophila]	gi|146184052	24 kDa		5	6
469	☑	☆	Protein kinase domain containing protein [Tetrahymena thermophila]	gi|118358856	72 kDa		5	6
470	☑	☆	EF hand family protein [Tetrahymena thermophila]	gi|146161610 (+1)	25 kDa		4	6
471	☑	☆	succinyl-CoA synthetase, beta subunit family protein [Tetrahymena thermophila]	gi|118365146	60 kDa		4	6
472	☑	☆	protein phosphatase 2A regulatory B subunit (B56 family) [Tetrahymena thermophila]	gi|146182357	61 kDa		4	6
473	☑	☆	hypothetical protein TTHERM_01079250 [Tetrahymena thermophila]	gi|118385110	68 kDa		4	6
474	☑	☆	Initiation factor 2 subunit family protein [Tetrahymena thermophila]	gi|118377939	36 kDa		3	6
475	☑	☆	hypothetical protein TTHERM_00377330 [Tetrahymena thermophila]	gi|118364383	68 kDa		3	6
476	☑	☆	Protein kinase domain containing protein [Tetrahymena thermophila]	gi|146176302	54 kDa		3	6
477	☑	☆	peptidase, S49 (protease IV) family protein [Tetrahymena thermophila]	gi|118382549	21 kDa		2	6
478	☑	☆	SLEI family protein [Tetrahymena thermophila]	gi|118395433	282 kDa	★	2	6
479	☑	☆	GTP-binding protein YchF containing protein [Tetrahymena thermophila]	gi|118382824	43 kDa		2	6
480	☑	☆	hypothetical protein TTHERM_00046360 [Tetrahymena thermophila]	gi|118363488	123 kDa		2	6
481	☑	☆	enolase family protein [Tetrahymena thermophila]	gi|118401078	48 kDa			6

Probability Legend

- over 95%
- 80% to 94%
- 50% to 79%
- 20% to 49%
- 0% to 19%

#	Visible?	Starred?	Bio View: Identified Proteins (1837)	Accession Number	Molecular Weight	Protein Grouping Ambiguity	01 Control	02 Sample
482	☑	☆	Acyl-CoA dehydrogenase, C-terminal domain containing protein [Tetrahymena thermophila]	gi|118348970	89 kDa			6
483	☑	☆	Phosphoenolpyruvate carboxylase, putative [Tetrahymena thermophila]	gi|118377471	107 kDa			6
484	☑	☆	aminotransferase, classes I and II family protein [Tetrahymena thermophila]	gi|146169134	48 kDa			6
485	☑	☆	Isoamylase N-terminal domain containing protein [Tetrahymena thermophila]	gi|146176814	79 kDa			6
486	☑	☆	hypothetical protein TTHERM_00649180 [Tetrahymena thermophila]	gi|229595597	52 kDa		13	6
487	☑	☆	hypothetical protein TTHERM_00227580 [Tetrahymena thermophila]	gi|146164890	54 kDa		10	6
488	☑	☆	hypothetical protein TTHERM_00250960 [Tetrahymena thermophila]	gi|118371696	28 kDa		9	6
489	☑	☆	hypothetical protein TTHERM_00086700 [Tetrahymena thermophila]	gi|118358954	85 kDa		9	6
490	☑	☆	SPFH domain / Band 7 family protein [Tetrahymena thermophila]	gi|118357197	43 kDa	★	6	6
491	☑	☆	Ubiquitin-conjugating enzyme family protein [Tetrahymena thermophila]	gi|146170202	309 kDa	★	6	6
492	☑	☆	Adaptin N-terminal region family protein [Tetrahymena thermophila]	gi|146181717	105 kDa		6	6
493	☑	☆	hypothetical protein TTHERM_01188360 [Tetrahymena thermophila]	gi|118360532	18 kDa		6	6
494	☑	☆	ATP synthase F1, delta subunit family protein [Tetrahymena thermophila]	gi|146182760	25 kDa		6	6
495	☑	☆	Domain found in IF2B/IF5 family protein [Tetrahymena thermophila]	gi|146183450	24 kDa		4	6
496	☑	☆	GFP-BSD [synthetic construct]	gi|386372757	45 kDa			6
497	☑	☆	hypothetical protein TTHERM_00773720 [Tetrahymena thermophila]	gi|229594564	40 kDa			6
498	☑	☆	AMP-binding enzyme family protein [Tetrahymena thermophila]	gi|146182536	73 kDa	★		6
499	☑	☆	Alpha amylase, catalytic domain containing protein [Tetrahymena thermophila]	gi|118394367	86 kDa			6
500	☑	☆	hypothetical protein TTHERM_00641200 [Tetrahymena thermophila]	gi|118364230	198 kDa		17	6
501	☑	☆	hypothetical protein TTHERM_00299970 [Tetrahymena thermophila]	gi|118382736	136 kDa		7	6
502	☑	☆	Leucine Rich Repeat family protein [Tetrahymena thermophila]	gi|118399424	69 kDa		2	6
503	☑	☆	PCI domain containing protein [Tetrahymena thermophila]	gi|118368159	45 kDa		2	6
504	☑	☆	peptidase, S49 (protease IV) family protein [Tetrahymena thermophila]	gi|146174461	29 kDa			6
505	☑	☆	hypothetical protein TTHERM_00823650 [Tetrahymena thermophila]	gi|118398276	20 kDa		5	6
506	☑	☆	hypothetical protein TTHERM_00474900 [Tetrahymena thermophila]	gi|118381623	70 kDa		4	6
507	☑	☆	Protein kinase domain containing protein [Tetrahymena thermophila]	gi|146161707	54 kDa			6
508	☑	☆	RecName: Full=High mobility group protein C; AltName: Full=Non-histone chromosomal protein LG-1	gi|126226	12 kDa		3	6
509	☑	☆	ATPase, AAA family protein [Tetrahymena thermophila]	gi|146161282	50 kDa	★	9	6
510	☑	☆	hypothetical protein TTHERM_00773780 [Tetrahymena thermophila]	gi|118398606	237 kDa			6
511	☑	☆	hypothetical protein TTHERM_00470570 [Tetrahymena thermophila]	gi|229595000	29 kDa		5	6
512	☑	☆	Dual specificity phosphatase, catalytic domain containing protein [Tetrahymena thermophila]	gi|146186247	102 kDa		2	6
513	☑	☆	aldehyde dehydrogenase [Tetrahymena thermophila]	gi|118400829	56 kDa		15	5
514	☑	☆	Peroxidase family protein [Tetrahymena thermophila]	gi|118372870	33 kDa		8	5
515	☑	☆	pyruvate dehydrogenase complex dihydrolipoamide acetyltransferase family protein [Tetrahymena thermophila]	gi|229594592	67 kDa		7	5
516	☑	☆	hypothetical protein TTHERM_00421060 [Tetrahymena thermophila]	gi|118402081	22 kDa		7	5
517	☑	☆	Ribosomal L18ae protein family protein [Tetrahymena thermophila]	gi|146170063 (+1)	29 kDa		6	5
518	☑	☆	RecName: Full=60S ribosomal protein L28	gi|353678076	15 kDa		6	5

Probability Legend

- over 95%
- 80% to 94%
- 50% to 79%
- 20% to 49%
- 0% to 19%

#	Visible?	Starred?	Bio View: Identified Proteins (1837)	Accession Number	Molecular Weight	Protein Grouping Ambiguity	01 Control	02 Sample
519	☑	☆	60S ribosomal protein L21, putative [Tetrahymena thermophila]	gi|229595108	18 kDa		6	5
520	☑	☆	Ribosomal protein L19e containing protein [Tetrahymena thermophila]	gi|146181731	21 kDa		5	5
521	☑	☆	ribosomal protein L23 containing protein [Tetrahymena thermophila]	gi|118372960	17 kDa		5	5
522	☑	☆	hypothetical protein TTHERM_00338210 [Tetrahymena thermophila]	gi|146170551 (+1)	88 kDa		4	5
523	☑	☆	Ribosomal protein L36e containing protein [Tetrahymena thermophila]	gi|118387091	12 kDa		4	5
524	☑	☆	Ribosomal protein S27a containing protein [Tetrahymena thermophila]	gi|118384128	21 kDa		3	5
525	☑	☆	ribosomal protein S10 containing protein [Tetrahymena thermophila]	gi|118397210	14 kDa		2	5
526	☑	☆	Mov34/MPN/PAD-1 family protein [Tetrahymena thermophila]	gi|146180804	33 kDa		2	5
527	☑	☆	hypothetical protein TTHERM_00967540 [Tetrahymena thermophila]	gi|118351335	210 kDa	★	24	5
528	☑	☆	Ribosomal Proteins L2, C-terminal domain containing protein [Tetrahymena thermophila]	gi|146185506	29 kDa		6	5
529	☑	☆	hypothetical protein TTHERM_00653670 [Tetrahymena thermophila]	gi|229595957	16 kDa		6	5
530	☑	☆	Cyclin, N-terminal domain containing protein [Tetrahymena thermophila]	gi|146168287	105 kDa		5	5
531	☑	☆	Mitochondrial ribosomal protein L51 / S25 / CI-B8 domain containing protein [Tetrahymena thermophila]	gi|118350252	26 kDa			5
532	☑	☆	RNA polymerase Rpb1, domain 2 family protein [Tetrahymena thermophila]	gi|118346269	162 kDa			5
533	☑	☆	hypothetical protein TTHERM_00220600 [Tetrahymena thermophila]	gi|118374889	41 kDa			5
534	☑	☆	Proteasome/cyclosome repeat family protein [Tetrahymena thermophila]	gi|118401628	108 kDa		15	5
535	☑	☆	Adaptor complexes medium subunit family protein [Tetrahymena thermophila]	gi|118352240	49 kDa		10	5
536	☑	☆	Sodium/hydrogen exchanger family protein [Tetrahymena thermophila]	gi|146165774	113 kDa		8	5
537	☑	☆	Cyclin, N-terminal domain containing protein [Tetrahymena thermophila]	gi|118386187	30 kDa		7	5
538	☑	☆	hypothetical protein TTHERM_00420130 [Tetrahymena thermophila]	gi|118401897	22 kDa		5	5
539	☑	☆	hypothetical protein TTHERM_00456720 [Tetrahymena thermophila]	gi|146182232	213 kDa			5
540	☑	☆	Cullin family protein [Tetrahymena thermophila]	gi|118358670	90 kDa			5
541	☑	☆	hypothetical protein TTHERM_00255660 [Tetrahymena thermophila]	gi|118371844	108 kDa		17	5
542	☑	☆	hypothetical protein TTHERM_00139640 [Tetrahymena thermophila]	gi|118373306	86 kDa		13	5
543	☑	☆	Dentin sialophosphoprotein precursor, putative [Tetrahymena thermophila]	gi|146185850	57 kDa		10	5
544	☑	☆	hypothetical protein TTHERM_01001220 [Tetrahymena thermophila]	gi|146183972	19 kDa		5	5
545	☑	☆	protein phosphatase regulatory subunit [Tetrahymena thermophila]	gi|146185803	48 kDa		5	5
546	☑	☆	aspartate aminotransferase [Tetrahymena thermophila]	gi|118367139	46 kDa			5
547	☑	☆	NADH dehydrogenase subunit 7 [Tetrahymena thermophila]	gi|15027641	51 kDa		5	5
548	☑	☆	dehydrogenase, isocitrate/isopropylmalate family protein [Tetrahymena thermophila]	gi|146172422	40 kDa		11	5
549	☑	☆	predicted protein [Tetrahymena thermophila]	gi|229594460	83 kDa		8	5
550	☑	☆	oxidoreductase, short chain dehydrogenase/reductase family protein [Tetrahymena thermophila]	gi|118400041	37 kDa		8	5
551	☑	☆	Glutathione S-transferase, N-terminal domain containing protein [Tetrahymena thermophila]	gi|118361714	46 kDa		6	5
552	☑	☆	hypothetical protein TTHERM_00675630 [Tetrahymena thermophila]	gi|118363621	17 kDa		5	5
553	☑	☆	hypothetical protein TTHERM_00402070 [Tetrahymena thermophila]	gi|118361704	25 kDa		5	5
554	☑	☆	ribosomal protein S11 containing protein [Tetrahymena thermophila]	gi|146182837 (+2)	16 kDa		4	5
555	☑	☆	hypothetical protein TTHERM_00456800 [Tetrahymena thermophila]	gi|229594332	88 kDa		4	5

Probability Legend

- over 95%
- 80% to 94%
- 50% to 79%
- 20% to 49%
- 0% to 19%

#	Visible?	Starred?	Bio View: Identified Proteins (1837)	Accession Number	Molecular Weight	Protein Grouping Ambiguity	01 Control	02 Sample
556	☑	☆	GAT domain containing protein [Tetrahymena thermophila]	gi|118354235	79 kDa		2	5
557	☑	☆	conserved hypothetical protein [Tetrahymena thermophila]	gi|146165759	41 kDa			5
558	☑	☆	ribosomal protein S16 containing protein [Tetrahymena thermophila]	gi|118380262	55 kDa			5
559	☑	☆	hypothetical protein TTHERM_01113100 [Tetrahymena thermophila]	gi|118385633	38 kDa			5
560	☑	☆	NADH-quinone oxidoreductase, F subunit family protein [Tetrahymena thermophila]	gi|118367869	52 kDa		9	5
561	☑	☆	hypothetical protein TTHERM_00133510 [Tetrahymena thermophila]	gi|118372872	21 kDa		5	5
562	☑	☆	hypothetical protein TTHERM_00429890 [Tetrahymena thermophila]	gi|229594632	42 kDa		5	5
563	☑	☆	hypothetical protein TTHERM_01310170 [Tetrahymena thermophila]	gi|118387128	113 kDa			5
564	☑	☆	hypothetical protein TTHERM_00455090 [Tetrahymena thermophila]	gi|229594325	23 kDa		5	5
565	☑	☆	hypothetical protein TTHERM_00601860 [Tetrahymena thermophila]	gi|229595761	20 kDa		3	5
566	☑	☆	HECT domain and RCC1-like domain-containing protein [Tetrahymena thermophila]	gi|118368035	619 kDa			5
567	☑	☆	TPR Domain containing protein [Tetrahymena thermophila]	gi|118388809	58 kDa			5
568	☑	☆	hypothetical protein TTHERM_00849310 [Tetrahymena thermophila]	gi|118372072	181 kDa		8	5
569	☑	☆	PX domain containing protein [Tetrahymena thermophila]	gi|118352781	52 kDa		3	5
570	☑	☆	hypothetical protein TTHERM_00106969 [Tetrahymena thermophila]	gi|146163808	28 kDa		3	5
571	☑	☆	hypothetical protein TTHERM_00161050 [Tetrahymena thermophila]	gi|118353129	68 kDa		3	5
572	☑	☆	Fructose-bisphosphate aldolase class-I family protein [Tetrahymena thermophila]	gi|118401200	39 kDa			5
573	☑	☆	KOW motif family protein [Tetrahymena thermophila]	gi|118346425	90 kDa		12	5
574	☑	☆	hypothetical protein TTHERM_00568030 [Tetrahymena thermophila]	gi|229594480	30 kDa		5	5
575	☑	☆	ribosomal protein S25 [Tetrahymena thermophila]	gi|146163441	16 kDa		5	5
576	☑	☆	KH domain containing protein [Tetrahymena thermophila]	gi|118363004	80 kDa		12	5
577	☑	☆	Ribosomal protein L14p/L23e containing protein [Tetrahymena thermophila]	gi|146164168	15 kDa		6	5
578	☑	☆	hypothetical protein TTHERM_00691100 [Tetrahymena thermophila]	gi|118399503	18 kDa		6	5
579	☑	☆	hypothetical protein TTHERM_00218570 [Tetrahymena thermophila]	gi|146179156	20 kDa		6	5
580	☑	☆	conserved hypothetical protein [Tetrahymena thermophila]	gi|118376634	75 kDa		5	5
581	☑	☆	actin [Tetrahymena thermophila]	gi|118375955	45 kDa		2	5
582	☑	☆	methylmalonate-semialdehyde dehydrogenase family protein [Tetrahymena thermophila]	gi|146182393	58 kDa			5
583	☑	☆	HEAT repeat family protein [Tetrahymena thermophila]	gi|118354303	74 kDa	★	8	5
584	☑	☆	hypothetical protein TTHERM_00497450 [Tetrahymena thermophila]	gi|118389778	41 kDa		2	5
585	☑	☆	fimbrin-like 71 K protein [Tetrahymena thermophila]	gi|118373076 (+1)	59 kDa			5
586	☑	☆	AhpC/TSA family protein [Tetrahymena thermophila]	gi|118395270	23 kDa		5	5
587	☑	☆	hypothetical protein TTHERM_00492680 [Tetrahymena thermophila]	gi|118379991	56 kDa		17	5
588	☑	☆	hypothetical protein TTHERM_00525160 [Tetrahymena thermophila]	gi|118390071	39 kDa		5	5
589	☑	☆	hypothetical protein TTHERM_00077780 [Tetrahymena thermophila]	gi|118364994	74 kDa			5
590	☑	☆	hypothetical protein TTHERM_00637020 [Tetrahymena thermophila]	gi|118400080	23 kDa		8	5
591	☑	☆	hypothetical protein TTHERM_00760790 [Tetrahymena thermophila]	gi|118398735	65 kDa		7	5
592	☑	☆	hydrolase, alpha/beta fold family protein [Tetrahymena thermophila]	gi|118347501	36 kDa			5

Probability Legend

- over 95%
- 80% to 94%
- 50% to 79%
- 20% to 49%
- 0% to 19%

#	Visible?	Starred?	Bio View: Identified Proteins (1837)	Accession Number	Molecular Weight	Protein Grouping Ambiguity	01 Control	02 Sample
593	☑	☆	hypothetical protein TTHERM_00433490 [Tetrahymena thermophila]	gi|146163410	10 kDa		5	5
594	☑	☆	PX domain containing protein [Tetrahymena thermophila]	gi|146179470	54 kDa		6	5
595	☑	☆	hypothetical protein TTHERM_01232260 [Tetrahymena thermophila]	gi|118385876	366 kDa		12	5
596	☑	☆	hypothetical protein TTHERM_00703510 [Tetrahymena thermophila]	gi|229594310	49 kDa		9	5
597	☑	☆	ADP-ribosylation factor, arf [Tetrahymena thermophila]	gi|118394673	20 kDa		5	5
598	☑	☆	Thiolase, N-terminal domain containing protein [Tetrahymena thermophila]	gi|118368061	47 kDa		3	5
599	☑	☆	hypothetical protein TTHERM_00659010 [Tetrahymena thermophila]	gi|118381884	48 kDa			5
600	☑	☆	WGR domain containing protein [Tetrahymena thermophila]	gi|229593699	71 kDa			5
601	☑	☆	Protein kinase domain containing protein [Tetrahymena thermophila]	gi|146170271	36 kDa		5	5
602	☑	☆	Protein kinase domain containing protein [Tetrahymena thermophila]	gi|146177486	45 kDa			5
603	☑	☆	ATP-dependent helicase DHX8, RNA helicase HRH1 [Tetrahymena thermophila]	gi|118380258	148 kDa		4	5
604	☑	☆	PCI domain containing protein [Tetrahymena thermophila]	gi|146165552	26 kDa			5
605	☑	☆	MCM2/3/5 family protein [Tetrahymena thermophila]	gi|146162314	105 kDa		18	5
606	☑	☆	IBR domain containing protein [Tetrahymena thermophila]	gi|146182786	63 kDa		2	5
607	☑	☆	hypothetical protein TTHERM_00992730 [Tetrahymena thermophila]	gi|146185097 (+1)	66 kDa		5	4
608	☑	☆	ribosomal protein S15 containing protein [Tetrahymena thermophila]	gi|146185324	18 kDa		5	4
609	☑	☆	60S ribosomal protein L31, putative [Tetrahymena thermophila]	gi|118397418	13 kDa		5	4
610	☑	☆	RecName: Full=Ubiquitin-60S ribosomal protein L40; Contains: RecName: Full=Ubiquitin; Contains: RecName: Full=60S ribosomal protei...	gi|302393730	15 kDa		4	4
611	☑	☆	hypothetical protein TTHERM_00024050 [Tetrahymena thermophila]	gi|118350420	162 kDa			4
612	☑	☆	hypothetical protein TTHERM_00158000 [Tetrahymena thermophila]	gi|118352915	27 kDa		2	4
613	☑	☆	RNA polymerase Rpb5, C-terminal domain containing protein [Tetrahymena thermophila]	gi|118387602	33 kDa			4
614	☑	☆	Protein kinase domain containing protein [Tetrahymena thermophila]	gi|118399669	70 kDa	★	8	4
615	☑	☆	Sec23/Sec24 trunk domain containing protein [Tetrahymena thermophila]	gi|118364676	106 kDa			4
616	☑	☆	hypothetical protein TTHERM_00381080 [Tetrahymena thermophila]	gi|118364539	125 kDa		4	4
617	☑	☆	hypothetical protein TTHERM_00028510 [Tetrahymena thermophila]	gi|118346891	9 kDa		3	4
618	☑	☆	Ribosomal protein L7Ae containing protein [Tetrahymena thermophila]	gi|146181778	11 kDa		3	4
619	☑	☆	metallopeptidase family M24 containing protein [Tetrahymena thermophila]	gi|146171896 (+1)	117 kDa		13	4
620	☑	☆	EF hand family protein [Tetrahymena thermophila]	gi|118372191	19 kDa		7	4
621	☑	☆	Dynamin central region family protein [Tetrahymena thermophila]	gi|118366703	110 kDa		10	4
622	☑	☆	hypothetical protein TTHERM_00295790 [Tetrahymena thermophila]	gi|118359946	33 kDa		5	4
623	☑	☆	hypothetical protein TTHERM_00697490 [Tetrahymena thermophila]	gi|146182954	15 kDa		5	4
624	☑	☆	hypothetical protein TTHERM_00734110 [Tetrahymena thermophila]	gi|118356607	89 kDa		13	4
625	☑	☆	Mov34/MPN/PAD-1 family protein [Tetrahymena thermophila]	gi|118365427	37 kDa		9	4
626	☑	☆	Protein will die slowly, putative [Tetrahymena thermophila]	gi|146186232	44 kDa		6	4
627	☑	☆	Transketolase, pyridine binding domain containing protein [Tetrahymena thermophila]	gi|146163023	72 kDa			4
628	☑	☆	V-type ATPase, D subunit family protein [Tetrahymena thermophila]	gi|146185304	29 kDa			4
629	☑	☆	hypothetical protein TTHERM_00829480 [Tetrahymena thermophila]	gi|118360840	141 kDa	★	9	4

Probability Legend

- over 95%
- 80% to 94%
- 50% to 79%
- 20% to 49%
- 0% to 19%

#	Visible?	Starred?	Bio View: Identified Proteins (1837)	Accession Number	Molecular Weight	Protein Grouping Ambiguity	01 Control	02 Sample
630	☑	☆	hypothetical protein TTHERM_00268010 [Tetrahymena thermophila]	gi|118365431	162 kDa		15	4
631	☑	☆	DEAD/DEAH box helicase family protein [Tetrahymena thermophila]	gi|146175019	163 kDa		2	4
632	☑	☆	hypothetical protein TTHERM_00402050 [Tetrahymena thermophila]	gi|229595943	64 kDa		5	4
633	☑	☆	small GTP-binding protein domain containing protein [Tetrahymena thermophila]	gi|118356635	20 kDa		5	4
634	☑	☆	Leucine Rich Repeat family protein [Tetrahymena thermophila]	gi|118367919	70 kDa		11	4
635	☑	☆	DEAD/DEAH box helicase family protein [Tetrahymena thermophila]	gi|118385791	53 kDa			4
636	☑	☆	hypothetical protein TTHERM_00797930 [Tetrahymena thermophila]	gi|146178520	80 kDa			4
637	☑	☆	EF hand family protein [Tetrahymena thermophila]	gi|118389806	18 kDa	★	4	4
638	☑	☆	conserved hypothetical protein [Tetrahymena thermophila]	gi|118376652	17 kDa		8	4
639	☑	☆	Dolichyl-diphosphooligosaccharide-protein glycosyltransferase 48kD subunit containing protein [Tetrahymena thermophila]	gi|118361324	50 kDa		11	4
640	☑	☆	AMP-binding enzyme family protein [Tetrahymena thermophila]	gi|118363068	76 kDa	★	2	4
641	☑	☆	hypothetical protein TTHERM_00497620 [Tetrahymena thermophila]	gi|118389810	30 kDa	★		4
642	☑	☆	hypothetical protein TTHERM_01094890 [Tetrahymena thermophila]	gi|118355322	26 kDa		4	4
643	☑	☆	WGR domain containing protein [Tetrahymena thermophila]	gi|146185297	69 kDa		10	4
644	☑	☆	hypothetical protein TTHERM_00522810 [Tetrahymena thermophila]	gi|229595895	28 kDa		5	4
645	☑	☆	UMP-CMP kinase family protein [Tetrahymena thermophila]	gi|118362049	22 kDa	★		4
646	☑	☆	ubiquitin-transferase, HECT-domain [Tetrahymena thermophila]	gi|118398361	432 kDa		3	4
647	☑	☆	hypothetical protein TTHERM_00998940 [Tetrahymena thermophila]	gi|118397141	24 kDa		7	4
648	☑	☆	Phosphoribulokinase / Uridine kinase family protein [Tetrahymena thermophila]	gi|146184709	55 kDa	★	3	4
649	☑	☆	hypothetical protein TTHERM_00402110 [Tetrahymena thermophila]	gi|118361712	57 kDa			4
650	☑	☆	hypothetical protein TTHERM_00449470 [Tetrahymena thermophila]	gi|146164537	33 kDa		4	4
651	☑	☆	hypothetical protein TTHERM_00189370 [Tetrahymena thermophila]	gi|118366795	215 kDa		11	4
652	☑	☆	hypothetical protein TTHERM_00149230 [Tetrahymena thermophila]	gi|146180829	68 kDa		4	4
653	☑	☆	hypothetical protein TTHERM_00408950 [Tetrahymena thermophila]	gi|118375204	29 kDa			4
654	☑	☆	PCI domain containing protein [Tetrahymena thermophila]	gi|146174383	45 kDa		12	4
655	☑	☆	hypothetical protein TTHERM_00579210 [Tetrahymena thermophila]	gi|118379512	226 kDa		11	4
656	☑	☆	ATPase, AAA family protein [Tetrahymena thermophila]	gi|118346257	84 kDa		9	4
657	☑	☆	hypothetical protein TTHERM_01394370 [Tetrahymena thermophila]	gi|118394522	59 kDa		8	4
658	☑	☆	hypothetical protein TTHERM_01015930 [Tetrahymena thermophila]	gi|146185031	57 kDa		7	4
659	☑	☆	C2 domain containing protein [Tetrahymena thermophila]	gi|118383367	66 kDa		7	4
660	☑	☆	hypothetical protein TTHERM_00794520 [Tetrahymena thermophila]	gi|118375574	31 kDa		7	4
661	☑	☆	hypothetical protein TTHERM_00582290 [Tetrahymena thermophila]	gi|118371538	23 kDa		6	4
662	☑	☆	hypothetical protein TTHERM_00156700 [Tetrahymena thermophila]	gi|146162555	35 kDa		6	4
663	☑	☆	SerH3 immobilization antigen, putative [Tetrahymena thermophila]	gi|118354547	41 kDa		5	4
664	☑	☆	Ubiquitin carboxyl-terminal hydrolase family protein [Tetrahymena thermophila]	gi|118398123	193 kDa		5	4
665	☑	☆	ankyrin repeat protein, putative [Tetrahymena thermophila]	gi|118346833	23 kDa		5	4
666	☑	☆	hypothetical protein TTHERM_00464930 [Tetrahymena thermophila]	gi|146182663	23 kDa		4	4

Probability Legend

- over 95%
- 80% to 94%
- 50% to 79%
- 20% to 49%
- 0% to 19%

#	Visible?	Starred?	Bio View: Identified Proteins (1837)	Accession Number	Molecular Weight	Protein Grouping Ambiguity	01 Control	02 Sample
667	☑	☆	hypothetical protein TTHERM_00927010 [Tetrahymena thermophila]	gi|229596073	17 kDa		4	4
668	☑	☆	hypothetical protein TTHERM_00502720 [Tetrahymena thermophila]	gi|118378300	52 kDa		4	4
669	☑	☆	Ras family protein [Tetrahymena thermophila]	gi|229593818	25 kDa		4	4
670	☑	☆	hypothetical protein TTHERM_00594190 [Tetrahymena thermophila]	gi|118356851	32 kDa		4	4
671	☑	☆	hypothetical protein TTHERM_00823660 [Tetrahymena thermophila]	gi|118398278	21 kDa		3	4
672	☑	☆	Domain found in IF2B/IF5 family protein [Tetrahymena thermophila]	gi|146180835	45 kDa		3	4
673	☑	☆	hypothetical protein TTHERM_00532490 [Tetrahymena thermophila]	gi|118382425	20 kDa		3	4
674	☑	☆	NAC domain containing protein [Tetrahymena thermophila]	gi|146163174	17 kDa		2	4
675	☑	☆	N2,N2-dimethylguanosine tRNA methyltransferase family protein [Tetrahymena thermophila]	gi|118400048	64 kDa			4
676	☑	☆	isocitrate dehydrogenase, NADP-dependent family protein [Tetrahymena thermophila]	gi|118368129	49 kDa			4
677	☑	☆	Leucine Rich Repeat family protein [Tetrahymena thermophila]	gi|118370115	51 kDa			4
678	☑	☆	hypothetical protein TTHERM_00137810 [Tetrahymena thermophila]	gi|118373138	159 kDa			4
679	☑	☆	cyclic nucleotide-binding domain containing protein [Tetrahymena thermophila]	gi|118387586	230 kDa		10	4
680	☑	☆	hypothetical protein TTHERM_00727790 [Tetrahymena thermophila]	gi|118378991	71 kDa		4	4
681	☑	☆	hypothetical protein TTHERM_00048950 [Tetrahymena thermophila]	gi|118363118	120 kDa		5	4
682	☑	☆	hypothetical protein TTHERM_00326790 [Tetrahymena thermophila]	gi|118386649	50 kDa		5	4
683	☑	☆	hypothetical protein TTHERM_00497570 [Tetrahymena thermophila]	gi|118389800	15 kDa		3	4
684	☑	☆	Phosphofructokinase family protein [Tetrahymena thermophila]	gi|146170622	63 kDa		4	4
685	☑	☆	hypothetical protein TTHERM_00842530 [Tetrahymena thermophila]	gi|146183890	70 kDa		2	4
686	☑	☆	vacuolar ATPase subunit E [Tetrahymena thermophila]	gi|146175044	30 kDa		2	4
687	☑	☆	DNA-directed RNA polymerase, beta subunit family protein [Tetrahymena thermophila]	gi|118396434	134 kDa			4
688	☑	☆	hypothetical protein TTHERM_00293380 [Tetrahymena thermophila]	gi|229596125	69 kDa			4
689	☑	☆	U1 small nuclear ribonucleoprotein 70 kDa [Tetrahymena thermophila]	gi|118356855	35 kDa		4	4
690	☑	☆	Ras-related protein Rab11, putative [Tetrahymena thermophila]	gi|146180823	23 kDa	★	6	4
691	☑	☆	hypothetical protein TTHERM_00144900 [Tetrahymena thermophila]	gi|229596395	50 kDa		9	4
692	☑	☆	polyadenylate-binding protein, putative [Tetrahymena thermophila]	gi|118395954	28 kDa		3	4
693	☑	☆	Protein kinase domain containing protein [Tetrahymena thermophila]	gi|118367507	71 kDa		15	4
694	☑	☆	IMP dehydrogenase / GMP reductase domain containing protein [Tetrahymena thermophila]	gi|118401144	67 kDa		2	4
695	☑	☆	Protein kinase domain containing protein [Tetrahymena thermophila]	gi|118396825	54 kDa		8	4
696	☑	☆	hypothetical protein TTHERM_00075810 [Tetrahymena thermophila]	gi|146165686	52 kDa		4	4
697	☑	☆	Ubiquitin carboxyl-terminal hydrolase family protein [Tetrahymena thermophila]	gi|118362868	150 kDa			4
698	☑	☆	NADH-ubiquinone oxidoreductase 23 kDa subunit, mitochondrial precursor, putative [Tetrahymena thermophila]	gi|118359716	38 kDa		6	4
699	☑	☆	SPFH domain / Band 7 family protein [Tetrahymena thermophila]	gi|118389838	43 kDa		2	4
700	☑	☆	hypothetical protein TTHERM_00313610 [Tetrahymena thermophila]	gi|229594472	15 kDa			4
701	☑	☆	hypothetical protein TTHERM_00945210 [Tetrahymena thermophila]	gi|229593828	27 kDa		3	4
702	☑	☆	Adaptin N-terminal region family protein [Tetrahymena thermophila]	gi|118376222	105 kDa		2	4
703	☑	☆	hypothetical protein TTHERM_00442960 [Tetrahymena thermophila]	gi|118401778	52 kDa		5	4

Probability Legend: over 95% | 80% to 94% | 50% to 79% | 20% to 49% | 0% to 19%

#	Visible?	Starred?	Bio View: Identified Proteins (1837)	Accession Number	Molecular Weight	Protein Grouping Ambiguity	01 Control	02 Sample
704	☑	☆	Adaptin N-terminal region family protein [Tetrahymena thermophila]	gi|118399217	96 kDa		4	4
705	☑	☆	hypothetical protein TTHERM_00585230 [Tetrahymena thermophila]	gi|118400612	132 kDa			4
706	☑	☆	hypothetical protein TTHERM_00561290 [Tetrahymena thermophila]	gi|118353836	43 kDa		3	4
707	☑	☆	hypothetical protein TTHERM_00047490 [Tetrahymena thermophila]	gi|146165295	58 kDa		12	4
708	☑	☆	hypothetical protein IMG5_178920 [Ichthyophthirius multifiliis]	gi|340501537	29 kDa		3	4
709	☑	☆	valyl-tRNA synthetase family protein [Tetrahymena thermophila]	gi|146186117	119 kDa			4
710	☑	☆	hypothetical protein TTHERM_01164140 [Tetrahymena thermophila]	gi|118395414	171 kDa		27	4
711	☑	☆	3'5'-cyclic nucleotide phosphodiesterase family protein [Tetrahymena thermophila]	gi|118366045	61 kDa			4
712	☑	☆	Radial spoke protein 3 containing protein [Tetrahymena thermophila]	gi|146181083	80 kDa		10	4
713	☑	☆	AMP-binding enzyme family protein [Tetrahymena thermophila]	gi|118352716	76 kDa	★	17	4
714	☑	☆	hypothetical protein TTHERM_00113310 [Tetrahymena thermophila]	gi|229595750	33 kDa		7	4
715	☑	☆	hypothetical protein TTHERM_00599980 [Tetrahymena thermophila]	gi|118400299	53 kDa		7	4
716	☑	☆	hypothetical protein TTHERM_00049060 [Tetrahymena thermophila]	gi|118363278	59 kDa		2	4
717	☑	☆	Protein kinase domain containing protein [Tetrahymena thermophila]	gi|118377227	64 kDa		6	4
718	☑	☆	SRP54-type protein, GTPase domain containing protein [Tetrahymena thermophila]	gi|118351025	70 kDa		7	4
719	☑	☆	hypothetical protein TTHERM_00703380 [Tetrahymena thermophila]	gi|118399368	120 kDa		3	4
720	☑	☆	thioesterase family protein [Tetrahymena thermophila]	gi|118389282	101 kDa			4
721	☑	☆	hypothetical protein TTHERM_00494830 [Tetrahymena thermophila]	gi|118380203	46 kDa		4	4
722	☑	☆	ZIP Zinc transporter family protein [Tetrahymena thermophila]	gi|118396391	61 kDa		3	4
723	☑	☆	SNF2 family N-terminal domain containing protein [Tetrahymena thermophila]	gi|146165350	190 kDa		8	4
724	☑	☆	hypothetical protein TTHERM_00245360 [Tetrahymena thermophila]	gi|118381208	117 kDa		9	4
725	☑	☆	malate dehydrogenase family protein [Tetrahymena thermophila]	gi|118346673	38 kDa		5	4
726	☑	☆	hypothetical protein TTHERM_00569060 [Tetrahymena thermophila]	gi|118389040	113 kDa		18	4
727	☑	☆	Protein kinase domain containing protein [Tetrahymena thermophila]	gi|146161904	44 kDa	★	10	4
728	☑	☆	hypothetical protein TTHERM_00142350 [Tetrahymena thermophila]	gi|118355628	231 kDa			4
729	☑	☆	Glutathione S-transferase, N-terminal domain containing protein [Tetrahymena thermophila]	gi|118373873	25 kDa		3	4
730	☑	☆	Peptidase family M1 containing protein [Tetrahymena thermophila]	gi|146181543	106 kDa			4
731	☑	☆	V-type ATPase 116kDa subunit family protein [Tetrahymena thermophila]	gi|118371279	100 kDa		10	4
732	☑	☆	Cytochrome P450 family protein [Tetrahymena thermophila]	gi|146171206 (+1)	267 kDa		4	4
733	☑	☆	Radial spoke head containing protein [Tetrahymena thermophila]	gi|118361215 (+1)	58 kDa		6	4
734	☑	☆	hypothetical protein TTHERM_00637380 [Tetrahymena thermophila]	gi|229594828	44 kDa		5	4
735	☑	☆	MIZ zinc finger family protein [Tetrahymena thermophila]	gi|118401640	164 kDa		8	4
736	☑	☆	hypothetical protein TTHERM_00261950 [Tetrahymena thermophila]	gi|118351536	139 kDa		3	4
737	☑	☆	hypothetical protein TTHERM_00831530 [Tetrahymena thermophila]	gi|118360854	75 kDa	★	6	4
738	☑	☆	MCM2/3/5 family protein [Tetrahymena thermophila]	gi|118369821	90 kDa	★	22	4
739	☑	☆	60s Acidic ribosomal protein [Tetrahymena thermophila]	gi|229594362	11 kDa		3	4
740	☑	☆	hypothetical protein TTHERM_00578940 [Tetrahymena thermophila]	gi|146181496	90 kDa		7	4

Probability Legend

- over 95%
- 80% to 94%
- 50% to 79%
- 20% to 49%
- 0% to 19%

#	Visible?	Starred?	Bio View: Identified Proteins (1837)	Accession Number	Molecular Weight	Protein Grouping Ambiguity	01 Control	02 Sample
741	☑	☆	hypothetical protein TTHERM_00649080 [Tetrahymena thermophila]	gi|229595595	17 kDa		3	4
742	☑	☆	Peptidase family M1 containing protein [Tetrahymena thermophila]	gi|118381659	107 kDa			4
743	☑	☆	hypothetical protein TTHERM_00723220 [Tetrahymena thermophila]	gi|118398947	171 kDa	★	5	4
744	☑	☆	HMG box family protein [Tetrahymena thermophila]	gi|118401491 (+1)	71 kDa		4	4
745	☑	☆	Eukaryotic translation initiation factor 2 alpha subunit family protein [Tetrahymena thermophila]	gi|118352055	37 kDa		5	4
746	☑	☆	GTP1/OBG family protein [Tetrahymena thermophila]	gi|146183354	42 kDa			4
747	☑	☆	HEAT repeat family protein [Tetrahymena thermophila]	gi|146175736	93 kDa		2	4
748	☑	☆	hypothetical protein TTHERM_00444500 [Tetrahymena thermophila]	gi|118380296	289 kDa		15	4
749	☑	☆	Dynein heavy chain family protein [Tetrahymena thermophila]	gi|118376063	512 kDa	★	19	4
750	☑	☆	hypothetical protein TTHERM_00030260 [Tetrahymena thermophila]	gi|118346191	75 kDa			4
751	☑	☆	kinase domain containing protein [Tetrahymena thermophila]	gi|118395318	49 kDa		7	4
752	☑	☆	fatty-acid amide hydrolase [Tetrahymena thermophila]	gi|146161510	72 kDa		9	4
753	☑	☆	hypothetical protein TTHERM_00697370 [Tetrahymena thermophila]	gi|118385011	29 kDa		4	4
754	☑	☆	La domain containing protein [Tetrahymena thermophila]	gi|146174241	39 kDa			4
755	☑	☆	hypothetical protein TTHERM_00196190 [Tetrahymena thermophila]	gi|146169768	197 kDa		7	4
756	☑	☆	Glutathionylspermidine synthase family protein [Tetrahymena thermophila]	gi|118348648	70 kDa			4
757	☑	☆	ribosomal protein L27 containing protein [Tetrahymena thermophila]	gi|118401851	39 kDa			4
758	☑	☆	ribosomal protein L20 containing protein [Tetrahymena thermophila]	gi|118358435	20 kDa			4
759	☑	☆	MBOAT family protein [Tetrahymena thermophila]	gi|118362922	70 kDa		5	4
760	☑	☆	BNR/Asp-box repeat family protein [Tetrahymena thermophila]	gi|118383762	106 kDa		13	4
761	☑	☆	hypothetical protein TTHERM_00853090 [Tetrahymena thermophila]	gi|229593874	74 kDa			4
762	☑	☆	IQ calmodulin-binding motif family protein [Tetrahymena thermophila]	gi|118354193	87 kDa		5	3
763	☑	☆	hypothetical protein TTHERM_00101160 [Tetrahymena thermophila]	gi|118357834 (+1)	218 kDa		23	3
764	☑	☆	Ribosomal protein L44 containing protein [Tetrahymena thermophila]	gi|118353575	13 kDa		4	3
765	☑	☆	hypothetical protein TTHERM_01100380 [Tetrahymena thermophila]	gi|118395897	58 kDa		12	3
766	☑	☆	ABC transporter family protein [Tetrahymena thermophila]	gi|118372126	69 kDa			3
767	☑	☆	ribosomal protein L22 containing protein [Tetrahymena thermophila]	gi|146170683 (+1)	52 kDa		3	3
768	☑	☆	60S ribosomal protein L34, putative [Tetrahymena thermophila]	gi|118377144	14 kDa		3	3
769	☑	☆	hypothetical protein TTHERM_00372430 [Tetrahymena thermophila]	gi|118352600	30 kDa		8	3
770	☑	☆	ubiquitin transferase, HECT domain family protein [Tetrahymena thermophila]	gi|118353277	480 kDa	★	3	3
771	☑	☆	hypothetical protein TTHERM_00263310 [Tetrahymena thermophila]	gi|229596479	34 kDa		3	3
772	☑	☆	hypothetical protein TTHERM_00316290 [Tetrahymena thermophila]	gi|146180703	36 kDa		2	3
773	☑	☆	hypothetical protein TTHERM_01043290 [Tetrahymena thermophila]	gi|146185001	15 kDa		2	3
774	☑	☆	hypothetical protein TTHERM_00481330 [Tetrahymena thermophila]	gi|118368475	24 kDa			3
775	☑	☆	hypothetical protein TTHERM_00180960 [Tetrahymena thermophila]	gi|118350969	129 kDa		4	3
776	☑	☆	hypothetical protein TTHERM_00666370 [Tetrahymena thermophila]	gi|118373626	16 kDa		2	3
777	☑	☆	hypothetical protein TTHERM_00220590 [Tetrahymena thermophila]	gi|146179610	37 kDa			3

Probability Legend

- over 95%
- 80% to 94%
- 50% to 79%
- 20% to 49%
- 0% to 19%

#	Visible?	Starred?	Bio View: Identified Proteins (1837)	Accession Number	Molecular Weight	Protein Grouping Ambiguity	01 Control	02 Sample
778	☑	☆	Profilin family protein [Tetrahymena thermophila]	gi|118358090	17 kDa			3
779	☑	☆	hypothetical protein TTHERM_00289380 [Tetrahymena thermophila]	gi|118370910	14 kDa		3	3
780	☑	☆	hypothetical protein TTHERM_00753340 [Tetrahymena thermophila]	gi|118370263	23 kDa		3	3
781	☑	☆	EF hand family protein [Tetrahymena thermophila]	gi|229594649	85 kDa		6	3
782	☑	☆	3'5'-cyclic nucleotide phosphodiesterase family protein [Tetrahymena thermophila]	gi|146164481	110 kDa		2	3
783	☑	☆	hypothetical protein TTHERM_00529760 [Tetrahymena thermophila]	gi|146185889	24 kDa		3	3
784	☑	☆	DnaJ C-terminal region family protein [Tetrahymena thermophila]	gi|146186094	38 kDa		5	3
785	☑	☆	SNF2 family N-terminal domain containing protein [Tetrahymena thermophila]	gi|146174330	120 kDa			3
786	☑	☆	phospholipid-translocating P-type ATPase, flippase family protein [Tetrahymena thermophila]	gi|118352606	123 kDa		11	3
787	☑	☆	MutS domain III family protein [Tetrahymena thermophila]	gi|146164189	93 kDa		4	3
788	☑	☆	F-actin capping protein alpha subunit containing protein [Tetrahymena thermophila]	gi|146163066	95 kDa			3
789	☑	☆	cytochrome c oxidase subunit 1 [Tetrahymena malaccensis]	gi|114329850 (+1)	80 kDa		2	3
790	☑	☆	hypothetical protein TTHERM_00675650 [Tetrahymena thermophila]	gi|118363625	36 kDa		3	3
791	☑	☆	hypothetical protein TTHERM_00502359 [Tetrahymena thermophila]	gi|146181182	30 kDa		2	3
792	☑	☆	hypothetical protein TTHERM_01244570 [Tetrahymena thermophila]	gi|146181377	34 kDa		2	3
793	☑	☆	Core histone H2A/H2B/H3/H4 family protein [Tetrahymena thermophila]	gi|229596389 (+2)	15 kDa	★	3	3
794	☑	☆	IBR domain containing protein [Tetrahymena thermophila]	gi|118365058	58 kDa			3
795	☑	☆	ubiquinone/menaquinone biosynthesis methyltransferases family protein [Tetrahymena thermophila]	gi|118372904	202 kDa		10	3
796	☑	☆	hypothetical protein TTHERM_00494580 [Tetrahymena thermophila]	gi|146181675	32 kDa		4	3
797	☑	☆	Protein kinase domain containing protein [Tetrahymena thermophila]	gi|118381854	63 kDa	★		3
798	☑	☆	conserved hypothetical protein [Tetrahymena thermophila]	gi|146170421	68 kDa		10	3
799	☑	☆	hypothetical protein TTHERM_00636870 [Tetrahymena thermophila]	gi|146185700	28 kDa			3
800	☑	☆	EF hand family protein [Tetrahymena thermophila]	gi|118380372	19 kDa		6	3
801	☑	☆	EF hand family protein [Tetrahymena thermophila]	gi|118355698	73 kDa		7	3
802	☑	☆	hypothetical protein TTHERM_00219250 [Tetrahymena thermophila]	gi|146179448	30 kDa		4	3
803	☑	☆	BNR/Asp-box repeat family protein [Tetrahymena thermophila]	gi|146186284	103 kDa		8	3
804	☑	☆	AFR650Wp, putative [Tetrahymena thermophila]	gi|146165282	30 kDa		2	3
805	☑	☆	hypothetical protein TTHERM_00194800 [Tetrahymena thermophila]	gi|229595479	45 kDa		5	3
806	☑	☆	enoyl-CoA hydratase/isomerase family protein [Tetrahymena thermophila]	gi|146185338	31 kDa			3
807	☑	☆	hypothetical protein TTHERM_00046750 [Tetrahymena thermophila]	gi|118363254	129 kDa		4	3
808	☑	☆	hypothetical protein TTHERM_00149260 [Tetrahymena thermophila]	gi|146180831	33 kDa		3	3
809	☑	☆	hypothetical protein TTHERM_00426240 [Tetrahymena thermophila]	gi|146164670	16 kDa		2	3
810	☑	☆	hypothetical protein TTHERM_00394570 [Tetrahymena thermophila]	gi|146163614	37 kDa		6	3
811	☑	☆	Protein-tyrosine phosphatase containing protein [Tetrahymena thermophila]	gi|118356199	48 kDa			3
812	☑	☆	DnaJ domain containing protein [Tetrahymena thermophila]	gi|118361157	30 kDa		7	3
813	☑	☆	RecName: Full=60S ribosomal protein L27	gi|353678075	17 kDa		3	3
814	☑	☆	hypothetical protein TTHERM_00637590 [Tetrahymena thermophila]	gi|146185744	17 kDa		3	3

Probability Legend
- over 95%
- 80% to 94%
- 50% to 79%
- 20% to 49%
- 0% to 19%

#	Visible?	Starred?	Bio View: Identified Proteins (1837)	Accession Number	Molecular Weight	Protein Grouping Ambiguity	01 Control	02 Sample
815	☑	☆	hypothetical protein TTHERM_00583460 [Tetrahymena thermophila]	gi|118371574	354 kDa		20	3
816	☑	☆	Zinc finger, C2H2 type family protein [Tetrahymena thermophila]	gi|118359832	452 kDa		10	3
817	☑	☆	Leucine Rich Repeat family protein [Tetrahymena thermophila]	gi|118368582	23 kDa		8	3
818	☑	☆	hypothetical protein TTHERM_00572190 [Tetrahymena thermophila]	gi|146184078	70 kDa		8	3
819	☑	☆	hypothetical protein TTHERM_00485750 [Tetrahymena thermophila]	gi|118400934	24 kDa		6	3
820	☑	☆	Ser/Thr protein phosphatase family protein [Tetrahymena thermophila]	gi|118394669	36 kDa	★	6	3
821	☑	☆	conserved hypothetical protein [Tetrahymena thermophila]	gi|146181517	126 kDa		6	3
822	☑	☆	hypothetical protein TTHERM_00890150 [Tetrahymena thermophila]	gi|118374232	33 kDa		6	3
823	☑	☆	Protein kinase domain containing protein [Tetrahymena thermophila]	gi|118362502	69 kDa		6	3
824	☑	☆	calcium-translocating P-type ATPase, PMCA-type family protein [Tetrahymena thermophila]	gi|118398887	102 kDa	★	5	3
825	☑	☆	hypothetical protein TTHERM_00188950 [Tetrahymena thermophila]	gi|118366711	57 kDa		5	3
826	☑	☆	hypothetical protein TTHERM_00185560 [Tetrahymena thermophila]	gi|229596559	27 kDa		5	3
827	☑	☆	hypothetical protein TTHERM_00498150 [Tetrahymena thermophila]	gi|118389916	62 kDa		5	3
828	☑	☆	small GTP-binding protein domain containing protein [Tetrahymena thermophila]	gi|118372074 (+1)	22 kDa		5	3
829	☑	☆	Alcohol dehydrogenase I, putative [Tetrahymena thermophila]	gi|146163286	40 kDa		4	3
830	☑	☆	hypothetical protein TTHERM_00829380 [Tetrahymena thermophila]	gi|118360820	20 kDa		4	3
831	☑	☆	Eukaryotic porin family protein [Tetrahymena thermophila]	gi|118349153	48 kDa		4	3
832	☑	☆	glycosyl transferase, group 2 family protein [Tetrahymena thermophila]	gi|146161605	27 kDa		4	3
833	☑	☆	EF-1 guanine nucleotide exchange domain containing protein [Tetrahymena thermophila]	gi|118377576	25 kDa		4	3
834	☑	☆	Cytochrome b5-like Heme/Steroid binding domain containing protein [Tetrahymena thermophila]	gi|118401913	24 kDa		4	3
835	☑	☆	conserved hypothetical protein [Tetrahymena thermophila]	gi|118395316	19 kDa		4	3
836	☑	☆	SPFH domain / Band 7 family protein [Tetrahymena thermophila]	gi|118366869	35 kDa		3	3
837	☑	☆	hypothetical protein TTHERM_00418110 [Tetrahymena thermophila]	gi|229596729 (+1)	19 kDa		3	3
838	☑	☆	hypothetical protein TTHERM_00429880 [Tetrahymena thermophila]	gi|146163373	28 kDa		3	3
839	☑	☆	hypothetical protein TTHERM_00541460 [Tetrahymena thermophila]	gi|146161176	25 kDa		3	3
840	☑	☆	ribosomal protein S2 containing protein [Tetrahymena thermophila]	gi|146165118	27 kDa		3	3
841	☑	☆	hypothetical protein TTHERM_00794470 [Tetrahymena thermophila]	gi|118375564	19 kDa		3	3
842	☑	☆	emp24/gp25L/p24 family protein [Tetrahymena thermophila]	gi|118378176	23 kDa		2	3
843	☑	☆	hypothetical protein TTHERM_00773400 [Tetrahymena thermophila]	gi|118398530	116 kDa		2	3
844	☑	☆	hypothetical protein TTHERM_00444800 [Tetrahymena thermophila]	gi|146181751	17 kDa		2	3
845	☑	☆	hypothetical protein TTHERM_01347920 [Tetrahymena thermophila]	gi|118394635	22 kDa		2	3
846	☑	☆	TPR Domain containing protein [Tetrahymena thermophila]	gi|118389702	110 kDa		2	3
847	☑	☆	hypothetical protein TTHERM_00621310 [Tetrahymena thermophila]	gi|118369504	112 kDa		2	3
848	☑	☆	hypothetical protein TTHERM_00614680 [Tetrahymena thermophila]	gi|118382963	46 kDa		2	3
849	☑	☆	SCO1/SenC family protein [Tetrahymena thermophila]	gi|118378359	38 kDa			3
850	☑	☆	hypothetical protein TTHERM_00558550 [Tetrahymena thermophila]	gi|118378483	37 kDa			3
851	☑	☆	hypothetical protein TTHERM_00984990 [Tetrahymena thermophila]	gi|146163651	38 kDa			3

Probability Legend

- over 95%
- 80% to 94%
- 50% to 79%
- 20% to 49%
- 0% to 19%

#	Visible?	Starred?	Bio View: Identified Proteins (1837)	Accession Number	Molecular Weight	Protein Grouping Ambiguity	01 Control	02 Sample
852	☑	☆	aconitate hydratase [Tetrahymena thermophila]	gi|118367081	99 kDa			3
853	☑	☆	DEAD/DEAH box helicase family protein [Tetrahymena thermophila]	gi|118389642	73 kDa			3
854	☑	☆	Glucosamine-6-phosphate isomerase/6-phosphogluconolactonase family protein [Tetrahymena thermophila]	gi|146181962	90 kDa			3
855	☑	☆	Acyl-CoA oxidase family protein [Tetrahymena thermophila]	gi|118352470	76 kDa			3
856	☑	☆	hypothetical protein TTHERM_00322930 [Tetrahymena thermophila]	gi|146164100	75 kDa		4	3
857	☑	☆	Adenylate kinase family protein [Tetrahymena thermophila]	gi|118376658	241 kDa		8	3
858	☑	☆	Elongation factor Tu C-terminal domain containing protein [Tetrahymena thermophila]	gi|146163353	74 kDa			3
859	☑	☆	Mitochondrial carrier protein [Tetrahymena thermophila]	gi|118369562	36 kDa		3	3
860	☑	☆	hypothetical protein TTHERM_00681790 [Tetrahymena thermophila]	gi|118388436	39 kDa			3
861	☑	☆	hypothetical protein TTHERM_01197030 [Tetrahymena thermophila]	gi|118374158	86 kDa			3
862	☑	☆	hypothetical protein TTHERM_00456790 [Tetrahymena thermophila]	gi|118382053	14 kDa		3	3
863	☑	☆	NADH-quinone oxidoreductase, E subunit family protein [Tetrahymena thermophila]	gi|118368650	31 kDa		5	3
864	☑	☆	hypothetical protein TTHERM_00123680 [Tetrahymena thermophila]	gi|118355204	36 kDa		7	3
865	☑	☆	PA domain containing protein [Tetrahymena thermophila]	gi|118348244	58 kDa		13	3
866	☑	☆	Protein kinase domain containing protein [Tetrahymena thermophila]	gi|118380529	62 kDa		5	3
867	☑	☆	heterochromatin-associated protein 1-like protein [Tetrahymena thermophila]	gi|146163183 (+1)	28 kDa		8	3
868	☑	☆	NAC domain containing protein [Tetrahymena thermophila]	gi|146172717	21 kDa		2	3
869	☑	☆	AT hook motif family protein [Tetrahymena thermophila]	gi|118389557	209 kDa		9	3
870	☑	☆	ribosomal protein S15 containing protein [Tetrahymena thermophila]	gi|146162564	23 kDa		2	3
871	☑	☆	hypothetical protein TTHERM_00797990 [Tetrahymena thermophila]	gi|118374387	99 kDa			3
872	☑	☆	hypothetical protein TTHERM_01093530 [Tetrahymena thermophila]	gi|118396013	164 kDa			3
873	☑	☆	Ribosomal protein L7Ae containing protein [Tetrahymena thermophila]	gi|118358524	15 kDa		3	3
874	☑	☆	oxidoreductase, short chain dehydrogenase/reductase family protein [Tetrahymena thermophila]	gi|118346627	34 kDa		4	3
875	☑	☆	hypothetical protein TTHERM_00590080 [Tetrahymena thermophila]	gi|146176354	83 kDa		3	3
876	☑	☆	DNA-directed RNA polymerase, beta subunit family protein [Tetrahymena thermophila]	gi|118358942	130 kDa			3
877	☑	☆	Protein prenyltransferase alpha subunit repeat containing protein [Tetrahymena thermophila]	gi|118368626	66 kDa			3
878	☑	☆	Mitochondrial carrier protein [Tetrahymena thermophila]	gi|118374989	33 kDa		3	3
879	☑	☆	DnaJ domain containing protein [Tetrahymena thermophila]	gi|118357868	42 kDa		4	3
880	☑	☆	hypothetical protein TTHERM_00660330 [Tetrahymena thermophila]	gi|118373807	89 kDa		6	3
881	☑	☆	Protein kinase domain containing protein [Tetrahymena thermophila]	gi|118361427	122 kDa		8	3
882	☑	☆	nucleosome assembly protein (NAP) [Tetrahymena thermophila]	gi|146181097	40 kDa		3	3
883	☑	☆	nuclear cap binding protein [Tetrahymena thermophila]	gi|118401473	28 kDa			3
884	☑	☆	hypothetical protein TTHERM_00721540 [Tetrahymena thermophila]	gi|118399120	115 kDa			3
885	☑	☆	cytidyltransferase-related domain containing protein [Tetrahymena thermophila]	gi|118350194	52 kDa	★	3	3
886	☑	☆	SPFH domain / Band 7 family protein [Tetrahymena thermophila]	gi|146174422	31 kDa		2	3
887	☑	☆	TPR Domain containing protein [Tetrahymena thermophila]	gi|146179561	154 kDa		2	3
888	☑	☆	hypothetical protein TTHERM_00083370 [Tetrahymena thermophila]	gi|118358684	48 kDa		2	3

Probability Legend
- over 95%
- 80% to 94%
- 50% to 79%
- 20% to 49%
- 0% to 19%

#	Visible?	Starred?	Bio View: Identified Proteins (1837)	Accession Number	Molecular Weight	Protein Grouping Ambiguity	01 Control	02 Sample
889	☑	☆	S-adenosylmethionine synthetase family protein [Tetrahymena thermophila]	gi|118380290	44 kDa		4	3
890	☑	☆	hypothetical protein TTHERM_01205260 [Tetrahymena thermophila]	gi|118395201	114 kDa		8	3
891	☑	☆	actin related protein [Tetrahymena thermophila]	gi|118349009	117 kDa			3
892	☑	☆	hypothetical protein TTHERM_00221170 [Tetrahymena thermophila]	gi|146179882	15 kDa			3
893	☑	☆	MIF4G domain containing protein [Tetrahymena thermophila]	gi|118401738	119 kDa	★	2	3
894	☑	☆	hypothetical protein TTHERM_00780500 [Tetrahymena thermophila]	gi|146183466	38 kDa		2	3
895	☑	☆	hypothetical protein TTHERM_00680670 [Tetrahymena thermophila]	gi|146183901	10 kDa		2	3
896	☑	☆	hypothetical protein TTHERM_00985010 [Tetrahymena thermophila]	gi|146163653	23 kDa		4	3
897	☑	☆	Protein kinase domain containing protein [Tetrahymena thermophila]	gi|118347348	65 kDa		2	3
898	☑	☆	hydrolase, alpha/beta fold family protein [Tetrahymena thermophila]	gi|118375554	153 kDa		9	3
899	☑	☆	hypothetical protein TTHERM_00637600 [Tetrahymena thermophila]	gi|118400196	18 kDa			3
900	☑	☆	2-methylcitrate synthase/citrate synthase II family protein [Tetrahymena thermophila]	gi|118380705	42 kDa		5	3
901	☑	☆	hypothetical protein TTHERM_00160560 [Tetrahymena thermophila]	gi|118353031	31 kDa		2	3
902	☑	☆	hypothetical protein TTHERM_00455630 [Tetrahymena thermophila]	gi|229594330	39 kDa			3
903	☑	☆	conserved hypothetical protein [Tetrahymena thermophila]	gi|118366801	96 kDa			3
904	☑	☆	conserved hypothetical protein [Tetrahymena thermophila]	gi|146162471	52 kDa		7	3
905	☑	☆	Clathrin adaptor complex small chain family protein [Tetrahymena thermophila]	gi|118385088	18 kDa		2	3
906	☑	☆	normocyte binding protein 2a, putative [Tetrahymena thermophila]	gi|118395486	21 kDa	★	5	3
907	☑	☆	hypothetical protein TTHERM_00218390 [Tetrahymena thermophila]	gi|118374647	697 kDa		9	3
908	☑	☆	conserved hypothetical protein [Tetrahymena thermophila]	gi|118382023	51 kDa			3
909	☑	☆	Vacuolar protein sorting-associated protein 26 containing protein [Tetrahymena thermophila]	gi|118395666	234 kDa			3
910	☑	☆	DEAD/DEAH box helicase family protein [Tetrahymena thermophila]	gi|118376256	120 kDa		9	3
911	☑	☆	hypothetical protein TTHERM_01285910 [Tetrahymena thermophila]	gi|146184652	53 kDa		8	3
912	☑	☆	Myb-like DNA-binding domain containing protein [Tetrahymena thermophila]	gi|229593594	188 kDa			3
913	☑	☆	NOL1/NOP2/sun family protein [Tetrahymena thermophila]	gi|118384004	133 kDa			3
914	☑	☆	Cytosol aminopeptidase family, catalytic domain containing protein [Tetrahymena thermophila]	gi|118348238	55 kDa		2	3
915	☑	☆	hypothetical protein TTHERM_00013760 [Tetrahymena thermophila]	gi|118350148	108 kDa		3	3
916	☑	☆	TBC domain containing protein [Tetrahymena thermophila]	gi|118369560	240 kDa	★	3	3
917	☑	☆	cyclic nucleotide-binding domain containing protein [Tetrahymena thermophila]	gi|118349586	51 kDa			3
918	☑	☆	conserved hypothetical protein [Tetrahymena thermophila]	gi|118350903	68 kDa		6	3
919	☑	☆	NPL4 family protein [Tetrahymena thermophila]	gi|118348606	134 kDa			3
920	☑	☆	Ubiquitin carboxyl-terminal hydrolase family protein [Tetrahymena thermophila]	gi|118397798	303 kDa			3
921	☑	☆	eukaryotic translation initiation factor 2 gamma subunit [Tetrahymena thermophila]	gi|118372425	56 kDa		7	3
922	☑	☆	Ribosomal protein S26e containing protein [Tetrahymena thermophila]	gi|118371293 (+1)	23 kDa		3	3
923	☑	☆	hypothetical protein TTHERM_00220810 [Tetrahymena thermophila]	gi|118374931	161 kDa		4	3
924	☑	☆	ymf68 [Tetrahymena thermophila]	gi|15027666	73 kDa		2	3
925	☑	☆	Eukaryotic translation initiation factor 3 subunit, putative [Tetrahymena thermophila]	gi|146185812	50 kDa		6	3

Probability Legend

- over 95%
- 80% to 94%
- 50% to 79%
- 20% to 49%
- 0% to 19%

#	Visible?	Starred?	Bio View: Identified Proteins (1837)	Accession Number	Molecular Weight	Protein Grouping Ambiguity	01 Control	02 Sample
926			Viral A-type inclusion protein repeat containing protein [Tetrahymena thermophila]	gi|118365437	164 kDa		18	3
927			hypothetical protein TTHERM_00554590 [Tetrahymena thermophila]	gi|229596461	36 kDa		8	3
928			Zinc finger, C2H2 type family protein [Tetrahymena thermophila]	gi|146171720	101 kDa			3
929			hypothetical protein TTHERM_01242400 [Tetrahymena thermophila]	gi|118384656	15 kDa			3
930			hypothetical protein TTHERM_00486650 [Tetrahymena thermophila]	gi|118401112	35 kDa		4	3
931			hypothetical protein TTHERM_00723310 [Tetrahymena thermophila]	gi|118398965	32 kDa		3	3
932			hypothetical protein TTHERM_00444170 [Tetrahymena thermophila]	gi|118380230	36 kDa		5	3
933			hypothetical protein TTHERM_00105300 [Tetrahymena thermophila]	gi|118358064	157 kDa		8	3
934			hypothetical protein TTHERM_00290740 [Tetrahymena thermophila]	gi|118370982	78 kDa		11	3
935			hydroxyacylglutathione hydrolase, putative [Tetrahymena thermophila]	gi|146183629	35 kDa			3
936			hypothetical protein TTHERM_00301790 [Tetrahymena thermophila]	gi|146182442	46 kDa			3
937			Histidine acid phosphatase family protein [Tetrahymena thermophila]	gi|118354001	67 kDa		4	3
938			hypothetical protein TTHERM_00558350 [Tetrahymena thermophila]	gi|229594710	35 kDa		7	3
939			hypothetical protein TTHERM_01227760 [Tetrahymena thermophila]	gi|229596058	20 kDa		7	3
940			hypothetical protein TTHERM_00470740 [Tetrahymena thermophila]	gi|118401287	64 kDa		3	3
941			hypothetical protein TTHERM_00218340 [Tetrahymena thermophila]	gi|118374637	17 kDa		3	3
942			Probable 26S proteasome non-ATPase regulatory subunit 6, putative [Tetrahymena thermophila]	gi|118366971	37 kDa		9	3
943			Sec23/Sec24 trunk domain containing protein [Tetrahymena thermophila]	gi|118381595	86 kDa			3
944			TPR Domain containing protein [Tetrahymena thermophila]	gi|146184776	85 kDa		9	3
945			Ubiquitin-conjugating enzyme family protein [Tetrahymena thermophila]	gi|118382495	21 kDa		4	2
946			Mov34/MPN/PAD-1 family protein [Tetrahymena thermophila]	gi|118365100	276 kDa		4	2
947			TB2/DP1, HVA22 family protein [Tetrahymena thermophila]	gi|118349644	19 kDa		3	2
948			zinc finger protein [Tetrahymena thermophila]	gi|146185835	72 kDa		4	2
949			hypothetical protein TTHERM_00334340 [Tetrahymena thermophila]	gi|229595410	13 kDa		4	2
950			HMG box family protein [Tetrahymena thermophila]	gi|229595100	16 kDa			2
951			granule lattice protein 5 precursor, putative [Tetrahymena thermophila]	gi|229595645	42 kDa		7	2
952			hypothetical protein TTHERM_00185570 [Tetrahymena thermophila]	gi|118351099	25 kDa		3	2
953			hypothetical protein TTHERM_00499570 [Tetrahymena thermophila]	gi|118378078	28 kDa		8	2
954			Ribosomal protein L24e containing protein [Tetrahymena thermophila]	gi|118368331	18 kDa			2
955			Vacuolar ATP synthase, putative [Tetrahymena thermophila]	gi|146162671	53 kDa	★		2
956			Dynein heavy chain family protein [Tetrahymena thermophila]	gi|229595213	500 kDa	★	7	2
957			hypothetical protein TTHERM_00899550 [Tetrahymena thermophila]	gi|118377241	128 kDa		5	2
958			hypothetical protein TTHERM_01060800 [Tetrahymena thermophila]	gi|118345345	45 kDa		10	2
959			hypothetical protein TTHERM_00310880 [Tetrahymena thermophila]	gi|118375943	14 kDa		2	2
960			translation elongation factor G [Tetrahymena thermophila]	gi|118395236	85 kDa			2
961			hypothetical protein TTHERM_00470710 [Tetrahymena thermophila]	gi|118401281	15 kDa		2	2
962			hypothetical protein TTHERM_00191720 [Tetrahymena thermophila]	gi|229595569	44 kDa		7	2

Probability Legend

- over 95%
- 80% to 94%
- 50% to 79%
- 20% to 49%
- 0% to 19%

#	Visible?	Starred?	Bio View: Identified Proteins (1837)	Accession Number	Molecular Weight	Protein Grouping Ambiguity	01 Control	02 Sample
963	☑	☆	Sm protein [Tetrahymena thermophila]	gi|146182712	169 kDa		3	2
964	☑	☆	NADH dehydrogenase subunit 9 [Tetrahymena thermophila]	gi|15027658 (+1)	24 kDa		3	2
965	☑	☆	ribosomal protein L21 containing protein [Tetrahymena thermophila]	gi|118369903	34 kDa			2
966	☑	☆	DNA-directed RNA polymerase I 40 kDa polypeptide [Tetrahymena thermophila]	gi|146164033	39 kDa			2
967	☑	☆	signal recognition particle 54 kDa protein 1, SRP54 [Tetrahymena thermophila]	gi|118370390	57 kDa		5	2
968	☑	☆	hypothetical protein TTHERM_00122270 [Tetrahymena thermophila]	gi|118355124	153 kDa			2
969	☑	☆	protofilament ribbon protein, putative [Tetrahymena thermophila]	gi|146181346	30 kDa		2	2
970	☑	☆	HIT domain containing protein [Tetrahymena thermophila]	gi|146182779	13 kDa			2
971	☑	☆	hypothetical protein TTHERM_00161200 [Tetrahymena thermophila]	gi|146162663	10 kDa		4	2
972	☑	☆	EF hand family protein [Tetrahymena thermophila]	gi|118374745	48 kDa		5	2
973	☑	☆	centrin [Tetrahymena thermophila]	gi|146165152	19 kDa		9	2
974	☑	☆	L1P family of ribosomal proteins containing protein [Tetrahymena thermophila]	gi|146184970	30 kDa		3	2
975	☑	☆	Kinesin motor domain containing protein [Tetrahymena thermophila]	gi|118368453 (+1)	85 kDa		2	2
976	☑	☆	hypothetical protein TTHERM_00522970 [Tetrahymena thermophila]	gi|146165141	32 kDa		11	2
977	☑	☆	hypothetical protein TTHERM_00105150 [Tetrahymena thermophila]	gi|118358038	123 kDa		2	2
978	☑	☆	conserved hypothetical protein [Tetrahymena thermophila]	gi|146180883	73 kDa		2	2
979	☑	☆	CPSF A subunit region family protein [Tetrahymena thermophila]	gi|118400837	135 kDa		4	2
980	☑	☆	Ras family protein [Tetrahymena thermophila]	gi|229595003	24 kDa			2
981	☑	☆	hypothetical protein TTHERM_00630410 [Tetrahymena thermophila]	gi|118379196	84 kDa		3	2
982	☑	☆	V-type ATPase, F subunit family protein [Tetrahymena thermophila]	gi|118363020	22 kDa			2
983	☑	☆	hypothetical protein TTHERM_00441960 [Tetrahymena thermophila]	gi|118401578	68 kDa		2	2
984	☑	☆	14-3-3 protein [Tetrahymena thermophila]	gi|146162627	29 kDa	★	4	2
985	☑	☆	phosphoenolpyruvate carboxykinase [Tetrahymena thermophila]	gi|146186256	62 kDa			2
986	☑	☆	Integral membrane protein [Tetrahymena thermophila]	gi|118397586	31 kDa		2	2
987	☑	☆	hypothetical protein TTHERM_00967550 [Tetrahymena thermophila]	gi|118351337	72 kDa	★	4	2
988	☑	☆	hypothetical protein TTHERM_00127020 [Tetrahymena thermophila]	gi|118366127	123 kDa			2
989	☑	☆	hypothetical protein TTHERM_00442200 [Tetrahymena thermophila]	gi|118401626	32 kDa		2	2
990	☑	☆	preprotein translocase, SecY subunit containing protein [Tetrahymena thermophila]	gi|146161267	61 kDa		5	2
991	☑	☆	Ras family protein [Tetrahymena thermophila]	gi|118365218	25 kDa	★	6	2
992	☑	☆	ribosomal protein L15 containing protein [Tetrahymena thermophila]	gi|118373022	35 kDa			2
993	☑	☆	NLI interacting factor-like phosphatase family protein [Tetrahymena thermophila]	gi|118380137	38 kDa			2
994	☑	☆	hypothetical protein TTHERM_00151250 [Tetrahymena thermophila]	gi|146180888	16 kDa			2
995	☑	☆	hypothetical protein TTHERM_00529610 [Tetrahymena thermophila]	gi|118400701	51 kDa		4	2
996	☑	☆	hypothetical protein TTHERM_00682930 [Tetrahymena thermophila]	gi|146183912	8 kDa			2
997	☑	☆	Ubiquitin carboxyl-terminal hydrolase family protein [Tetrahymena thermophila]	gi|118385826	139 kDa			2
998	☑	☆	Riboflavin kinase / FAD synthetase family protein [Tetrahymena thermophila]	gi|146162111	31 kDa		3	2
999	☑	☆	hypothetical protein TTHERM_01084200 [Tetrahymena thermophila]	gi|146184913	89 kDa		8	2

Probability Legend
- over 95%
- 80% to 94%
- 50% to 79%
- 20% to 49%
- 0% to 19%

#	Visible?	Starred?	Bio View: Identified Proteins (1837)	Accession Number	Molecular Weight	Protein Grouping Ambiguity	01 Control	02 Sample
1000	☑	☆	hypothetical protein TTHERM_00494060 [Tetrahymena thermophila]	gi|118380069	28 kDa		3	2
1001	☑	☆	hypothetical protein TTHERM_00245120 [Tetrahymena thermophila]	gi|146181986	49 kDa			2
1002	☑	☆	hypothetical protein TTHERM_01092400 [Tetrahymena thermophila]	gi|118384606	209 kDa		16	2
1003	☑	☆	60s Acidic ribosomal protein [Tetrahymena thermophila]	gi|118384233	12 kDa		2	2
1004	☑	☆	BNR/Asp-box repeat family protein [Tetrahymena thermophila]	gi|229594938	103 kDa		10	2
1005	☑	☆	PBS lyase HEAT-like repeat family protein [Tetrahymena thermophila]	gi|118390247	136 kDa		8	2
1006	☑	☆	Oligosaccharyl transferase STT3 subunit family protein [Tetrahymena thermophila]	gi|118365664	91 kDa		8	2
1007	☑	☆	ABC transporter family protein [Tetrahymena thermophila]	gi|118385520	98 kDa		8	2
1008	☑	☆	AMP-binding enzyme family protein [Tetrahymena thermophila]	gi|118389456	74 kDa	★	8	2
1009	☑	☆	hypothetical protein TTHERM_00896100 [Tetrahymena thermophila]	gi|118397836	48 kDa		8	2
1010	☑	☆	NAD transhydrogenase beta subunit family protein [Tetrahymena thermophila]	gi|118376190	137 kDa		7	2
1011	☑	☆	Ras family protein [Tetrahymena thermophila]	gi|229594508	24 kDa	★	6	2
1012	☑	☆	Sm protein [Tetrahymena thermophila]	gi|118398961	18 kDa		6	2
1013	☑	☆	hypothetical protein TTHERM_00474520 [Tetrahymena thermophila]	gi|118381551	149 kDa		6	2
1014	☑	☆	hypothetical protein TTHERM_00522160 [Tetrahymena thermophila]	gi|146165094	27 kDa		5	2
1015	☑	☆	conserved hypothetical protein [Tetrahymena thermophila]	gi|146163315	53 kDa		5	2
1016	☑	☆	Cullin family protein [Tetrahymena thermophila]	gi|146176040	90 kDa		5	2
1017	☑	☆	Guanylate-binding protein, N-terminal domain containing protein [Tetrahymena thermophila]	gi|118358352	93 kDa		5	2
1018	☑	☆	hypothetical protein TTHERM_00196300 [Tetrahymena thermophila]	gi|118368149 (+1)	19 kDa		5	2
1019	☑	☆	Protein kinase domain containing protein [Tetrahymena thermophila]	gi|146165567	35 kDa		5	2
1020	☑	☆	IFT52/Intraflagellar transport protein 52 [Tetrahymena thermophila]	gi|146185658	70 kDa		4	2
1021	☑	☆	conserved hypothetical protein [Tetrahymena thermophila]	gi|118379382	76 kDa		4	2
1022	☑	☆	hypothetical protein TTHERM_00446290 [Tetrahymena thermophila]	gi|118380456	44 kDa		4	2
1023	☑	☆	EF hand family protein [Tetrahymena thermophila]	gi|229594529	129 kDa		4	2
1024	☑	☆	hypothetical protein TTHERM_00727840 [Tetrahymena thermophila]	gi|146181401	27 kDa		4	2
1025	☑	☆	Acyl-CoA oxidase family protein [Tetrahymena thermophila]	gi|118373365	82 kDa		4	2
1026	☑	☆	ZPR1 zinc-finger domain containing protein [Tetrahymena thermophila]	gi|146185455	55 kDa		4	2
1027	☑	☆	POLO box duplicated region family protein [Tetrahymena thermophila]	gi|118396387	65 kDa		4	2
1028	☑	☆	Ubiquitin interaction motif family protein [Tetrahymena thermophila]	gi|118401493	43 kDa		3	2
1029	☑	☆	hypothetical protein TTHERM_00695750 [Tetrahymena thermophila]	gi|118384895	15 kDa		3	2
1030	☑	☆	Gar1 protein RNA binding region containing protein [Tetrahymena thermophila]	gi|118385169	22 kDa		3	2
1031	☑	☆	TB2/DP1, HVA22 family protein [Tetrahymena thermophila]	gi|118358377	20 kDa		3	2
1032	☑	☆	hypothetical protein TTHERM_00628400 [Tetrahymena thermophila]	gi|229595150	28 kDa		3	2
1033	☑	☆	hypothetical protein TTHERM_00091770 [Tetrahymena thermophila]	gi|118359176	44 kDa	★	3	2
1034	☑	☆	Mitochondrial glycoprotein [Tetrahymena thermophila]	gi|118364930	29 kDa		3	2
1035	☑	☆	hypothetical protein TTHERM_00938940 [Tetrahymena thermophila]	gi|118397473	19 kDa		3	2
1036	☑	☆	Phosphoribulokinase / Uridine kinase family protein [Tetrahymena thermophila]	gi|118399267	36 kDa		3	2

Probability Legend
- over 95%
- 80% to 94%
- 50% to 79%
- 20% to 49%
- 0% to 19%

#	Visible?	Starred?	Bio View: Identified Proteins (1837)	Accession Number	Molecular Weight	Protein Grouping Ambiguity	01 Control	02 Sample
1037	☑	☆	outer dynein arm docking complex protein ODA1, putative [Tetrahymena thermophila]	gi|146163570	65 kDa		3	2
1038	☑	☆	conserved hypothetical protein [Tetrahymena thermophila]	gi|146176765	43 kDa		3	2
1039	☑	☆	hypothetical protein TTHERM_01394360 [Tetrahymena thermophila]	gi|118394520	25 kDa		3	2
1040	☑	☆	zinc finger protein [Tetrahymena thermophila]	gi|229594824	32 kDa		3	2
1041	☑	☆	Ubiquitin carboxyl-terminal hydrolase, family 1 protein [Tetrahymena thermophila]	gi|229594443	43 kDa		3	2
1042	☑	☆	beta tubulin [Dinophyceae sp. RS6]	gi|134142269	34 kDa	★	3	2
1043	☑	☆	Ras family protein [Tetrahymena thermophila]	gi|229596300	24 kDa		3	2
1044	☑	☆	hypothetical protein TTHERM_00927000 [Tetrahymena thermophila]	gi|118397554	24 kDa		3	2
1045	☑	☆	PX domain containing protein [Tetrahymena thermophila]	gi|118376858	91 kDa		2	2
1046	☑	☆	hypothetical protein TTHERM_00418090 [Tetrahymena thermophila]	gi|146161478	20 kDa		2	2
1047	☑	☆	hypothetical protein TTHERM_00037080 [Tetrahymena thermophila]	gi|118346463	81 kDa		2	2
1048	☑	☆	SPFH domain / Band 7 family protein [Tetrahymena thermophila]	gi|118401407	34 kDa		2	2
1049	☑	☆	hypothetical protein TTHERM_00193950 [Tetrahymena thermophila]	gi|118367877	22 kDa		2	2
1050	☑	☆	hypothetical protein TTHERM_00616320 [Tetrahymena thermophila]	gi|118383093	15 kDa		2	2
1051	☑	☆	hypothetical protein TTHERM_00805850 [Tetrahymena thermophila]	gi|118357470	14 kDa		2	2
1052	☑	☆	inorganic h+ pyrophosphatase, putative [Ichthyophthirius multifiliis]	gi|340503350	82 kDa	★	2	2
1053	☑	☆	PSP family protein [Tetrahymena thermophila]	gi|118350154	61 kDa		2	2
1054	☑	☆	hypothetical protein TTHERM_01481590 [Tetrahymena thermophila]	gi|118355291 (+3)	89 kDa		2	2
1055	☑	☆	hypothetical protein TTHERM_00143580 [Tetrahymena thermophila]	gi|118355676	18 kDa		2	2
1056	☑	☆	hypothetical protein TTHERM_00128870 [Tetrahymena thermophila]	gi|118366299	146 kDa		2	2
1057	☑	☆	hypothetical protein TTHERM_00459290 [Tetrahymena thermophila]	gi|118382155	22 kDa		2	2
1058	☑	☆	Translationally controlled tumor protein [Tetrahymena thermophila]	gi|118385474	19 kDa		2	2
1059	☑	☆	Spermine/spermidine synthase family protein [Tetrahymena thermophila]	gi|118388670	41 kDa		2	2
1060	☑	☆	Cytochrome oxidase assembly protein [Tetrahymena thermophila]	gi|118389636	51 kDa		2	2
1061	☑	☆	hypothetical protein TTHERM_00637670 [Tetrahymena thermophila]	gi|118400210	9 kDa		2	2
1062	☑	☆	proton-pumping vacuolar pyrophosphatase [Tetrahymena thermophila]	gi|146163572	87 kDa		2	2
1063	☑	☆	hypothetical protein TTHERM_00780550 [Tetrahymena thermophila]	gi|229593884	28 kDa		2	2
1064	☑	☆	hypothetical protein TTHERM_01020880 [Tetrahymena thermophila]	gi|229594069	54 kDa		2	2
1065	☑	☆	DNL zinc finger family protein [Tetrahymena thermophila]	gi|229594717	42 kDa		2	2
1066	☑	☆	Kelch motif family protein [Tetrahymena thermophila]	gi|118376212	90 kDa		2	2
1067	☑	☆	Dynein light chain 2B, cytoplasmic, putative [Tetrahymena thermophila]	gi|118346777	12 kDa		2	2
1068	☑	☆	hypothetical protein TTHERM_00765330 [Tetrahymena thermophila]	gi|146182840	16 kDa		2	2
1069	☑	☆	Ubiquitin-conjugating enzyme family protein [Tetrahymena thermophila]	gi|118371010	17 kDa		2	2
1070	☑	☆	hypothetical protein TTHERM_00716190 [Tetrahymena thermophila]	gi|118399301	24 kDa		2	2
1071	☑	☆	Cytochrome P450 family protein [Tetrahymena thermophila]	gi|118357860	61 kDa		2	2
1072	☑	☆	hypothetical protein TTHERM_00024220 [Tetrahymena thermophila]	gi|118350454	21 kDa			2
1073	☑	☆	hypothetical protein IMG5_168850 [Ichthyophthirius multifiliis]	gi|340502063	51 kDa	★		2

Probability Legend

- over 95%
- 80% to 94%
- 50% to 79%
- 20% to 49%
- 0% to 19%

#	Visible?	Starred?	Bio View: Identified Proteins (1837)	Accession Number	Molecular Weight	Protein Grouping Ambiguity	01 Control	02 Sample
1074	☑	☆	hypothetical protein TTHERM_00713350 [Tetrahymena thermophila]	gi|118385516	11 kDa			2
1075	☑	☆	RecName: Full=Tubulin beta chain; AltName: Full=Beta-tubulin	gi|21542248 (+1)	50 kDa	★		2
1076	☑	☆	Fatty acid hydroxylase family protein [Tetrahymena thermophila]	gi|118371365	44 kDa			2
1077	☑	☆	50S ribosomal protein L7Ae, putative [Tetrahymena thermophila]	gi|146175071	14 kDa			2
1078	☑	☆	hypothetical protein TTHERM_00653950 [Tetrahymena thermophila]	gi|118361437	32 kDa			2
1079	☑	☆	hypothetical protein TTHERM_01043090 [Tetrahymena thermophila]	gi|118396634 (+1)	25 kDa			2
1080	☑	☆	PWI domain containing protein [Tetrahymena thermophila]	gi|118346895	129 kDa			2
1081	☑	☆	conserved hypothetical protein [Tetrahymena thermophila]	gi|118350963	140 kDa			2
1082	☑	☆	hypothetical protein TTHERM_00355660 [Tetrahymena thermophila]	gi|118354403	33 kDa			2
1083	☑	☆	hypothetical protein TTHERM_00285560 [Tetrahymena thermophila]	gi|118370740	59 kDa			2
1084	☑	☆	hypothetical protein TTHERM_00289050 [Tetrahymena thermophila]	gi|118370844	18 kDa			2
1085	☑	☆	Initiation factor 2 subunit family protein [Tetrahymena thermophila]	gi|118377342	55 kDa			2
1086	☑	☆	hypothetical protein TTHERM_00535550 [Tetrahymena thermophila]	gi|118380601	16 kDa			2
1087	☑	☆	hypothetical protein TTHERM_00694540 [Tetrahymena thermophila]	gi|118380913	149 kDa			2
1088	☑	☆	Cullin family protein [Tetrahymena thermophila]	gi|118386061	88 kDa			2
1089	☑	☆	XRN 5'-3' exonuclease N-terminus family protein [Tetrahymena thermophila]	gi|118388580	209 kDa			2
1090	☑	☆	conserved hypothetical protein [Tetrahymena thermophila]	gi|118396968	16 kDa			2
1091	☑	☆	PCI domain containing protein [Tetrahymena thermophila]	gi|146163417	44 kDa			2
1092	☑	☆	hypothetical protein TTHERM_00824040 [Tetrahymena thermophila]	gi|146185301	236 kDa			2
1093	☑	☆	Protein kinase domain containing protein [Tetrahymena thermophila]	gi|118388688	72 kDa			2
1094	☑	☆	hypothetical protein TTHERM_00785870 [Tetrahymena thermophila]	gi|118377897	18 kDa			2
1095	☑	☆	EF hand family protein [Tetrahymena thermophila]	gi|118361816	58 kDa			2
1096	☑	☆	hypothetical protein TTHERM_00355330 [Tetrahymena thermophila]	gi|118354337	78 kDa			2
1097	☑	☆	maleylacetoacetate isomerase [Tetrahymena thermophila]	gi|118352112	25 kDa			2
1098	☑	☆	Nucleotidyl transferase family protein [Tetrahymena thermophila]	gi|146175074	81 kDa			2
1099	☑	☆	FHA domain containing protein [Tetrahymena thermophila]	gi|118355666	60 kDa			2
1100	☑	☆	ribosome biogenesis regulatory protein (RRS1) [Tetrahymena thermophila]	gi|118356857	36 kDa			2
1101	☑	☆	Protein kinase domain containing protein [Tetrahymena thermophila]	gi|118357363	39 kDa			2
1102	☑	☆	hypothetical protein TTHERM_00086970 [Tetrahymena thermophila]	gi|118359008	47 kDa			2
1103	☑	☆	hypothetical protein TTHERM_00052480 [Tetrahymena thermophila]	gi|118363116	116 kDa			2
1104	☑	☆	eukaryotic translation initiation factor 3, putative [Tetrahymena thermophila]	gi|118367941	38 kDa	★		2
1105	☑	☆	TPR Domain containing protein [Tetrahymena thermophila]	gi|118369702	116 kDa			2
1106	☑	☆	von Willebrand factor type A domain containing protein [Tetrahymena thermophila]	gi|118372759	57 kDa			2
1107	☑	☆	XRN 5'-3' exonuclease N-terminus family protein [Tetrahymena thermophila]	gi|118373913	104 kDa			2
1108	☑	☆	TRNA binding domain containing protein [Tetrahymena thermophila]	gi|118378491	32 kDa			2
1109	☑	☆	adenylate kinases family protein [Tetrahymena thermophila]	gi|118378957	28 kDa			2
1110	☑	☆	hypothetical protein TTHERM_00455100 [Tetrahymena thermophila]	gi|118381913	18 kDa			2

Probability Legend

- over 95%
- 80% to 94%
- 50% to 79%
- 20% to 49%
- 0% to 19%

#	Visible?	Starred?	Bio View: Identified Proteins (1837)	Accession Number	Molecular Weight	Protein Grouping Ambiguity	01 Control	02 Sample
1111	☑	☆	hypothetical protein TTHERM_01287980 [Tetrahymena thermophila]	gi|118384383	47 kDa			2
1112	☑	☆	hypothetical protein TTHERM_01092380 [Tetrahymena thermophila]	gi|118384602	302 kDa			2
1113	☑	☆	Glutathione S-transferase, N-terminal domain containing protein [Tetrahymena thermophila]	gi|118389232	30 kDa			2
1114	☑	☆	8-amino-7-oxononanoate synthase [Tetrahymena thermophila]	gi|118395835	54 kDa			2
1115	☑	☆	conserved hypothetical protein [Tetrahymena thermophila]	gi|118399786	177 kDa			2
1116	☑	☆	Actin family protein [Tetrahymena thermophila]	gi|146162754	51 kDa			2
1117	☑	☆	hypothetical protein TTHERM_00102740 [Tetrahymena thermophila]	gi|146163739	72 kDa			2
1118	☑	☆	Zinc finger, C2H2 type family protein [Tetrahymena thermophila]	gi|146174270	53 kDa			2
1119	☑	☆	PX domain containing protein [Tetrahymena thermophila]	gi|146179046	71 kDa			2
1120	☑	☆	hypothetical protein TTHERM_00989460 [Tetrahymena thermophila]	gi|146181272	17 kDa			2
1121	☑	☆	ubiquitin specific protease 39 and snrnp assembly factor [Tetrahymena thermophila]	gi|146186096	62 kDa			2
1122	☑	☆	oxidoreductase, short chain dehydrogenase/reductase family protein [Tetrahymena thermophila]	gi|146161927	28 kDa			2
1123	☑	☆	Cofilin/tropomyosin-type actin-binding protein [Tetrahymena thermophila]	gi|118375500	16 kDa			2
1124	☑	☆	hypothetical protein TTHERM_00302050 [Tetrahymena thermophila]	gi|118382946	37 kDa		3	2
1125	☑	☆	Ras family protein [Tetrahymena thermophila]	gi|146182702 (+1)	24 kDa		4	2
1126	☑	☆	hypothetical protein TTHERM_00037740 [Tetrahymena thermophila]	gi|118346407	563 kDa	★		2
1127	☑	☆	predicted protein [Tetrahymena thermophila]	gi|229594775	81 kDa		3	2
1128	☑	☆	SPRY domain containing protein [Tetrahymena thermophila]	gi|146182844	41 kDa		2	2
1129	☑	☆	Clathrin adaptor complex small chain family protein [Tetrahymena thermophila]	gi|118360407	17 kDa			2
1130	☑	☆	ATPase, AAA family protein [Tetrahymena thermophila]	gi|146180922	97 kDa		11	2
1131	☑	☆	hypothetical protein TTHERM_00192030 [Tetrahymena thermophila]	gi|118367127	61 kDa		6	2
1132	☑	☆	hypothetical protein TTHERM_01289060 [Tetrahymena thermophila]	gi|118394899	8 kDa		2	2
1133	☑	☆	Peptidase family M1 containing protein [Tetrahymena thermophila]	gi|118379482	70 kDa			2
1134	☑	☆	Patatin-like phospholipase family protein [Tetrahymena thermophila]	gi|118355534	41 kDa			2
1135	☑	☆	S-antigen protein [Tetrahymena thermophila]	gi|118389844	310 kDa		3	2
1136	☑	☆	hypothetical protein TTHERM_00538790 [Tetrahymena thermophila]	gi|118348860	57 kDa		4	2
1137	☑	☆	hypothetical protein TTHERM_00641119 [Tetrahymena thermophila]	gi|146165550	17 kDa			2
1138	☑	☆	hypothetical protein TTHERM_00471950 [Tetrahymena thermophila]	gi|229595014	47 kDa		7	2
1139	☑	☆	hypothetical protein TTHERM_00825290 [Tetrahymena thermophila]	gi|118398135	34 kDa			2
1140	☑	☆	hypothetical protein TTHERM_00056000 [Tetrahymena thermophila]	gi|118348112	57 kDa		6	2
1141	☑	☆	hypothetical protein TTHERM_00091620 [Tetrahymena thermophila]	gi|118359146 (+1)	328 kDa		7	2
1142	☑	☆	histone acetyltransferase, ELP3 family protein [Tetrahymena thermophila]	gi|146185746	67 kDa			2
1143	☑	☆	Papain family cysteine protease containing protein [Tetrahymena thermophila]	gi|118364222	37 kDa		4	2
1144	☑	☆	Major Facilitator Superfamily protein [Tetrahymena thermophila]	gi|118381192	62 kDa		2	2
1145	☑	☆	cyclic nucleotide-binding domain containing protein [Tetrahymena thermophila]	gi|146181312	48 kDa		2	2
1146	☑	☆	MutS domain III family protein [Tetrahymena thermophila]	gi|118376906 (+1)	158 kDa		6	2
1147	☑	☆	major facilitator superfamily protein [Tetrahymena thermophila]	gi|146185919	60 kDa		3	2

Probability Legend
- over 95%
- 80% to 94%
- 50% to 79%
- 20% to 49%
- 0% to 19%

#	Visible?	Starred?	Bio View: Identified Proteins (1837)	Accession Number	Molecular Weight	Protein Grouping Ambiguity	01 Control	02 Sample
1148	☑	☆	Adenylate kinase family protein [Tetrahymena thermophila]	gi|118382001	51 kDa		2	2
1149	☑	☆	protozoan/cyanobacterial globin family protein [Tetrahymena thermophila]	gi|118355044	19 kDa		2	2
1150	☑	☆	protein phsophatase-2a [Tetrahymena thermophila]	gi|146180938 (+1)	43 kDa			2
1151	☑	☆	Adaptin N-terminal region family protein [Tetrahymena thermophila]	gi|118374843	113 kDa		4	2
1152	☑	☆	hypothetical protein TTHERM_00618840 [Tetrahymena thermophila]	gi|229595372	136 kDa			2
1153	☑	☆	Penicillin amidase family protein [Tetrahymena thermophila]	gi|118390269	103 kDa		11	2
1154	☑	☆	hypothetical protein TTHERM_01093690 [Tetrahymena thermophila]	gi|229593990	66 kDa		16	2
1155	☑	☆	V-type ATPase 116kDa subunit family protein [Tetrahymena thermophila]	gi|118352620	233 kDa		4	2
1156	☑	☆	hypothetical protein TTHERM_00954340 [Tetrahymena thermophila]	gi|229595357	34 kDa		3	2
1157	☑	☆	hypothetical protein TTHERM_00622800 [Tetrahymena thermophila]	gi|118378662	113 kDa		5	2
1158	☑	☆	Protein kinase domain containing protein [Tetrahymena thermophila]	gi|146183980	79 kDa			2
1159	☑	☆	hypothetical protein TTHERM_00509080 [Tetrahymena thermophila]	gi|118363979	44 kDa		3	2
1160	☑	☆	metallopeptidase family M24 containing protein [Tetrahymena thermophila]	gi|118354100	41 kDa			2
1161	☑	☆	ATPase, putative [Tetrahymena thermophila]	gi|118364083	122 kDa		7	2
1162	☑	☆	Glutathione S-transferase, N-terminal domain containing protein [Tetrahymena thermophila]	gi|118389068	23 kDa			2
1163	☑	☆	RNA binding protein [Tetrahymena thermophila]	gi|146184999	43 kDa		9	2
1164	☑	☆	hypothetical protein TTHERM_01015860 [Tetrahymena thermophila]	gi|229594387	194 kDa		6	2
1165	☑	☆	hypothetical protein TTHERM_01044770 [Tetrahymena thermophila]	gi|118396623	32 kDa		2	2
1166	☑	☆	hypothetical protein TTHERM_01021920 [Tetrahymena thermophila]	gi|118352397	104 kDa		9	2
1167	☑	☆	hypothetical protein TTHERM_00462850 [Tetrahymena thermophila]	gi|146173285	28 kDa			2
1168	☑	☆	Kinesin motor domain containing protein [Tetrahymena thermophila]	gi|118388276	144 kDa		8	2
1169	☑	☆	RNA binding protein [Tetrahymena thermophila]	gi|118386360	19 kDa			2
1170	☑	☆	hypothetical protein TTHERM_00194400 [Tetrahymena thermophila]	gi|118367967	12 kDa			2
1171	☑	☆	hypothetical protein TTHERM_00688660 [Tetrahymena thermophila]	gi|146183749	49 kDa		3	2
1172	☑	☆	Glutathione S-transferase, N-terminal domain containing protein [Tetrahymena thermophila]	gi|118350781 (+1)	25 kDa			2
1173	☑	☆	Thioredoxin family protein [Tetrahymena thermophila]	gi|229595814	42 kDa		3	2
1174	☑	☆	enoyl-CoA hydratase/isomerase family protein [Tetrahymena thermophila]	gi|118348110	31 kDa			2
1175	☑	☆	hypothetical protein TTHERM_00516380 [Tetrahymena thermophila]	gi|118364053	36 kDa	★	10	2
1176	☑	☆	Protein kinase domain containing protein [Tetrahymena thermophila]	gi|118380328	208 kDa		4	2
1177	☑	☆	Sm protein [Tetrahymena thermophila]	gi|118387719	262 kDa		2	2
1178	☑	☆	hypothetical protein TTHERM_00242500 [Tetrahymena thermophila]	gi|118383637	75 kDa			2
1179	☑	☆	hypothetical protein TTHERM_00502190 [Tetrahymena thermophila]	gi|118378194	74 kDa		4	2
1180	☑	☆	hypothetical protein TTHERM_00492440 [Tetrahymena thermophila]	gi|146181620	33 kDa			2
1181	☑	☆	hypothetical protein TTHERM_01339580 [Tetrahymena thermophila]	gi|229593620	33 kDa			2
1182	☑	☆	Mitochondrial ribosomal protein L51 / S25 / CI-B8 domain containing protein [Tetrahymena thermophila]	gi|118381896	17 kDa			2
1183	☑	☆	hypothetical protein TTHERM_00537400 [Tetrahymena thermophila]	gi|118380773	260 kDa		2	2
1184	☑	☆	hypothetical protein TTHERM_00473110 [Tetrahymena thermophila]	gi|118381467	29 kDa			2

Probability Legend

- over 95%
- 80% to 94%
- 50% to 79%
- 20% to 49%
- 0% to 19%

#	Visible?	Starred?	Bio View: Identified Proteins (1837)	Accession Number	Molecular Weight	Protein Grouping Ambiguity	01 Control	02 Sample
1185	☑	☆	hypothetical protein TTHERM_01122690 [Tetrahymena thermophila]	gi|118372755	15 kDa		5	2
1186	☑	☆	Guanylate-binding protein, N-terminal domain containing protein [Tetrahymena thermophila]	gi|118388876	101 kDa		10	2
1187	☑	☆	hypothetical protein TTHERM_00794120 [Tetrahymena thermophila]	gi|118375494	14 kDa		2	2
1188	☑	☆	hypothetical protein TTHERM_00127269 [Tetrahymena thermophila]	gi|146167369	40 kDa			2
1189	☑	☆	signal peptide peptidase family protein [Tetrahymena thermophila]	gi|118397869	49 kDa			2
1190	☑	☆	Leucine Rich Repeat family protein [Tetrahymena thermophila]	gi|146163566	209 kDa		13	2
1191	☑	☆	hypothetical protein TTHERM_00695820 [Tetrahymena thermophila]	gi|118384909	102 kDa			2
1192	☑	☆	hypothetical protein TTHERM_00777260 [Tetrahymena thermophila]	gi|118376157	36 kDa	★	5	2
1193	☑	☆	RNA polymerase Rpb8 family protein [Tetrahymena thermophila]	gi|118345758	17 kDa			2
1194	☑	☆	hypothetical protein TTHERM_00394600 [Tetrahymena thermophila]	gi|118357365	26 kDa			2
1195	☑	☆	hypothetical protein TTHERM_00497310 [Tetrahymena thermophila]	gi|229593563	19 kDa		3	2
1196	☑	☆	Helicase conserved C-terminal domain containing protein [Tetrahymena thermophila]	gi|118357243	55 kDa			2
1197	☑	☆	hypothetical protein TTHERM_00522940 [Tetrahymena thermophila]	gi|118362490	7 kDa			2
1198	☑	☆	hypothetical protein TTHERM_00449020 [Tetrahymena thermophila]	gi|118360130	116 kDa			2
1199	☑	☆	hypothetical protein TTHERM_00006010 [Tetrahymena thermophila]	gi|118349602	93 kDa		10	2
1200	☑	☆	conserved hypothetical protein [Tetrahymena thermophila]	gi|118395330	66 kDa			2
1201	☑	☆	hypothetical protein TTHERM_00442910 [Tetrahymena thermophila]	gi|118401768	62 kDa			2
1202	☑	☆	hypothetical protein TTHERM_00151580 [Tetrahymena thermophila]	gi|229594874	12 kDa		3	2
1203	☑	☆	Protein kinase domain containing protein [Tetrahymena thermophila]	gi|118386071	35 kDa		4	2
1204	☑	☆	Adaptor complexes medium subunit family protein [Tetrahymena thermophila]	gi|146182191	51 kDa			2
1205	☑	☆	hypothetical protein TTHERM_00989510 [Tetrahymena thermophila]	gi|229594701	65 kDa			2
1206	☑	☆	hypothetical protein TTHERM_00146450 [Tetrahymena thermophila]	gi|118356050	37 kDa		2	2
1207	☑	☆	DNA topoisomerase family protein [Tetrahymena thermophila]	gi|146182668	100 kDa			2
1208	☑	☆	hypothetical protein TTHERM_00647070 [Tetrahymena thermophila]	gi|118388640	113 kDa			2
1209	☑	☆	Ribophorin I family protein [Tetrahymena thermophila]	gi|118363208	54 kDa		4	2
1210	☑	☆	hypothetical protein TTHERM_00335640 [Tetrahymena thermophila]	gi|118368652	173 kDa		7	2
1211	☑	☆	hypothetical protein TTHERM_00239090 [Tetrahymena thermophila]	gi|118383359	94 kDa			2
1212	☑	☆	hypothetical protein TTHERM_00160690 [Tetrahymena thermophila]	gi|146162619	12 kDa			2
1213	☑	☆	Synaptobrevin family protein [Tetrahymena thermophila]	gi|118363280	26 kDa		2	2
1214	☑	☆	C2 domain containing protein [Tetrahymena thermophila]	gi|118369298	170 kDa		15	2
1215	☑	☆	Inorganic H+ pyrophosphatase [Tetrahymena thermophila]	gi|118356655	81 kDa	★		2
1216	☑	☆	Protein kinase domain containing protein [Tetrahymena thermophila]	gi|118387530 (+3)	58 kDa			2
1217	☑	☆	hypothetical protein TTHERM_00046250 [Tetrahymena thermophila]	gi|118363282	101 kDa		16	2
1218	☑	☆	Ribosomal L39 protein [Tetrahymena thermophila]	gi|118381535 (+2)	11 kDa		2	2
1219	☑	☆	Insulinase (Peptidase family M16) [Tetrahymena thermophila]	gi|146183516	55 kDa			2
1220	☑	☆	Acyl-CoA oxidase family protein [Tetrahymena thermophila]	gi|118371253	80 kDa			2
1221	☑	☆	hypothetical protein TTHERM_00903870 [Tetrahymena thermophila]	gi|118387922	15 kDa		2	2

Probability Legend

- over 95%
- 80% to 94%
- 50% to 79%
- 20% to 49%
- 0% to 19%

#	Visible?	Starred?	Bio View: Identified Proteins (1837)	Accession Number	Molecular Weight	Protein Grouping Ambiguity	01 Control	02 Sample
1222	☑	☆	DEAD/DEAH box helicase family protein [Tetrahymena thermophila]	gi|118362432	48 kDa			2
1223	☑	☆	IFT81/Intraflagellar transport protein 81 [Tetrahymena thermophila]	gi|118345489	84 kDa		2	2
1224	☑	☆	hypothetical protein TTHERM_00001120 [Tetrahymena thermophila]	gi|118349422	84 kDa		4	2
1225	☑	☆	hypothetical protein TTHERM_00399440 [Tetrahymena thermophila]	gi|118361578	151 kDa		8	2
1226	☑	☆	hypothetical protein TTHERM_00145360 [Tetrahymena thermophila]	gi|118355834	15 kDa			2
1227	☑	☆	hypothetical protein TTHERM_00205170 [Tetrahymena thermophila]	gi|146161421	18 kDa		7	2
1228	☑	☆	Nuclear movement protein [Tetrahymena thermophila]	gi|118396057	37 kDa			2
1229	☑	☆	hypothetical protein TTHERM_00053820 [Tetrahymena thermophila]	gi|118348072	40 kDa			2
1230	☑	☆	Adaptin N-terminal region family protein [Tetrahymena thermophila]	gi|118361963	95 kDa		3	2
1231	☑	☆	Protein phosphatase 2C containing protein [Tetrahymena thermophila]	gi|146161639	36 kDa			2
1232	☑	☆	hypothetical protein TTHERM_00850630 [Tetrahymena thermophila]	gi|146174104	13 kDa		2	2
1233	☑	☆	oxidoreductase, short chain dehydrogenase/reductase family protein [Tetrahymena thermophila]	gi|118396114	31 kDa			2
1234	☑	☆	Ubiquitin carboxyl-terminal hydrolase family protein [Tetrahymena thermophila]	gi|118395227 (+1)	265 kDa		4	2
1235	☑	☆	hypothetical protein TTHERM_00170300 [Tetrahymena thermophila]	gi|118350823	149 kDa		19	
1236	☑	☆	Dynein heavy chain family protein [Tetrahymena thermophila]	gi|118378501	488 kDa	★	16	
1237	☑	☆	EF hand family protein [Tetrahymena thermophila]	gi|118399993	179 kDa		14	
1238	☑	☆	nucleoporin Nup155 [Tetrahymena thermophila]	gi|289666659	239 kDa		14	
1239	☑	☆	hypothetical protein TTHERM_00474640 [Tetrahymena thermophila]	gi|118381573	219 kDa		13	
1240	☑	☆	hypothetical protein TTHERM_00006090 [Tetrahymena thermophila]	gi|118349618	95 kDa		12	
1241	☑	☆	Zinc finger family protein [Tetrahymena thermophila]	gi|118353257	414 kDa	★	11	
1242	☑	☆	Adaptin N-terminal region family protein [Tetrahymena thermophila]	gi|118397830	109 kDa		11	
1243	☑	☆	flavodoxin family protein [Tetrahymena thermophila]	gi|118345517	77 kDa		11	
1244	☑	☆	hypothetical protein TTHERM_00388480 [Tetrahymena thermophila]	gi|118372311	54 kDa		11	
1245	☑	☆	hypothetical protein TTHERM_00537180 [Tetrahymena thermophila]	gi|118380729	62 kDa		11	
1246	☑	☆	hypothetical protein TTHERM_00261800 [Tetrahymena thermophila]	gi|118351506	39 kDa		11	
1247	☑	☆	Kinesin motor domain containing protein [Tetrahymena thermophila]	gi|118401618	171 kDa		11	
1248	☑	☆	Brg1p [Tetrahymena thermophila]	gi|31322808	145 kDa		11	
1249	☑	☆	Suppressor of kinetochore protein 1 [Tetrahymena thermophila]	gi|118361159 (+1)	29 kDa		10	
1250	☑	☆	hypothetical protein TTHERM_00551040 [Tetrahymena thermophila]	gi|118351833	560 kDa		10	
1251	☑	☆	DNA topoisomerase family protein [Tetrahymena thermophila]	gi|118389870	106 kDa		10	
1252	☑	☆	hypothetical protein TTHERM_00522820 [Tetrahymena thermophila]	gi|146165131	54 kDa		10	
1253	☑	☆	PA domain containing protein [Tetrahymena thermophila]	gi|146184097	55 kDa		10	
1254	☑	☆	Ubiquitin carboxyl-terminal hydrolase family protein [Tetrahymena thermophila]	gi|118379729	422 kDa		10	
1255	☑	☆	hypothetical protein TTHERM_00590150 [Tetrahymena thermophila]	gi|118373459	89 kDa		10	
1256	☑	☆	Dynein heavy chain family protein [Tetrahymena thermophila]	gi|118377765	520 kDa	★	9	
1257	☑	☆	Dynein heavy chain family protein [Tetrahymena thermophila]	gi|118378437 (+1)	494 kDa		9	
1258	☑	☆	EF hand family protein [Tetrahymena thermophila]	gi|118362512	179 kDa		9	

Probability Legend

- over 95%
- 80% to 94%
- 50% to 79%
- 20% to 49%
- 0% to 19%

#	Visible?	Starred?	Bio View: Identified Proteins (1837)	Accession Number	Molecular Weight	Protein Grouping Ambiguity	01 Control	02 Sample
1259	☑	☆	hypothetical protein TTHERM_00218910 [Tetrahymena thermophila]	gi|118374751	34 kDa		9	
1260	☑	☆	hypothetical protein TTHERM_00392760 [Tetrahymena thermophila]	gi|118357205	175 kDa		9	
1261	☑	☆	EF hand family protein [Tetrahymena thermophila]	gi|146180948	88 kDa		9	
1262	☑	☆	DHHC zinc finger domain containing protein [Tetrahymena thermophila]	gi|146183347	117 kDa		9	
1263	☑	☆	hypothetical protein TTHERM_00773710 [Tetrahymena thermophila]	gi|229594562	41 kDa		9	
1264	☑	☆	TPR Domain containing protein [Tetrahymena thermophila]	gi|118387255	167 kDa	★	8	
1265	☑	☆	Cullin family protein [Tetrahymena thermophila]	gi|118359032	92 kDa		8	
1266	☑	☆	EF hand family protein [Tetrahymena thermophila]	gi|118372327	119 kDa		8	
1267	☑	☆	Dynein heavy chain family protein [Tetrahymena thermophila]	gi|118367791	479 kDa	★	8	
1268	☑	☆	hypothetical protein TTHERM_00728900 [Tetrahymena thermophila]	gi|118379017	46 kDa		8	
1269	☑	☆	CAP-Gly domain containing protein [Tetrahymena thermophila]	gi|118398234	144 kDa		8	
1270	☑	☆	hypothetical protein TTHERM_00047650 [Tetrahymena thermophila]	gi|146165304	99 kDa		8	
1271	☑	☆	phospholipid-translocating P-type ATPase, flippase family protein [Tetrahymena thermophila]	gi|118374377	349 kDa		8	
1272	☑	☆	Protein kinase domain containing protein [Tetrahymena thermophila]	gi|118359918	89 kDa		8	
1273	☑	☆	Protein kinase domain containing protein [Tetrahymena thermophila]	gi|229594993	108 kDa		8	
1274	☑	☆	hypothetical protein TTHERM_00321730 [Tetrahymena thermophila]	gi|118359467	104 kDa		8	
1275	☑	☆	hypothetical protein TTHERM_00298320 [Tetrahymena thermophila]	gi|118382620	288 kDa		8	
1276	☑	☆	Kinesin motor domain containing protein [Tetrahymena thermophila]	gi|118379637	166 kDa		8	
1277	☑	☆	actin-related protein [Tetrahymena thermophila]	gi|25361128	44 kDa		8	
1278	☑	☆	hypothetical protein TTHERM_00773210 [Tetrahymena thermophila]	gi|229594553	72 kDa		7	
1279	☑	☆	hypothetical protein TTHERM_00267890 [Tetrahymena thermophila]	gi|146166097	85 kDa		7	
1280	☑	☆	Dynein heavy chain family protein [Tetrahymena thermophila]	gi|118396733	496 kDa	★	7	
1281	☑	☆	hypothetical protein TTHERM_00627000 [Tetrahymena thermophila]	gi|146174811	88 kDa		7	
1282	☑	☆	hypothetical protein TTHERM_00827050 [Tetrahymena thermophila]	gi|118398042	108 kDa		7	
1283	☑	☆	hypothetical protein TTHERM_00703970 [Tetrahymena thermophila]	gi|146185560	69 kDa		7	
1284	☑	☆	hypothetical protein TTHERM_01093580 [Tetrahymena thermophila]	gi|118396023	53 kDa		7	
1285	☑	☆	hypothetical protein TTHERM_00835100 [Tetrahymena thermophila]	gi|118397885	36 kDa		7	
1286	☑	☆	conserved hypothetical protein [Tetrahymena thermophila]	gi|118370119	146 kDa		7	
1287	☑	☆	hypothetical protein TTHERM_00218750 [Tetrahymena thermophila]	gi|118374719	266 kDa		7	
1288	☑	☆	conserved hypothetical protein [Tetrahymena thermophila]	gi|118388389	45 kDa		7	
1289	☑	☆	ADL128Cp, putative [Tetrahymena thermophila]	gi|118350092	133 kDa		7	
1290	☑	☆	hypothetical protein TTHERM_00576790 [Tetrahymena thermophila]	gi|118352200	102 kDa		7	
1291	☑	☆	hypothetical protein TTHERM_00305630 [Tetrahymena thermophila]	gi|118375703	231 kDa		7	
1292	☑	☆	hypothetical protein TTHERM_00967570 [Tetrahymena thermophila]	gi|118351341	72 kDa	★	6	
1293	☑	☆	PX domain containing protein [Tetrahymena thermophila]	gi|146163033	67 kDa		6	
1294	☑	☆	hypothetical protein TTHERM_00237520 [Tetrahymena thermophila]	gi|146182531	67 kDa		6	
1295	☑	☆	hypothetical protein TTHERM_00030340 [Tetrahymena thermophila]	gi|146161244	41 kDa	★	6	

Probability Legend

- over 95%
- 80% to 94%
- 50% to 79%
- 20% to 49%
- 0% to 19%

#	Visible?	Starred?	Bio View: Identified Proteins (1837)	Accession Number	Molecular Weight	Protein Grouping Ambiguity	01 Control	02 Sample
1296	☑	☆	hypothetical protein TTHERM_00029960 [Tetrahymena thermophila]	gi|118346569	40 kDa		6	
1297	☑	☆	hypothetical protein TTHERM_00136430 [Tetrahymena thermophila]	gi|118373060	146 kDa		6	
1298	☑	☆	TPR Domain containing protein [Tetrahymena thermophila]	gi|118375831	52 kDa		6	
1299	☑	☆	Leucine Rich Repeat family protein [Tetrahymena thermophila]	gi|118370718	183 kDa		6	
1300	☑	☆	hypothetical protein TTHERM_00136490 [Tetrahymena thermophila]	gi|118373072	56 kDa		6	
1301	☑	☆	Phosphatidylinositol 3- and 4-kinase family protein [Tetrahymena thermophila]	gi|118376626	298 kDa		6	
1302	☑	☆	C2 domain containing protein [Tetrahymena thermophila]	gi|118398056	179 kDa		6	
1303	☑	☆	hypothetical protein TTHERM_00655460 [Tetrahymena thermophila]	gi|118399714	83 kDa		6	
1304	☑	☆	hypothetical protein TTHERM_00584510 [Tetrahymena thermophila]	gi|118400472	225 kDa		6	
1305	☑	☆	hypothetical protein TTHERM_00220650 [Tetrahymena thermophila]	gi|146179632	57 kDa		6	
1306	☑	☆	CCR4-Not complex component, Not1 family protein [Tetrahymena thermophila]	gi|118357702	291 kDa		6	
1307	☑	☆	ATPase, AAA family protein [Tetrahymena thermophila]	gi|118386221	36 kDa		6	
1308	☑	☆	hypothetical protein TTHERM_01054390 [Tetrahymena thermophila]	gi|118396556	239 kDa		6	
1309	☑	☆	EF hand family protein [Tetrahymena thermophila]	gi|118401425	65 kDa		6	
1310	☑	☆	hypothetical protein TTHERM_00321720 [Tetrahymena thermophila]	gi|146164084	47 kDa		6	
1311	☑	☆	hypothetical protein TTHERM_00685990 [Tetrahymena thermophila]	gi|146182770	141 kDa		6	
1312	☑	☆	hypothetical protein TTHERM_00780490 [Tetrahymena thermophila]	gi|118386169	216 kDa		6	
1313	☑	☆	hypothetical protein TTHERM_00521980 [Tetrahymena thermophila]	gi|118362298	323 kDa		6	
1314	☑	☆	hypothetical protein TTHERM_00058840 [Tetrahymena thermophila]	gi|146161665	56 kDa		6	
1315	☑	☆	hypothetical protein TTHERM_00833610 [Tetrahymena thermophila]	gi|118360874	97 kDa	★	6	
1316	☑	☆	Calpain family cysteine protease containing protein [Tetrahymena thermophila]	gi|118377187	99 kDa		6	
1317	☑	☆	Tubulin-tyrosine ligase family protein [Tetrahymena thermophila]	gi|118400871	75 kDa		6	
1318	☑	☆	ATPase, AAA family protein [Tetrahymena thermophila]	gi|118359798	181 kDa		6	
1319	☑	☆	MYND finger family protein [Tetrahymena thermophila]	gi|118387165	150 kDa		6	
1320	☑	☆	hypothetical protein TTHERM_00773790 [Tetrahymena thermophila]	gi|118398608	153 kDa		6	
1321	☑	☆	hypothetical protein TTHERM_00085510 [Tetrahymena thermophila]	gi|146163934	44 kDa		6	
1322	☑	☆	hypothetical protein TTHERM_00426280 [Tetrahymena thermophila]	gi|118361151	128 kDa		6	
1323	☑	☆	hypothetical protein TTHERM_00348140 [Tetrahymena thermophila]	gi|118379607	238 kDa	★	6	
1324	☑	☆	Adaptin N-terminal region family protein [Tetrahymena thermophila]	gi|118371508	144 kDa		6	
1325	☑	☆	hypothetical protein TTHERM_00410150 [Tetrahymena thermophila]	gi|118375246	230 kDa		6	
1326	☑	☆	hypothetical protein TTHERM_00019680 [Tetrahymena thermophila]	gi|118350338	101 kDa		6	
1327	☑	☆	hypothetical protein TTHERM_00690020 [Tetrahymena thermophila]	gi|118387805	48 kDa		5	
1328	☑	☆	hypothetical protein TTHERM_00535160 [Tetrahymena thermophila]	gi|146181810	59 kDa		5	
1329	☑	☆	Leucine Rich Repeat family protein [Tetrahymena thermophila]	gi|146184056	77 kDa		5	
1330	☑	☆	calcium-translocating P-type ATPase, PMCA-type family protein [Tetrahymena thermophila]	gi|118372229	111 kDa	★	5	
1331	☑	☆	Eukaryotic DNA topoisomerase I, catalytic core family protein [Tetrahymena thermophila]	gi|118384307	83 kDa		5	
1332	☑	☆	hypothetical protein TTHERM_00502340 [Tetrahymena thermophila]	gi|146181180	68 kDa		5	

Probability Legend: over 95% | 80% to 94% | 50% to 79% | 20% to 49% | 0% to 19%

#	Visible?	Starred?	Bio View: Identified Proteins (1837)	Accession Number	Molecular Weight	Protein Grouping Ambiguity	01 Control	02 Sample
1333	☑	☆	C2 domain containing protein [Tetrahymena thermophila]	gi|146186084	93 kDa		5	
1334	☑	☆	ABC transporter N-terminus family protein [Tetrahymena thermophila]	gi|118377667	80 kDa		5	
1335	☑	☆	E1-E2 ATPase family protein [Tetrahymena thermophila]	gi|118362569	109 kDa	★	5	
1336	☑	☆	hypothetical protein TTHERM_01044620 [Tetrahymena thermophila]	gi|118396593	63 kDa		5	
1337	☑	☆	hypothetical protein TTHERM_00540130 [Tetrahymena thermophila]	gi|118348930	144 kDa		5	
1338	☑	☆	hypothetical protein TTHERM_00185310 [Tetrahymena thermophila]	gi|118351047	33 kDa		5	
1339	☑	☆	hypothetical protein TTHERM_00371110 [Tetrahymena thermophila]	gi|146162502	139 kDa		5	
1340	☑	☆	hypothetical protein TTHERM_00463800 [Tetrahymena thermophila]	gi|118371355	68 kDa		5	
1341	☑	☆	hypothetical protein TTHERM_00141110 [Tetrahymena thermophila]	gi|118355578	46 kDa		5	
1342	☑	☆	hypothetical protein TTHERM_00729020 [Tetrahymena thermophila]	gi|118379039	159 kDa		5	
1343	☑	☆	hypothetical protein TTHERM_00056189 [Tetrahymena thermophila]	gi|146161697	42 kDa		5	
1344	☑	☆	ATPase, AAA family protein [Tetrahymena thermophila]	gi|146181988	42 kDa		5	
1345	☑	☆	von Willebrand factor type A domain containing protein [Tetrahymena thermophila]	gi|118353832	62 kDa	★	5	
1346	☑	☆	hypothetical protein TTHERM_00558470 [Tetrahymena thermophila]	gi|118378467	244 kDa		5	
1347	☑	☆	hypothetical protein TTHERM_00194770 [Tetrahymena thermophila]	gi|118368041	144 kDa		5	
1348	☑	☆	hypothetical protein TTHERM_00773700 [Tetrahymena thermophila]	gi|118398590	44 kDa		5	
1349	☑	☆	hypothetical protein TTHERM_00051700 [Tetrahymena thermophila]	gi|118362902	112 kDa		5	
1350	☑	☆	hypothetical protein TTHERM_00348780 [Tetrahymena thermophila]	gi|118379735	189 kDa		5	
1351	☑	☆	B-box zinc finger family protein [Tetrahymena thermophila]	gi|118384971	57 kDa		5	
1352	☑	☆	hypothetical protein TTHERM_00248430 [Tetrahymena thermophila]	gi|118381422	33 kDa		5	
1353	☑	☆	hypothetical protein TTHERM_00565590 [Tetrahymena thermophila]	gi|146181062	31 kDa		5	
1354	☑	☆	hypothetical protein TTHERM_00292250 [Tetrahymena thermophila]	gi|118371078	549 kDa		5	
1355	☑	☆	conserved hypothetical protein [Tetrahymena thermophila]	gi|118353261	60 kDa		5	
1356	☑	☆	UBX domain containing protein [Tetrahymena thermophila]	gi|229594421	55 kDa		5	
1357	☑	☆	hypothetical protein TTHERM_00042690 [Tetrahymena thermophila]	gi|118346695	74 kDa		5	
1358	☑	☆	Vps52 / Sac2 family protein [Tetrahymena thermophila]	gi|118377705	84 kDa		5	
1359	☑	☆	hypothetical protein TTHERM_00077120 [Tetrahymena thermophila]	gi|118364864	44 kDa		5	
1360	☑	☆	hypothetical protein TTHERM_00463380 [Tetrahymena thermophila]	gi|118371271	28 kDa		5	
1361	☑	☆	Ubiquitin carboxyl-terminal hydrolase family protein [Tetrahymena thermophila]	gi|118364429	361 kDa		5	
1362	☑	☆	GTP-ase activating protein for Arf containing protein [Tetrahymena thermophila]	gi|146185984	69 kDa		5	
1363	☑	☆	hypothetical protein TTHERM_00590170 [Tetrahymena thermophila]	gi|229595114	43 kDa		5	
1364	☑	☆	hypothetical protein TTHERM_00474930 [Tetrahymena thermophila]	gi|146182101	28 kDa		5	
1365	☑	☆	Protein kinase domain containing protein [Tetrahymena thermophila]	gi|146182326	67 kDa		5	
1366	☑	☆	hypothetical protein TTHERM_00985030 [Tetrahymena thermophila]	gi|229596262	125 kDa		5	
1367	☑	☆	SGS domain containing protein [Tetrahymena thermophila]	gi|118401351	49 kDa		5	
1368	☑	☆	hypothetical protein TTHERM_00120830 [Tetrahymena thermophila]	gi|118355042	117 kDa		5	
1369	☑	☆	Ubiquitin carboxyl-terminal hydrolase family protein [Tetrahymena thermophila]	gi|146181648	228 kDa		5	

Probability Legend: over 95% | 80% to 94% | 50% to 79% | 20% to 49% | 0% to 19%

#	Visible?	Starred?	Bio View: Identified Proteins (1837)	Accession Number	Molecular Weight	Protein Grouping Ambiguity	01 Control	02 Sample
1370	☑	☆	hypothetical protein TTHERM_01055420 [Tetrahymena thermophila]	gi|229593633	57 kDa		5	
1371	☑	☆	Kelch motif family protein [Tetrahymena thermophila]	gi|118358286	136 kDa		5	
1372	☑	☆	ATPase, AAA family protein [Tetrahymena thermophila]	gi|118353063	123 kDa		5	
1373	☑	☆	hypothetical protein TTHERM_00449180 [Tetrahymena thermophila]	gi|229596095	44 kDa		5	
1374	☑	☆	DnaJ C-terminal region family protein [Tetrahymena thermophila]	gi|229594886	49 kDa		5	
1375	☑	☆	hypothetical protein TTHERM_00548140 [Tetrahymena thermophila]	gi|118345680	125 kDa		5	
1376	☑	☆	hypothetical protein TTHERM_00431360 [Tetrahymena thermophila]	gi|118356205	198 kDa		5	
1377	☑	☆	hypothetical protein TTHERM_01102760 [Tetrahymena thermophila]	gi|118372504	71 kDa		4	
1378	☑	☆	hypothetical protein TTHERM_00974270 [Tetrahymena thermophila]	gi|146162744	29 kDa		4	
1379	☑	☆	Ribonucleotide reductase, barrel domain containing protein [Tetrahymena thermophila]	gi|118346189	343 kDa		4	
1380	☑	☆	hypothetical protein TTHERM_00313090 [Tetrahymena thermophila]	gi|118349227	40 kDa		4	
1381	☑	☆	hypothetical protein TTHERM_00370840 [Tetrahymena thermophila]	gi|146162515	74 kDa		4	
1382	☑	☆	hypothetical protein TTHERM_00488193 [Tetrahymena thermophila]	gi|146168434	225 kDa		4	
1383	☑	☆	Protein kinase domain containing protein [Tetrahymena thermophila]	gi|118369158	99 kDa		4	
1384	☑	☆	hypothetical protein TTHERM_01028760 [Tetrahymena thermophila]	gi|146165468	84 kDa		4	
1385	☑	☆	hypothetical protein TTHERM_00849260 [Tetrahymena thermophila]	gi|229595197	65 kDa		4	
1386	☑	☆	Penicillin amidase family protein [Tetrahymena thermophila]	gi|118365941	103 kDa		4	
1387	☑	☆	E1-E2 ATPase family protein [Tetrahymena thermophila]	gi|229595713	101 kDa	★	4	
1388	☑	☆	hypothetical protein TTHERM_00770760 [Tetrahymena thermophila]	gi|118361276	152 kDa		4	
1389	☑	☆	hypothetical protein TTHERM_00050550 [Tetrahymena thermophila]	gi|118362800	164 kDa		4	
1390	☑	☆	hypothetical protein TTHERM_00283730 [Tetrahymena thermophila]	gi|118370053	156 kDa		4	
1391	☑	☆	chromodomain containing protein [Tetrahymena thermophila]	gi|118365977 (+1)	55 kDa		4	
1392	☑	☆	conserved hypothetical protein [Tetrahymena thermophila]	gi|146165310	47 kDa		4	
1393	☑	☆	hypothetical protein TTHERM_00445990 [Tetrahymena thermophila]	gi|146181763	178 kDa		4	
1394	☑	☆	Protein kinase domain containing protein [Tetrahymena thermophila]	gi|118388793	77 kDa		4	
1395	☑	☆	Armadillo/beta-catenin repeat family protein [Tetrahymena thermophila]	gi|118396891	60 kDa		4	
1396	☑	☆	hypothetical protein TTHERM_00529650 [Tetrahymena thermophila]	gi|118400709	94 kDa	★	4	
1397	☑	☆	Leucine Rich Repeat family protein [Tetrahymena thermophila]	gi|118366809	55 kDa		4	
1398	☑	☆	small GTP-binding protein domain containing protein [Tetrahymena thermophila]	gi|146185006	29 kDa		4	
1399	☑	☆	hypothetical protein TTHERM_01276320 [Tetrahymena thermophila]	gi|146184658	125 kDa		4	
1400	☑	☆	hypothetical protein TTHERM_00927130 [Tetrahymena thermophila]	gi|146185174	74 kDa		4	
1401	☑	☆	conserved hypothetical protein [Tetrahymena thermophila]	gi|118373752	181 kDa		4	
1402	☑	☆	DSS1/SEM1 family protein [Tetrahymena thermophila]	gi|118362053	17 kDa		4	
1403	☑	☆	Radial spoke protein 3 containing protein [Tetrahymena thermophila]	gi|118347623	111 kDa		4	
1404	☑	☆	hypothetical protein TTHERM_00300390 [Tetrahymena thermophila]	gi|118382820	21 kDa		4	
1405	☑	☆	Endonuclease/Exonuclease/phosphatase family protein [Tetrahymena thermophila]	gi|146171520	104 kDa		4	
1406	☑	☆	Protein kinase domain containing protein [Tetrahymena thermophila]	gi|146179021	61 kDa		4	

Probability Legend: over 95% | 80% to 94% | 50% to 79% | 20% to 49% | 0% to 19%

#	Visible?	Starred?	Bio View: Identified Proteins (1837)	Accession Number	Molecular Weight	Protein Grouping Ambiguity	01 Control	02 Sample
1407	☑	☆	hypothetical protein TTHERM_00998970 [Tetrahymena thermophila]	gi|146185086	97 kDa		4	
1408	☑	☆	hypothetical protein TTHERM_01299730 [Tetrahymena thermophila]	gi|229594156	101 kDa		4	
1409	☑	☆	Transketolase, C-terminal domain containing protein [Tetrahymena thermophila]	gi|146163490	139 kDa		4	
1410	☑	☆	Nucleoporin autopeptidase [Tetrahymena thermophila]	gi|118400911	235 kDa		4	
1411	☑	☆	Protein kinase domain containing protein [Tetrahymena thermophila]	gi|146165653	58 kDa		4	
1412	☑	☆	hypothetical protein TTHERM_00312690 [Tetrahymena thermophila]	gi|118349147	88 kDa		4	
1413	☑	☆	hypothetical protein TTHERM_00121030 [Tetrahymena thermophila]	gi|118355074	31 kDa		4	
1414	☑	☆	TPR Domain containing protein [Tetrahymena thermophila]	gi|118368634	92 kDa		4	
1415	☑	☆	hypothetical protein TTHERM_00348450 [Tetrahymena thermophila]	gi|118379669	72 kDa		4	
1416	☑	☆	EF hand family protein [Tetrahymena thermophila]	gi|118383583	95 kDa		4	
1417	☑	☆	hypothetical protein TTHERM_01141590 [Tetrahymena thermophila]	gi|118395566	111 kDa		4	
1418	☑	☆	hypothetical protein TTHERM_00675560 [Tetrahymena thermophila]	gi|146165431	130 kDa		4	
1419	☑	☆	hypothetical protein TTHERM_00290850 [Tetrahymena thermophila]	gi|146172955	182 kDa		4	
1420	☑	☆	hypothetical protein TTHERM_00492690 [Tetrahymena thermophila]	gi|118379993	66 kDa		4	
1421	☑	☆	hypothetical protein TTHERM_00684770 [Tetrahymena thermophila]	gi|118384050	22 kDa		4	
1422	☑	☆	EF hand family protein [Tetrahymena thermophila]	gi|146181449	17 kDa	★	4	
1423	☑	☆	ATPase, AAA family protein [Tetrahymena thermophila]	gi|118359475	78 kDa		4	
1424	☑	☆	hypothetical protein TTHERM_00213600 [Tetrahymena thermophila]	gi|146161442	40 kDa		4	
1425	☑	☆	von Willebrand factor type A domain containing protein [Tetrahymena thermophila]	gi|118357564	103 kDa		4	
1426	☑	☆	Mov34/MPN/PAD-1 family protein [Tetrahymena thermophila]	gi|118359916	24 kDa		4	
1427	☑	☆	hypothetical protein TTHERM_00317230 [Tetrahymena thermophila]	gi|118376444	16 kDa		4	
1428	☑	☆	hypothetical protein TTHERM_01276410 [Tetrahymena thermophila]	gi|118394990	56 kDa		4	
1429	☑	☆	hypothetical protein TTHERM_00643530 [Tetrahymena thermophila]	gi|118364300	71 kDa		4	
1430	☑	☆	ubiquitin-conjugating enzyme [Tetrahymena thermophila]	gi|146184801	17 kDa		4	
1431	☑	☆	MBOAT family protein [Tetrahymena thermophila]	gi|146165214	66 kDa		4	
1432	☑	☆	hypothetical protein TTHERM_00455610 [Tetrahymena thermophila]	gi|118382015	109 kDa		4	
1433	☑	☆	hypothetical protein TTHERM_00756430 [Tetrahymena thermophila]	gi|118398862	154 kDa		4	
1434	☑	☆	Anaphase promoting complex subunit 8 / cdc23 family protein [Tetrahymena thermophila]	gi|118356683	80 kDa		4	
1435	☑	☆	PX domain containing protein [Tetrahymena thermophila]	gi|118350506	103 kDa		4	
1436	☑	☆	hypothetical protein TTHERM_00077270 [Tetrahymena thermophila]	gi|118364894	36 kDa		4	
1437	☑	☆	conserved hypothetical protein [Tetrahymena thermophila]	gi|118373646	72 kDa		4	
1438	☑	☆	hypothetical protein TTHERM_00112410 [Tetrahymena thermophila]	gi|118354750	65 kDa		4	
1439	☑	☆	hypothetical protein TTHERM_00037020 [Tetrahymena thermophila]	gi|118346537	105 kDa		4	
1440	☑	☆	Leucine Rich Repeat family protein [Tetrahymena thermophila]	gi|146161146	158 kDa		4	
1441	☑	☆	hypothetical protein TTHERM_00086850 [Tetrahymena thermophila]	gi|118358984	33 kDa		4	
1442	☑	☆	hypothetical protein TTHERM_00139700 [Tetrahymena thermophila]	gi|118373316	88 kDa		4	
1443	☑	☆	Structure-specific recognition protein [Tetrahymena thermophila]	gi|146165332	50 kDa		4	

Probability Legend: over 95% | 80% to 94% | 50% to 79% | 20% to 49% | 0% to 19%

#	Visible?	Starred?	Bio View: Identified Proteins (1837)	Accession Number	Molecular Weight	Protein Grouping Ambiguity	01 Control	02 Sample
1444	☑	☆	Ubiquitin carboxyl-terminal hydrolase family protein [Tetrahymena thermophila]	gi|118399511	511 kDa		4	
1445	☑	☆	Zinc finger, ZZ type family protein [Tetrahymena thermophila]	gi|118380071	79 kDa		4	
1446	☑	☆	hypothetical protein TTHERM_00856680 [Tetrahymena thermophila]	gi|229595877	37 kDa		4	
1447	☑	☆	Sec7 domain containing protein [Tetrahymena thermophila]	gi|118389124 (+1)	238 kDa		4	
1448	☑	☆	Tubulin/FtsZ family, GTPase domain containing protein [Tetrahymena thermophila]	gi|118365142 (+1)	53 kDa		4	
1449	☑	☆	hypothetical protein TTHERM_00419990 [Tetrahymena thermophila]	gi|118401869	167 kDa		4	
1450	☑	☆	hypothetical protein TTHERM_00070730 [Tetrahymena thermophila]	gi|118348672	632 kDa		4	
1451	☑	☆	Protein transport protein SEC61 gamma-1 subunit, putative [Tetrahymena thermophila]	gi|118379524	8 kDa		4	
1452	☑	☆	hypothetical protein TTHERM_00558180 [Tetrahymena thermophila]	gi|118378411	130 kDa		4	
1453	☑	☆	hypothetical protein TTHERM_00276060 [Tetrahymena thermophila]	gi|118365631	115 kDa		4	
1454	☑	☆	hypothetical protein TTHERM_00220670 [Tetrahymena thermophila]	gi|118374903	162 kDa		4	
1455	☑	☆	hypothetical protein TTHERM_01108490 [Tetrahymena thermophila]	gi|118395728	50 kDa		4	
1456	☑	☆	hypothetical protein TTHERM_00355110 [Tetrahymena thermophila]	gi|146162935	164 kDa		4	
1457	☑	☆	hypothetical protein TTHERM_01326880 [Tetrahymena thermophila]	gi|118394782	137 kDa		4	
1458	☑	☆	Leucine Rich Repeat family protein [Tetrahymena thermophila]	gi|118349642	76 kDa	★	4	
1459	☑	☆	hypothetical protein TTHERM_00112650 [Tetrahymena thermophila]	gi|118354798	37 kDa		4	
1460	☑	☆	hypothetical protein TTHERM_00444420 [Tetrahymena thermophila]	gi|118380280	66 kDa		4	
1461	☑	☆	hypothetical protein TTHERM_00327070 [Tetrahymena thermophila]	gi|118386705	51 kDa		4	
1462	☑	☆	hypothetical protein TTHERM_00830510 [Tetrahymena thermophila]	gi|229596000	116 kDa	★	4	
1463	☑	☆	hypothetical protein TTHERM_00616600 [Tetrahymena thermophila]	gi|118383149	131 kDa		4	
1464	☑	☆	hypothetical protein TTHERM_01005060 [Tetrahymena thermophila]	gi|229595795	40 kDa		4	
1465	☑	☆	hypothetical protein TTHERM_00535450 [Tetrahymena thermophila]	gi|146181824	175 kDa		4	
1466	☑	☆	hypothetical protein TTHERM_00462860 [Tetrahymena thermophila]	gi|118371169	40 kDa		4	
1467	☑	☆	small GTP-binding protein domain containing protein [Tetrahymena thermophila]	gi|118365411	22 kDa		4	
1468	☑	☆	hypothetical protein TTHERM_00475210 [Tetrahymena thermophila]	gi|118381685	42 kDa		4	
1469	☑	☆	hypothetical protein TTHERM_00354760 [Tetrahymena thermophila]	gi|118354223	104 kDa		4	
1470	☑	☆	hypothetical protein TTHERM_00966510 [Tetrahymena thermophila]	gi|118351327	68 kDa	★	4	
1471	☑	☆	jmjC domain containing protein [Tetrahymena thermophila]	gi|146162156	123 kDa		3	
1472	☑	☆	LMBR1-like conserved region family protein [Tetrahymena thermophila]	gi|146179713	241 kDa		3	
1473	☑	☆	XYPPX repeat family protein [Tetrahymena thermophila]	gi|118361979	33 kDa		3	
1474	☑	☆	Zinc knuckle family protein [Tetrahymena thermophila]	gi|118401479	41 kDa		3	
1475	☑	☆	hypothetical protein TTHERM_00196150 [Tetrahymena thermophila]	gi|229595487	40 kDa		3	
1476	☑	☆	hypothetical protein TTHERM_00927080 [Tetrahymena thermophila]	gi|118397568	32 kDa		3	
1477	☑	☆	ABC transporter N-terminus family protein [Tetrahymena thermophila]	gi|118384191	82 kDa		3	
1478	☑	☆	dnaK protein [Tetrahymena thermophila]	gi|118401337 (+1)	102 kDa		3	
1479	☑	☆	hypothetical protein IMG5_146630 [Ichthyophthirius multifiliis]	gi|340503287	472 kDa	★	3	
1480	☑	☆	Protein kinase domain containing protein [Tetrahymena thermophila]	gi|118354381	72 kDa		3	

Probability Legend: over 95% | 80% to 94% | 50% to 79% | 20% to 49% | 0% to 19%

#	Visible?	Starred?	Bio View: Identified Proteins (1837)	Accession Number	Molecular Weight	Protein Grouping Ambiguity	01 Control	02 Sample
1481	☑	☆	hypothetical protein TTHERM_00696900 [Tetrahymena thermophila]	gi|146182936	44 kDa		3	
1482	☑	☆	Ubiquitin carboxyl-terminal hydrolase family protein [Tetrahymena thermophila]	gi|118401656	363 kDa		3	
1483	☑	☆	TPR Domain containing protein [Tetrahymena thermophila]	gi|118395872	102 kDa		3	
1484	☑	☆	hypothetical protein TTHERM_00686200 [Tetrahymena thermophila]	gi|118384138	164 kDa		3	
1485	☑	☆	EF hand family protein [Tetrahymena thermophila]	gi|118389122	18 kDa	★	3	
1486	☑	☆	hypothetical protein TTHERM_00170270 [Tetrahymena thermophila]	gi|118350817	56 kDa		3	
1487	☑	☆	TPR Domain containing protein [Tetrahymena thermophila]	gi|118352013	82 kDa		3	
1488	☑	☆	hypothetical protein TTHERM_00727670 [Tetrahymena thermophila]	gi|118378965	29 kDa		3	
1489	☑	☆	Ankyrin repeat protein [Tetrahymena thermophila]	gi|118376808	44 kDa		3	
1490	☑	☆	DnaJ domain containing protein [Tetrahymena thermophila]	gi|118401788	79 kDa		3	
1491	☑	☆	Rab-family small GTPase Rab11H [Tetrahymena thermophila]	gi|307777890	25 kDa		3	
1492	☑	☆	hypothetical protein TTHERM_00486070 [Tetrahymena thermophila]	gi|118400998	172 kDa		3	
1493	☑	☆	Importin-beta N-terminal domain containing protein [Tetrahymena thermophila]	gi|118358371	119 kDa		3	
1494	☑	☆	conserved hypothetical protein [Tetrahymena thermophila]	gi|229595327	44 kDa		3	
1495	☑	☆	hypothetical protein TTHERM_00993040 [Tetrahymena thermophila]	gi|229594203	44 kDa		3	
1496	☑	☆	hypothetical protein TTHERM_00049209 [Tetrahymena thermophila]	gi|146165345	120 kDa		3	
1497	☑	☆	conserved hypothetical protein [Tetrahymena thermophila]	gi|146182541	33 kDa		3	
1498	☑	☆	hypothetical protein TTHERM_00151720 [Tetrahymena thermophila]	gi|118377046	88 kDa		3	
1499	☑	☆	RNA binding motif protein [Tetrahymena thermophila]	gi|118381772	83 kDa		3	
1500	☑	☆	FAD binding domain containing protein [Tetrahymena thermophila]	gi|118348068	67 kDa		3	
1501	☑	☆	Viral A-type inclusion protein repeat containing protein [Tetrahymena thermophila]	gi|118371099	217 kDa		3	
1502	☑	☆	WD domain containing protein [Tetrahymena thermophila]	gi|146169504	39 kDa		3	
1503	☑	☆	hypothetical protein TTHERM_00193580 [Tetrahymena thermophila]	gi|118367803	42 kDa		3	
1504	☑	☆	hypothetical protein TTHERM_00189060 [Tetrahymena thermophila]	gi|229595559	37 kDa		3	
1505	☑	☆	Patatin-like phospholipase family protein [Tetrahymena thermophila]	gi|118362183	41 kDa		3	
1506	☑	☆	TBC domain containing protein [Tetrahymena thermophila]	gi|118363086	117 kDa		3	
1507	☑	☆	hypothetical protein TTHERM_00196220 [Tetrahymena thermophila]	gi|118368133	18 kDa		3	
1508	☑	☆	FF domain containing protein [Tetrahymena thermophila]	gi|118398780	90 kDa		3	
1509	☑	☆	Ras family protein [Tetrahymena thermophila]	gi|146184753	30 kDa		3	
1510	☑	☆	Bm44, putative [Tetrahymena thermophila]	gi|118350458	24 kDa		3	
1511	☑	☆	Beige/BEACH domain containing protein [Tetrahymena thermophila]	gi|118346022	524 kDa		3	
1512	☑	☆	hypothetical protein TTHERM_00181090 [Tetrahymena thermophila]	gi|118350995	47 kDa		3	
1513	☑	☆	Mov34/MPN/PAD-1 family protein [Tetrahymena thermophila]	gi|118363404	35 kDa		3	
1514	☑	☆	Ku70/Ku80 beta-barrel domain containing protein [Tetrahymena thermophila]	gi|118379947	87 kDa		3	
1515	☑	☆	hypothetical protein TTHERM_00713270 [Tetrahymena thermophila]	gi|118385500	62 kDa		3	
1516	☑	☆	hypothetical protein TTHERM_00092790 [Tetrahymena thermophila]	gi|146163998	21 kDa		3	
1517	☑	☆	DIRP family protein [Tetrahymena thermophila]	gi|146176546	74 kDa		3	

Probability Legend: over 95% | 80% to 94% | 50% to 79% | 20% to 49% | 0% to 19%

#	Visible?	Starred?	Bio View: Identified Proteins (1837)	Accession Number	Molecular Weight	Protein Grouping Ambiguity	01 Control	02 Sample
1518	☑	☆	hypothetical protein TTHERM_00469400 [Tetrahymena thermophila]	gi|146182738	20 kDa		3	
1519	☑	☆	hypothetical protein TTHERM_00697090 [Tetrahymena thermophila]	gi|146182939	116 kDa		3	
1520	☑	☆	zinc finger protein [Tetrahymena thermophila]	gi|146185727	51 kDa		3	
1521	☑	☆	TPR Domain containing protein [Tetrahymena thermophila]	gi|118396400	35 kDa		3	
1522	☑	☆	hypothetical protein TTHERM_00312320 [Tetrahymena thermophila]	gi|118349073	142 kDa		3	
1523	☑	☆	hypothetical protein TTHERM_00355570 [Tetrahymena thermophila]	gi|118354385	119 kDa		3	
1524	☑	☆	DNA repair protein RAD51 containing protein [Tetrahymena thermophila]	gi|118355624	36 kDa		3	
1525	☑	☆	Type III restriction enzyme, res subunit family protein [Tetrahymena thermophila]	gi|118358423	313 kDa		3	
1526	☑	☆	hypothetical protein TTHERM_00196380 [Tetrahymena thermophila]	gi|118368165	83 kDa		3	
1527	☑	☆	hypothetical protein TTHERM_00249650 [Tetrahymena thermophila]	gi|118371634	15 kDa		3	
1528	☑	☆	LEM3 (ligand-effect modulator 3) family protein [Tetrahymena thermophila]	gi|118386495	36 kDa		3	
1529	☑	☆	hypothetical protein TTHERM_01002720 [Tetrahymena thermophila]	gi|118397070	27 kDa		3	
1530	☑	☆	hypothetical protein TTHERM_00755930 [Tetrahymena thermophila]	gi|118398790	25 kDa		3	
1531	☑	☆	Glycosyl hydrolase family 85 protein [Tetrahymena thermophila]	gi|146161799	231 kDa		3	
1532	☑	☆	Calpain family cysteine protease containing protein [Tetrahymena thermophila]	gi|146165125	202 kDa		3	
1533	☑	☆	hypothetical protein TTHERM_00243960 [Tetrahymena thermophila]	gi|146181975	214 kDa		3	
1534	☑	☆	hypothetical protein TTHERM_00697290 [Tetrahymena thermophila]	gi|146182943	43 kDa		3	
1535	☑	☆	hypothetical protein TTHERM_00585510 [Tetrahymena thermophila]	gi|229594531	18 kDa		3	
1536	☑	☆	hypothetical protein TTHERM_01207630 [Tetrahymena thermophila]	gi|229594756	61 kDa		3	
1537	☑	☆	DnaJ domain containing protein [Tetrahymena thermophila]	gi|229595623	35 kDa		3	
1538	☑	☆	ARID/BRIGHT DNA binding domain containing protein [Tetrahymena thermophila]	gi|146181973	76 kDa		3	
1539	☑	☆	SWIB/MDM2 domain containing protein [Tetrahymena thermophila]	gi|118397681	64 kDa		3	
1540	☑	☆	cation channel family protein [Tetrahymena thermophila]	gi|118352466	402 kDa		3	
1541	☑	☆	hypothetical protein TTHERM_00471930 [Tetrahymena thermophila]	gi|118401513	97 kDa		3	
1542	☑	☆	hypothetical protein TTHERM_00857860 [Tetrahymena thermophila]	gi|118377500	59 kDa		3	
1543	☑	☆	methionyl-tRNA synthetase [Tetrahymena thermophila]	gi|118399643	63 kDa		3	
1544	☑	☆	hypothetical protein TTHERM_01087820 [Tetrahymena thermophila]	gi|118369339	194 kDa		3	
1545	☑	☆	Kinesin motor domain containing protein [Tetrahymena thermophila]	gi|118386141	125 kDa		3	
1546	☑	☆	hypothetical protein TTHERM_00301740 [Tetrahymena thermophila]	gi|118382892	28 kDa		3	
1547	☑	☆	ATPase, AAA family protein [Tetrahymena thermophila]	gi|146183034	47 kDa		3	
1548	☑	☆	Sec7 domain containing protein [Tetrahymena thermophila]	gi|146185202	215 kDa		3	
1549	☑	☆	hypothetical protein TTHERM_01262850 [Tetrahymena thermophila]	gi|118385838	116 kDa		3	
1550	☑	☆	Replication factor C subunit, putative [Tetrahymena thermophila]	gi|118353287	41 kDa		3	
1551	☑	☆	BRCT domain containing protein [Tetrahymena thermophila]	gi|118397505	129 kDa		3	
1552	☑	☆	hypothetical protein TTHERM_00047120 [Tetrahymena thermophila]	gi|118362912	40 kDa		3	
1553	☑	☆	hypothetical protein TTHERM_00895610 [Tetrahymena thermophila]	gi|118397760	23 kDa		3	
1554	☑	☆	hypothetical protein TTHERM_00601850 [Tetrahymena thermophila]	gi|118354515	87 kDa		3	

Probability Legend: over 95% | 80% to 94% | 50% to 79% | 20% to 49% | 0% to 19%

#	Visible?	Starred?	Bio View: Identified Proteins (1837)	Accession Number	Molecular Weight	Protein Grouping Ambiguity	01 Control	02 Sample
1555	☑	☆	hypothetical protein TTHERM_00398080 [Tetrahymena thermophila]	gi|229595935	25 kDa		3	
1556	☑	☆	hypothetical protein TTHERM_01040860 [Tetrahymena thermophila]	gi|118385727	69 kDa	★	3	
1557	☑	☆	Kinesin motor domain containing protein [Tetrahymena thermophila]	gi|146185978	123 kDa		3	
1558	☑	☆	hypothetical protein TTHERM_00540280 [Tetrahymena thermophila]	gi|118348960	66 kDa		3	
1559	☑	☆	Ras family protein [Tetrahymena thermophila]	gi|118356223	28 kDa	★	3	
1560	☑	☆	hypothetical protein TTHERM_00522420 [Tetrahymena thermophila]	gi|118362386	142 kDa		3	
1561	☑	☆	Zinc carboxypeptidase family protein [Tetrahymena thermophila]	gi|146183815	73 kDa		3	
1562	☑	☆	hypothetical protein TTHERM_00522740 [Tetrahymena thermophila]	gi|118362450	201 kDa		3	
1563	☑	☆	hypothetical protein TTHERM_01372820 [Tetrahymena thermophila]	gi|146184576	57 kDa		3	
1564	☑	☆	EF hand family protein [Tetrahymena thermophila]	gi|118396782	19 kDa		3	
1565	☑	☆	phospholipid-translocating P-type ATPase, flippase family protein [Tetrahymena thermophila]	gi|146180724	146 kDa		3	
1566	☑	☆	Dynein heavy chain family protein [Tetrahymena thermophila]	gi|118380021	548 kDa		3	
1567	☑	☆	hypothetical protein TTHERM_00138280 [Tetrahymena thermophila]	gi|146175267	95 kDa		3	
1568	☑	☆	Transketolase, pyridine binding domain containing protein [Tetrahymena thermophila]	gi|118367391	47 kDa		3	
1569	☑	☆	Ubiquitin carboxyl-terminal hydrolase family protein [Tetrahymena thermophila]	gi|146184643	111 kDa		3	
1570	☑	☆	DEAD/DEAH box helicase family protein [Tetrahymena thermophila]	gi|118372814	127 kDa		3	
1571	☑	☆	hypothetical protein TTHERM_00919620 [Tetrahymena thermophila]	gi|118389543	105 kDa		3	
1572	☑	☆	Mov34/MPN/PAD-1 family protein [Tetrahymena thermophila]	gi|118368363	36 kDa		3	
1573	☑	☆	Ser/Thr protein phosphatase family protein [Tetrahymena thermophila]	gi|146165300	22 kDa		3	
1574	☑	☆	calcium-translocating P-type ATPase, PMCA-type family protein [Tetrahymena thermophila]	gi|118383497	115 kDa		3	
1575	☑	☆	hypothetical protein TTHERM_00486439 [Tetrahymena thermophila]	gi|146185996	125 kDa		3	
1576	☑	☆	IQ calmodulin-binding motif family protein [Tetrahymena thermophila]	gi|118363418	273 kDa		3	
1577	☑	☆	hypothetical protein TTHERM_00388200 [Tetrahymena thermophila]	gi|118372255	82 kDa		3	
1578	☑	☆	EF hand family protein [Tetrahymena thermophila]	gi|146169367	107 kDa		3	
1579	☑	☆	MIZ zinc finger family protein [Tetrahymena thermophila]	gi|118354934	111 kDa		3	
1580	☑	☆	DNA polymerase family B containing protein [Tetrahymena thermophila]	gi|118400060	130 kDa		3	
1581	☑	☆	Eukaryotic aspartyl protease family protein [Tetrahymena thermophila]	gi|146168013	66 kDa		3	
1582	☑	☆	hypothetical protein TTHERM_00849220 [Tetrahymena thermophila]	gi|118372054	106 kDa		3	
1583	☑	☆	hypothetical protein TTHERM_00522030 [Tetrahymena thermophila]	gi|146164987	60 kDa		3	
1584	☑	☆	SET domain containing protein [Tetrahymena thermophila]	gi|146185998	242 kDa		3	
1585	☑	☆	hypothetical protein TTHERM_00196250 [Tetrahymena thermophila]	gi|146169781	28 kDa		3	
1586	☑	☆	CAF1 family ribonuclease containing protein [Tetrahymena thermophila]	gi|229593727	149 kDa		3	
1587	☑	☆	Ymf62 [Tetrahymena malaccensis]	gi|114329852 (+1)	31 kDa		3	
1588	☑	☆	hypothetical protein TTHERM_01164060 [Tetrahymena thermophila]	gi|146184747	158 kDa		3	
1589	☑	☆	hypothetical protein TTHERM_00780680 [Tetrahymena thermophila]	gi|118386207	80 kDa		3	
1590	☑	☆	hypothetical protein TTHERM_00185490 [Tetrahymena thermophila]	gi|118351083	168 kDa		3	
1591	☑	☆	hypothetical protein TTHERM_00046740 [Tetrahymena thermophila]	gi|118363198	188 kDa		3	

Probability Legend: over 95% | 80% to 94% | 50% to 79% | 20% to 49% | 0% to 19%

#	Visible?	Starred?	Bio View: Identified Proteins (1837)	Accession Number	Molecular Weight	Protein Grouping Ambiguity	01 Control	02 Sample
1592	☑	☆	RecName: Full=Histone H3.3; AltName: Full=H3.2; AltName: Full=Minor histone H3 variant; AltName: Full=hv2	gi|122066 (+1)	16 kDa	★	2	
1593	☑	☆	RNA binding protein [Tetrahymena thermophila]	gi|118379043	45 kDa		2	
1594	☑	☆	GRAM domain containing protein [Tetrahymena thermophila]	gi|118366431	196 kDa	★	2	
1595	☑	☆	hypothetical protein TTHERM_00812780 [Tetrahymena thermophila]	gi|146162057	10 kDa		2	
1596	☑	☆	hypothetical protein TTHERM_00459310 [Tetrahymena thermophila]	gi|146182264	33 kDa		2	
1597	☑	☆	hypothetical protein TTHERM_00865100 [Tetrahymena thermophila]	gi|118387277	45 kDa		2	
1598	☑	☆	hypothetical protein TTHERM_00420160 [Tetrahymena thermophila]	gi|118401903	20 kDa		2	
1599	☑	☆	phosphatidylserine decarboxylase family protein [Tetrahymena thermophila]	gi|118377592	121 kDa		2	
1600	☑	☆	Leucine Rich Repeat family protein [Tetrahymena thermophila]	gi|118348334	182 kDa		2	
1601	☑	☆	EF hand family protein [Tetrahymena thermophila]	gi|118399680	424 kDa		2	
1602	☑	☆	hypothetical protein TTHERM_00535480 [Tetrahymena thermophila]	gi|118380587	23 kDa		2	
1603	☑	☆	hypothetical protein TTHERM_00133730 [Tetrahymena thermophila]	gi|146175865	40 kDa		2	
1604	☑	☆	hypothetical protein TTHERM_00775940 [Tetrahymena thermophila]	gi|118376091	50 kDa		2	
1605	☑	☆	hypothetical protein TTHERM_00145910 [Tetrahymena thermophila]	gi|118355942	29 kDa		2	
1606	☑	☆	glycosyl transferase, group 1 family protein [Tetrahymena thermophila]	gi|118368712	47 kDa		2	
1607	☑	☆	hypothetical protein TTHERM_00446430 [Tetrahymena thermophila]	gi|118380484	105 kDa		2	
1608	☑	☆	ATPase, AAA family protein [Tetrahymena thermophila]	gi|118352130	76 kDa		2	
1609	☑	☆	hypothetical protein TTHERM_00670600 [Tetrahymena thermophila]	gi|146183520	196 kDa		2	
1610	☑	☆	Ras family protein [Tetrahymena thermophila]	gi|118369861	23 kDa	★	2	
1611	☑	☆	hypothetical protein TTHERM_00348390 [Tetrahymena thermophila]	gi|118379657	37 kDa		2	
1612	☑	☆	Protein kinase domain containing protein [Tetrahymena thermophila]	gi|118366305	68 kDa		2	
1613	☑	☆	hypothetical protein TTHERM_00929580 [Tetrahymena thermophila]	gi|146183114	205 kDa		2	
1614	☑	☆	Peptidase C13 family protein [Tetrahymena thermophila]	gi|146184083	39 kDa		2	
1615	☑	☆	zinc finger protein [Tetrahymena thermophila]	gi|118387073	107 kDa		2	
1616	☑	☆	hypothetical protein TTHERM_00895820 [Tetrahymena thermophila]	gi|118397802	56 kDa		2	
1617	☑	☆	hypothetical protein TTHERM_01043360 [Tetrahymena thermophila]	gi|118396688	34 kDa		2	
1618	☑	☆	hypothetical protein TTHERM_00075680 [Tetrahymena thermophila]	gi|118364774	91 kDa		2	
1619	☑	☆	Phosphatidylinositol 3- and 4-kinase family protein [Tetrahymena thermophila]	gi|118347218	511 kDa		2	
1620	☑	☆	hypothetical protein TTHERM_00752160 [Tetrahymena thermophila]	gi|118370227	34 kDa		2	
1621	☑	☆	nucleic acid helicase, putative [Tetrahymena thermophila]	gi|146163979	208 kDa		2	
1622	☑	☆	hypothetical protein TTHERM_00584930 [Tetrahymena thermophila]	gi|229594521	49 kDa		2	
1623	☑	☆	FHA domain containing protein [Tetrahymena thermophila]	gi|118367274	91 kDa		2	
1624	☑	☆	hypothetical protein TTHERM_00592830 [Tetrahymena thermophila]	gi|118356777	59 kDa		2	
1625	☑	☆	SWIRM domain containing protein [Tetrahymena thermophila]	gi|118400536	115 kDa		2	
1626	☑	☆	hypothetical protein TTHERM_00599960 [Tetrahymena thermophila]	gi|118400295	96 kDa		2	
1627	☑	☆	dehydrogenase, isocitrate/isopropylmalate family protein [Tetrahymena thermophila]	gi|146180442	53 kDa		2	
1628	☑	☆	Adaptor complexes medium subunit family protein [Tetrahymena thermophila]	gi|118389248	40 kDa		2	

#	Visible?	Starred?	Bio View: Identified Proteins (1837)	Accession Number	Molecular Weight	Protein Grouping Ambiguity	01 Control	02 Sample
1629	☑	☆	Elongation factor Tu GTP binding domain containing protein [Tetrahymena thermophila]	gi|118361328	174 kDa		2	
1630	☑	☆	hypothetical protein TTHERM_00079330 [Tetrahymena thermophila]	gi|118365106	120 kDa		2	
1631	☑	☆	hypothetical protein TTHERM_00530590 [Tetrahymena thermophila]	gi|118400885	23 kDa		2	
1632	☑	☆	Ras family protein [Tetrahymena thermophila]	gi|118386344	26 kDa		2	
1633	☑	☆	hypothetical protein TTHERM_00219320 [Tetrahymena thermophila]	gi|118374833	40 kDa		2	
1634	☑	☆	Viral A-type inclusion protein repeat containing protein [Tetrahymena thermophila]	gi|118381947	257 kDa	★	2	
1635	☑	☆	Protein kinase domain containing protein [Tetrahymena thermophila]	gi|118396389	36 kDa		2	
1636	☑	☆	Guanylate-binding protein, N-terminal domain containing protein [Tetrahymena thermophila]	gi|118352484	132 kDa		2	
1637	☑	☆	MHCK/EF2 kinase domain family protein [Tetrahymena thermophila]	gi|118388807	119 kDa		2	
1638	☑	☆	hypothetical protein TTHERM_00339710 [Tetrahymena thermophila]	gi|118368858	52 kDa		2	
1639	☑	☆	hypothetical protein TTHERM_00823720 [Tetrahymena thermophila]	gi|118398290	18 kDa		2	
1640	☑	☆	hypothetical protein TTHERM_00614670 [Tetrahymena thermophila]	gi|229594257	17 kDa		2	
1641	☑	☆	Hsp90 protein [Tetrahymena thermophila]	gi|118365244	82 kDa		2	
1642	☑	☆	hypothetical protein TTHERM_00476740 [Tetrahymena thermophila]	gi|118368349	24 kDa		2	
1643	☑	☆	hypothetical protein TTHERM_00835040 [Tetrahymena thermophila]	gi|146185210	44 kDa		2	
1644	☑	☆	Phosphopantetheine attachment site family protein [Tetrahymena thermophila]	gi|229595797	16 kDa		2	
1645	☑	☆	hypothetical protein TTHERM_00188600 [Tetrahymena thermophila]	gi|118366641	199 kDa		2	
1646	☑	☆	Amidase family protein [Tetrahymena thermophila]	gi|118347746	69 kDa	★	2	
1647	☑	☆	hypothetical protein TTHERM_00312730 [Tetrahymena thermophila]	gi|118349155	139 kDa		2	
1648	☑	☆	hypothetical protein TTHERM_00392900 [Tetrahymena thermophila]	gi|118357233	25 kDa		2	
1649	☑	☆	Ubiquitin carboxyl-terminal hydrolase family protein [Tetrahymena thermophila]	gi|118360308	293 kDa		2	
1650	☑	☆	hypothetical protein TTHERM_00653790 [Tetrahymena thermophila]	gi|118361405	102 kDa		2	
1651	☑	☆	hypothetical protein TTHERM_00820660 [Tetrahymena thermophila]	gi|118365910	73 kDa	★	2	
1652	☑	☆	hypothetical protein TTHERM_00285610 [Tetrahymena thermophila]	gi|118370750	41 kDa		2	
1653	☑	☆	hypothetical protein TTHERM_00850590 [Tetrahymena thermophila]	gi|118372130	72 kDa		2	
1654	☑	☆	Ubiquitin-conjugating enzyme family protein [Tetrahymena thermophila]	gi|118374645	66 kDa		2	
1655	☑	☆	hypothetical protein TTHERM_00218470 [Tetrahymena thermophila]	gi|118374663	216 kDa		2	
1656	☑	☆	Phosphatidylinositol-4-phosphate 5-Kinase family protein [Tetrahymena thermophila]	gi|118375978	101 kDa		2	
1657	☑	☆	hypothetical protein TTHERM_00535410 [Tetrahymena thermophila]	gi|118380573	32 kDa		2	
1658	☑	☆	ATP-dependent metalloprotease FtsH family protein [Tetrahymena thermophila]	gi|118382640	102 kDa	★	2	
1659	☑	☆	von Willebrand factor type A domain containing protein [Tetrahymena thermophila]	gi|118384116	73 kDa		2	
1660	☑	☆	calcium-translocating P-type ATPase, PMCA-type family protein [Tetrahymena thermophila]	gi|118386173	141 kDa		2	
1661	☑	☆	merozoite surface protein 4/5, putative [Tetrahymena thermophila]	gi|118388676	17 kDa		2	
1662	☑	☆	hypothetical protein TTHERM_00149839 [Tetrahymena thermophila]	gi|146180860	19 kDa		2	
1663	☑	☆	hypothetical protein TTHERM_00923030 [Tetrahymena thermophila]	gi|229594900	29 kDa		2	
1664	☑	☆	Rab-family small GTPase Rab6A [Tetrahymena thermophila]	gi|307777798	26 kDa	★	2	
1665	☑	☆	hypothetical protein TTHERM_00522240 [Tetrahymena thermophila]	gi|118362350	45 kDa		2	

Probability Legend: over 95% / 80% to 94% / 50% to 79% / 20% to 49% / 0% to 19%

Probability Legend: over 95% | 80% to 94% | 50% to 79% | 20% to 49% | 0% to 19%

#	Visible?	Starred?	Bio View: Identified Proteins (1837)	Accession Number	Molecular Weight	Protein Grouping Ambiguity	01 Control	02 Sample
1666	☑	☆	DegT/DnrJ/EryC1/StrS aminotransferase family protein [Tetrahymena thermophila]	gi|118359710	50 kDa		2	
1667	☑	☆	hypothetical protein TTHERM_00549510 [Tetrahymena thermophila]	gi|118345738	100 kDa		2	
1668	☑	☆	hypothetical protein TTHERM_00833840 [Tetrahymena thermophila]	gi|146164628	35 kDa		2	
1669	☑	☆	Bromodomain containing protein [Tetrahymena thermophila]	gi|118364670	102 kDa		2	
1670	☑	☆	TPR Domain containing protein [Tetrahymena thermophila]	gi|146165308	50 kDa	★	2	
1671	☑	☆	hypothetical protein TTHERM_00657230 [Tetrahymena thermophila]	gi|118381726	13 kDa		2	
1672	☑	☆	2Fe-2S iron-sulfur cluster binding domain containing protein [Tetrahymena thermophila]	gi|118353159	20 kDa		2	
1673	☑	☆	hypothetical protein TTHERM_00841210 [Tetrahymena thermophila]	gi|118388278	28 kDa		2	
1674	☑	☆	cation diffusion facilitator family transporter containing protein [Tetrahymena thermophila]	gi|118389630	74 kDa		2	
1675	☑	☆	WGR domain containing protein [Tetrahymena thermophila]	gi|146183662	92 kDa		2	
1676	☑	☆	hypothetical protein TTHERM_00577130 [Tetrahymena thermophila]	gi|118352260	92 kDa		2	
1677	☑	☆	hypothetical protein TTHERM_00370800 [Tetrahymena thermophila]	gi|118352478	58 kDa		2	
1678	☑	☆	U-box domain containing protein [Tetrahymena thermophila]	gi|118352979	140 kDa		2	
1679	☑	☆	Dynamin central region family protein [Tetrahymena thermophila]	gi|118353117	85 kDa		2	
1680	☑	☆	hypothetical protein TTHERM_00140960 [Tetrahymena thermophila]	gi|118355548	74 kDa		2	
1681	☑	☆	PX domain containing protein [Tetrahymena thermophila]	gi|118355670	104 kDa		2	
1682	☑	☆	probable Mg2+-specific channel-like exchanger, putative [Tetrahymena thermophila]	gi|118355842	72 kDa		2	
1683	☑	☆	DnaJ domain containing protein [Tetrahymena thermophila]	gi|118358098	23 kDa		2	
1684	☑	☆	ABC transporter family protein [Tetrahymena thermophila]	gi|118359327	165 kDa		2	
1685	☑	☆	EF hand family protein [Tetrahymena thermophila]	gi|118362802	713 kDa		2	
1686	☑	☆	hypothetical protein TTHERM_00046490 [Tetrahymena thermophila]	gi|118362994	117 kDa		2	
1687	☑	☆	hypothetical protein TTHERM_00046840 [Tetrahymena thermophila]	gi|118363358	64 kDa		2	
1688	☑	☆	Protein kinase domain containing protein [Tetrahymena thermophila]	gi|118364706	219 kDa		2	
1689	☑	☆	TPR Domain containing protein [Tetrahymena thermophila]	gi|118365798	114 kDa		2	
1690	☑	☆	Type III restriction enzyme, res subunit family protein [Tetrahymena thermophila]	gi|118367847	254 kDa		2	
1691	☑	☆	hypothetical protein TTHERM_00196160 [Tetrahymena thermophila]	gi|118368121	91 kDa		2	
1692	☑	☆	hypothetical protein TTHERM_00283370 [Tetrahymena thermophila]	gi|118369981	100 kDa		2	
1693	☑	☆	hypothetical protein TTHERM_00753570 [Tetrahymena thermophila]	gi|118370301	14 kDa		2	
1694	☑	☆	hypothetical protein TTHERM_00257160 [Tetrahymena thermophila]	gi|118371946	131 kDa		2	
1695	☑	☆	EF hand family protein [Tetrahymena thermophila]	gi|118372128	21 kDa		2	
1696	☑	☆	small GTP-binding protein domain containing protein [Tetrahymena thermophila]	gi|118375516	22 kDa		2	
1697	☑	☆	hypothetical protein TTHERM_00630490 [Tetrahymena thermophila]	gi|118379212	72 kDa		2	
1698	☑	☆	hypothetical protein TTHERM_00732810 [Tetrahymena thermophila]	gi|118381034	387 kDa		2	
1699	☑	☆	hypothetical protein TTHERM_00475200 [Tetrahymena thermophila]	gi|118381683	29 kDa		2	
1700	☑	☆	Protein kinase domain containing protein [Tetrahymena thermophila]	gi|118382818	63 kDa		2	
1701	☑	☆	hypothetical protein TTHERM_00242330 [Tetrahymena thermophila]	gi|118383603	20 kDa		2	
1702	☑	☆	hypothetical protein TTHERM_00696940 [Tetrahymena thermophila]	gi|118384935	105 kDa		2	

Probability Legend
- over 95%
- 80% to 94%
- 50% to 79%
- 20% to 49%
- 0% to 19%

#	Visible?	Starred?	Bio View: Identified Proteins (1837)	Accession Number	Molecular Weight	Protein Grouping Ambiguity	01 Control	02 Sample
1703	✓	☆	EF hand family protein [Tetrahymena thermophila]	gi|118386517	19 kDa		2	
1704	✓	☆	TPR Domain containing protein [Tetrahymena thermophila]	gi|118386759	94 kDa		2	
1705	✓	☆	CHCH domain containing protein [Tetrahymena thermophila]	gi|118386839	28 kDa		2	
1706	✓	☆	hypothetical protein TTHERM_00841280 [Tetrahymena thermophila]	gi|118388292	53 kDa		2	
1707	✓	☆	HSF-type DNA-binding domain containing protein [Tetrahymena thermophila]	gi|118389216	74 kDa		2	
1708	✓	☆	Importin-beta N-terminal domain containing protein [Tetrahymena thermophila]	gi|118390251	108 kDa		2	
1709	✓	☆	hypothetical protein TTHERM_01151590 [Tetrahymena thermophila]	gi|118395484	129 kDa		2	
1710	✓	☆	hypothetical protein TTHERM_01099230 [Tetrahymena thermophila]	gi|118395990	222 kDa		2	
1711	✓	☆	hypothetical protein TTHERM_01016190 [Tetrahymena thermophila]	gi|118396885	162 kDa		2	
1712	✓	☆	Papain family cysteine protease containing protein [Tetrahymena thermophila]	gi|118401108	41 kDa		2	
1713	✓	☆	IBR domain containing protein [Tetrahymena thermophila]	gi|118401708	70 kDa		2	
1714	✓	☆	phosphatidate cytidylyltransferase family protein [Tetrahymena thermophila]	gi|146161650	51 kDa	★	2	
1715	✓	☆	hypothetical protein TTHERM_00357110 [Tetrahymena thermophila]	gi|146162984	48 kDa		2	
1716	✓	☆	SnoRNA binding domain containing protein [Tetrahymena thermophila]	gi|146163076	61 kDa		2	
1717	✓	☆	hypothetical protein TTHERM_00145310 [Tetrahymena thermophila]	gi|146163292	84 kDa		2	
1718	✓	☆	hypothetical protein TTHERM_00146330 [Tetrahymena thermophila]	gi|146163349	136 kDa		2	
1719	✓	☆	PHD-finger family protein [Tetrahymena thermophila]	gi|146165227	149 kDa		2	
1720	✓	☆	hypothetical protein TTHERM_00378600 [Tetrahymena thermophila]	gi|146165579	27 kDa		2	
1721	✓	☆	kinase domain containing protein [Tetrahymena thermophila]	gi|146170354	87 kDa		2	
1722	✓	☆	UBA/TS-N domain containing protein [Tetrahymena thermophila]	gi|146170451	50 kDa		2	
1723	✓	☆	hypothetical protein TTHERM_00335750 [Tetrahymena thermophila]	gi|146170462	131 kDa		2	
1724	✓	☆	hypothetical protein TTHERM_00219110 [Tetrahymena thermophila]	gi|146179379	25 kDa		2	
1725	✓	☆	small GTP-binding protein domain containing protein [Tetrahymena thermophila]	gi|146182464	23 kDa		2	
1726	✓	☆	DnaJ domain containing protein [Tetrahymena thermophila]	gi|146182547	39 kDa		2	
1727	✓	☆	hypothetical protein TTHERM_01099210 [Tetrahymena thermophila]	gi|146184864	27 kDa		2	
1728	✓	☆	hypothetical protein TTHERM_01026420 [Tetrahymena thermophila]	gi|146185023	55 kDa		2	
1729	✓	☆	AT hook motif family protein [Tetrahymena thermophila]	gi|229594269	91 kDa		2	
1730	✓	☆	Acyltransferase family protein [Tetrahymena thermophila]	gi|229594505	82 kDa		2	
1731	✓	☆	hypothetical protein TTHERM_00196180 [Tetrahymena thermophila]	gi|229595489	54 kDa		2	
1732	✓	☆	hypothetical protein TTHERM_00784250 [Tetrahymena thermophila]	gi|229596306	67 kDa		2	
1733	✓	☆	hypothetical protein TTHERM_00001289 [Tetrahymena thermophila]	gi|229596602	90 kDa		2	
1734	✓	☆	sperm associated antigen 6, putative [Ichthyophthirius multifiliis]	gi|340501712 (+1)	55 kDa		2	
1735	✓	☆	protein disulfide-isomerase domain containing protein [Tetrahymena thermophila]	gi|118354146	47 kDa		2	
1736	✓	☆	Peptidase family M48 containing protein [Tetrahymena thermophila]	gi|146184827	56 kDa		2	
1737	✓	☆	DHHC zinc finger domain containing protein [Tetrahymena thermophila]	gi|146180605	44 kDa		2	
1738	✓	☆	hypothetical protein TTHERM_00558050 [Tetrahymena thermophila]	gi|146181236	41 kDa		2	
1739	✓	☆	Ras family protein [Tetrahymena thermophila]	gi|118376340	26 kDa	★	2	

Probability Legend: over 95% | 80% to 94% | 50% to 79% | 20% to 49% | 0% to 19%

#	Visible?	Starred?	Bio View: Identified Proteins (1837)	Accession Number	Molecular Weight	Protein Grouping Ambiguity	01 Control	02 Sample
1740	☑	☆	hypothetical protein TTHERM_00390180 [Tetrahymena thermophila]	gi|118372453	71 kDa		2	
1741	☑	☆	Armadillo/beta-catenin repeat family protein [Tetrahymena thermophila]	gi|118359892 (+1)	61 kDa		2	
1742	☑	☆	hypothetical protein TTHERM_00133700 [Tetrahymena thermophila]	gi|118372910	39 kDa		2	
1743	☑	☆	hypothetical protein TTHERM_00048900 [Tetrahymena thermophila]	gi|118362846	169 kDa		2	
1744	☑	☆	small GTP-binding protein domain containing protein [Tetrahymena thermophila]	gi|118354199	26 kDa		2	
1745	☑	☆	Ubiquitin carboxyl-terminal hydrolase family protein [Tetrahymena thermophila]	gi|118381651	150 kDa		2	
1746	☑	☆	conserved hypothetical protein [Tetrahymena thermophila]	gi|118368173	165 kDa		2	
1747	☑	☆	SLEI family protein [Tetrahymena thermophila]	gi|146184699	273 kDa	★	2	
1748	☑	☆	Synaptobrevin family protein [Tetrahymena thermophila]	gi|118381176	26 kDa		2	
1749	☑	☆	Fatty acid desaturase family protein [Tetrahymena thermophila]	gi|118384446	42 kDa		2	
1750	☑	☆	Fatty acid desaturase family protein [Tetrahymena thermophila]	gi|118368588	43 kDa		2	
1751	☑	☆	hypothetical protein TTHERM_00456770 [Tetrahymena thermophila]	gi|118382049	67 kDa		2	
1752	☑	☆	Adenylate and Guanylate cyclase catalytic domain containing protein [Tetrahymena thermophila]	gi|118379575	311 kDa		2	
1753	☑	☆	cyclic nucleotide-binding domain containing protein [Tetrahymena thermophila]	gi|118362826	94 kDa	★	2	
1754	☑	☆	hypothetical protein TTHERM_00831550 [Tetrahymena thermophila]	gi|118360858	71 kDa	★	2	
1755	☑	☆	hypothetical protein TTHERM_00784270 [Tetrahymena thermophila]	gi|118356458	76 kDa		2	
1756	☑	☆	hypothetical protein TTHERM_01113060 [Tetrahymena thermophila]	gi|118385625	126 kDa		2	
1757	☑	☆	nitroreductase family protein [Tetrahymena thermophila]	gi|118401170	34 kDa		2	
1758	☑	☆	EF hand family protein [Tetrahymena thermophila]	gi|118380023	62 kDa		2	
1759	☑	☆	hypothetical protein TTHERM_00475400 [Tetrahymena thermophila]	gi|118381723	32 kDa		2	
1760	☑	☆	hypothetical protein TTHERM_00257170 [Tetrahymena thermophila]	gi|118371948	78 kDa		2	
1761	☑	☆	hypothetical protein TTHERM_00459230 [Tetrahymena thermophila]	gi|118382143	40 kDa		2	
1762	☑	☆	hypothetical protein TTHERM_00535670 [Tetrahymena thermophila]	gi|146181850	115 kDa		2	
1763	☑	☆	hypothetical protein TTHERM_00703480 [Tetrahymena thermophila]	gi|118399388	9 kDa		2	
1764	☑	☆	Papain family cysteine protease containing protein [Tetrahymena thermophila]	gi|118379711	42 kDa		2	
1765	☑	☆	hypothetical protein TTHERM_00349060 [Tetrahymena thermophila]	gi|118379791	17 kDa		2	
1766	☑	☆	hypothetical protein TTHERM_00433410 [Tetrahymena thermophila]	gi|118356219	61 kDa		2	
1767	☑	☆	hypothetical protein TTHERM_00283130 [Tetrahymena thermophila]	gi|118369933	90 kDa		2	
1768	☑	☆	hypothetical protein TTHERM_00633350 [Tetrahymena thermophila]	gi|118353615	21 kDa		2	
1769	☑	☆	hypothetical protein TTHERM_00143670 [Tetrahymena thermophila]	gi|146163248	35 kDa		2	
1770	☑	☆	oxidoreductase, short chain dehydrogenase/reductase family protein [Tetrahymena thermophila]	gi|118371377	37 kDa		2	
1771	☑	☆	RNA 2'-phosphotransferase, Tpt1 / KptA family protein [Tetrahymena thermophila]	gi|118362535	28 kDa		2	
1772	☑	☆	Kelch motif family protein [Tetrahymena thermophila]	gi|118372620	76 kDa		2	
1773	☑	☆	Kinesin motor domain containing protein [Tetrahymena thermophila]	gi|118378274	83 kDa		2	
1774	☑	☆	Protein kinase domain containing protein [Tetrahymena thermophila]	gi|146167965	62 kDa		2	
1775	☑	☆	Leucine Rich Repeat family protein [Tetrahymena thermophila]	gi|118358056	49 kDa		2	
1776	☑	☆	alveolin 2 [Tetrahymena thermophila]	gi|225675561	68 kDa		2	

Probability Legend: over 95% | 80% to 94% | 50% to 79% | 20% to 49% | 0% to 19%

#	Visible?	Starred?	Bio View: Identified Proteins (1837)	Accession Number	Molecular Weight	Protein Grouping Ambiguity	01 Control	02 Sample
1777	☑	☆	dynein light chain 8 [Tetrahymena thermophila]	gi|94541078	10 kDa		2	
1778	☑	☆	hypothetical protein TTHERM_00752080 [Tetrahymena thermophila]	gi|118370211	87 kDa		2	
1779	☑	☆	EF hand family protein [Tetrahymena thermophila]	gi|229594989	27 kDa		2	
1780	☑	☆	hypothetical protein TTHERM_00378800 [Tetrahymena thermophila]	gi|118364479	157 kDa		2	
1781	☑	☆	hypothetical protein TTHERM_01528500 [Tetrahymena thermophila]	gi|118394045	25 kDa		2	
1782	☑	☆	hypothetical protein TTHERM_00393040 [Tetrahymena thermophila]	gi|146163589	114 kDa		2	
1783	☑	☆	Dynein heavy chain family protein [Tetrahymena thermophila]	gi|118389527	656 kDa	★	2	
1784	☑	☆	hypothetical protein TTHERM_01001540 [Tetrahymena thermophila]	gi|118388815	34 kDa		2	
1785	☑	☆	hypothetical protein TTHERM_00196240 [Tetrahymena thermophila]	gi|118368137	56 kDa		2	
1786	☑	☆	hypothetical protein TTHERM_00310170 [Tetrahymena thermophila]	gi|118375819	77 kDa		2	
1787	☑	☆	hypothetical protein TTHERM_00121000 [Tetrahymena thermophila]	gi|118355068	38 kDa		2	
1788	☑	☆	hypothetical protein TTHERM_00218690 [Tetrahymena thermophila]	gi|118374707	32 kDa		2	
1789	☑	☆	hypothetical protein TTHERM_00105410 [Tetrahymena thermophila]	gi|118358086	47 kDa		2	
1790	☑	☆	hypothetical protein TTHERM_00715980 [Tetrahymena thermophila]	gi|118399259	90 kDa		2	
1791	☑	☆	hypothetical protein TTHERM_00633390 [Tetrahymena thermophila]	gi|118353623	195 kDa		2	
1792	☑	☆	Aspartyl/Asparaginyl beta-hydroxylase family protein [Tetrahymena thermophila]	gi|118395184	191 kDa		2	
1793	☑	☆	hypothetical protein TTHERM_01055640 [Tetrahymena thermophila]	gi|118388870	308 kDa		2	
1794	☑	☆	heat shock protein [Euplotes eurystomus]	gi|1209312	72 kDa	★	2	
1795	☑	☆	hypothetical protein TTHERM_01014770 [Tetrahymena thermophila]	gi|229594581	59 kDa		2	
1796	☑	☆	cation channel family protein [Tetrahymena thermophila]	gi|118360336	416 kDa		2	
1797	☑	☆	hypothetical protein TTHERM_00773530 [Tetrahymena thermophila]	gi|118398556	211 kDa		2	
1798	☑	☆	hypothetical protein TTHERM_00190658 [Tetrahymena thermophila]	gi|146168022	99 kDa		2	
1799	☑	☆	hypothetical protein TTHERM_00196280 [Tetrahymena thermophila]	gi|118368145	67 kDa		2	
1800	☑	☆	hypothetical protein TTHERM_00312160 [Tetrahymena thermophila]	gi|118349043	36 kDa		2	
1801	☑	☆	Glutathione peroxidase family protein [Tetrahymena thermophila]	gi|118369873	255 kDa		2	
1802	☑	☆	Ras family protein [Tetrahymena thermophila]	gi|118385741	25 kDa		2	
1803	☑	☆	hypothetical protein TTHERM_00323030 [Tetrahymena thermophila]	gi|118359529	119 kDa		2	
1804	☑	☆	hypothetical protein TTHERM_00399200 [Tetrahymena thermophila]	gi|229595937	37 kDa		2	
1805	☑	☆	hypothetical protein TTHERM_00294820 [Tetrahymena thermophila]	gi|118359752	48 kDa		2	
1806	☑	☆	hypothetical protein TTHERM_01046810 [Tetrahymena thermophila]	gi|118369583 (+1)	145 kDa		2	
1807	☑	☆	cytochrome P450 monooxygenase CYP5012A2 [Tetrahymena thermophila]	gi|164519855	58 kDa		2	
1808	☑	☆	hypothetical protein TTHERM_00118720 [Tetrahymena thermophila]	gi|118355016	21 kDa		2	
1809	☑	☆	hypothetical protein TTHERM_00578550 [Tetrahymena thermophila]	gi|118379380	13 kDa		2	
1810	☑	☆	hypothetical protein TTHERM_00497200 [Tetrahymena thermophila]	gi|118389690	43 kDa		2	
1811	☑	☆	3-oxo-5-alpha-steroid 4-dehydrogenase family protein [Tetrahymena thermophila]	gi|118361680	36 kDa		2	
1812	☑	☆	hypothetical protein TTHERM_00077380 [Tetrahymena thermophila]	gi|118364916	150 kDa		2	
1813	☑	☆	hypothetical protein TTHERM_01001530 [Tetrahymena thermophila]	gi|146183983	178 kDa		2	

Probability Legend

- over 95%
- 80% to 94%
- 50% to 79%
- 20% to 49%
- 0% to 19%

#	Visible?	Starred?	Bio View: Identified Proteins (1837)	Accession Number	Molecular Weight	Protein Grouping Ambiguity	01 Control	02 Sample
1814	☑	☆	hypothetical protein TTHERM_00046930 [Tetrahymena thermophila]	gi|229595689	28 kDa		2	
1815	☑	☆	hypothetical protein TTHERM_00151440 [Tetrahymena thermophila]	gi|146180901	41 kDa		2	
1816	☑	☆	CRAL/TRIO, N-terminus family protein [Tetrahymena thermophila]	gi|146173043	41 kDa		2	
1817	☑	☆	hypothetical protein TTHERM_00194690 [Tetrahymena thermophila]	gi|146169611	10 kDa		2	
1818	☑	☆	hypothetical protein TTHERM_00348520 [Tetrahymena thermophila]	gi|118379683	35 kDa		2	
1819	☑	☆	Adenylate and Guanylate cyclase catalytic domain protein [Tetrahymena thermophila]	gi|118401208	118 kDa		2	
1820	☑	☆	Transcription factor Dp-2 [Tetrahymena thermophila]	gi|118362700	89 kDa		2	
1821	☑	☆	hypothetical protein TTHERM_00835120 [Tetrahymena thermophila]	gi|118397889	53 kDa		2	
1822	☑	☆	hypothetical protein TTHERM_00285250 [Tetrahymena thermophila]	gi|118370678	257 kDa		2	
1823	☑	☆	PX domain containing protein [Tetrahymena thermophila]	gi|118352290	70 kDa		2	
1824	☑	☆	hypothetical protein TTHERM_00196140 [Tetrahymena thermophila]	gi|118368117	128 kDa		2	
1825	☑	☆	hypothetical protein TTHERM_00790950 [Tetrahymena thermophila]	gi|146183436	81 kDa		2	
1826	☑	☆	60s Acidic ribosomal protein [Tetrahymena thermophila]	gi|118354178	31 kDa		2	
1827	☑	☆	TPR Domain containing protein [Tetrahymena thermophila]	gi|146180576	132 kDa		2	
1828	☑	☆	Protein kinase domain containing protein [Tetrahymena thermophila]	gi|146181519	52 kDa		2	
1829	☑	☆	hypothetical protein TTHERM_00298270 [Tetrahymena thermophila]	gi|118382610	290 kDa		2	
1830	☑	☆	EF hand family protein [Tetrahymena thermophila]	gi|146175974	225 kDa		2	
1831	☑	☆	EF hand family protein [Tetrahymena thermophila]	gi|118367969	260 kDa		2	
1832	☑	☆	hypothetical protein TTHERM_00755860 [Tetrahymena thermophila]	gi|118398776	142 kDa	★	2	
1833	☑	☆	SPFH domain / Band 7 family protein [Tetrahymena thermophila]	gi|146184885	35 kDa		2	
1834	☑	☆	FUN14 family protein [Tetrahymena thermophila]	gi|118374371	14 kDa		2	
1835	☑	☆	Zinc carboxypeptidase family protein [Tetrahymena thermophila]	gi|118376442	81 kDa		2	
1836	☑	☆	KE2 family protein [Tetrahymena thermophila]	gi|118359002	17 kDa		2	
1837	☑	☆	hypothetical protein TTHERM_00112450 [Tetrahymena thermophila]	gi|118354758	44 kDa		2	
