# Supplementary material for: A Single Cohesin Complex Performs Mitotic and Meiotic Functions in the Protist Tetrahymena
Source: PLoS Genet. 2013 Mar 28;9(3):e1003418. doi: 10.1371/journal.pgen.1003418 (PMC3610610; doi:10.1371/journal.pgen.1003418)
Supplement: Table S2 — List of oligonucleotides. (PDF) [file pgen.1003418.s008.pdf]

Supplemental Table S2. List of oligonucleotides

Oligos used in RNAi vector construction	
PmeESPiFor	CGT TTA AAC GAG TAC TGA TGA TCC AAA GG
SmaESPiRev	GCC CGG GGG TAT TTC ACT CAA ATA AAG TGC
ApaESPiFor	CGG GCC CGA GTA CTG ATG ATC CAA AGG
XhoESPiRev	GCT CGA GGG TAT TTC ACT CAA ATA AAG TGC
PmeREC8iFW	CGT TTA AAC CTA CAG AAG AGA GAC TGA ATA TG
ApaREC8iFW	CGG GCC CCT ACA GAA GAG AGA CTG AAT ATG
SmaREC8iRV	GCC CGG GCT TTC AGA GAT GTC GCC AAC
XhoREC8iRV	GCT CGA GCT TTC AGA GAT GTC GCC AAC
SMC1iBamFW	CAG GAT CCG GCA AAA ATA GCA GAC AC
SMC1iPmeRV	CAG TTT AAA CCT TGC AAC CTT TTC TCA ATT TC
SMC1iPstFW	GAC TGC AGG GCA AAA ATA GCA GAC AC
SMC1iSmaRV	GTC CCG GGC TTG CAA CCT TTT CTC AAT TTC
SPO11iPmeFW	GTT TAA ACT TAT GGA TTG AAA CAA ACT CTG C
SPO11iApaFW	GGG CCC TTA TGG ATT GAA ACA AAC TCT GC
SPO11iSmaRV	CCC GGG ATT TGG CGA TTA AGG CAT CC
SPO11iXhoRV	CTC GAG ATT TGG CGA TTA AGG CAT CC

Oligos used in Rec8-GFP, Smc1-HA and Scc3-mCherry tagging	
Rec8-EGfpRV	AAG TTC TTC ACC CTT AGA AAC CAT GGA TCC TTT GAT AAG AAT TTG TAT TGG TGA AAA ATG
5AmRec8FW	GCT GAT GGC GAT GAA TGA ACA CTG GTG CTC CTT TCT GAA GAA C
Neo4-Rec8FW	CCC GGG GGA TCT GAA TTC GAT ATC AAG CTT CCT GTT TAA GAT AGC TAT GGC
3Am-Rec8RV	GCG AGC ACA GAA TTA ATA CGA CTC ATC GAA TTC ATG CTT ACA CC
SMC1 5'rev	CAG GAA CAT CAT AAG GAT AGG ATC CTG CTT TTA AAT TTA AAG ATA AGG TGA ATG
SMC1 5'for	CAT GTC GAC GAA GGC AGA AGA AGA TCTC
NheI-Scc3for	GCT AGC CAG ATA TGT TAA GAA CCT AGA ATC ATG GG
BamHI-Scc3rev	GGA TCC ATT CTT CTT TTA TCT TCT ATG ATT TTT TTT

	AAC AG
Neo4-Scc3for	CCC GGG GGA TCT GAA TTC GAT ATC AAG CTT GAA TTT AGT CAG TAG AAA GTA ATT TG
Scc3-3’rev	GGG CCC CTA AGT ATT CGA AAA CTC TTT TTA TGA TTG TAT TC
