# Supplementary material for: A Single Cohesin Complex Performs Mitotic and Meiotic Functions in the Protist Tetrahymena
Source: PLoS Genet. 2013 Mar 28;9(3):e1003418. doi: 10.1371/journal.pgen.1003418 (PMC3610610; doi:10.1371/journal.pgen.1003418)
Supplement: Text S1 — Search for α-kleisin homologs and Scc3 homologs. Description of the bioinformatic methods used to identify cohesin homologs in Tetrahymena. (PDF) [file pgen.1003418.s009.pdf]

**Text S1**

**Search for α-kleisin homologs**

The cleavable subunit of the cohesin complex is a member of the conserved family of α-kleisin proteins [1]. Eukaryotic kleisins of the Rad21/Rec8/Scc1 family show characteristic conserved N- and C-terminal regions with the Pfam definitions PF04825 and PF04824, respectively (pfam.sanger.ac.uk). Using the Pfam-provided Hidden Markov Model (HMM) for the C-terminal winged helix domain [2], we searched the *Tetrahymena* proteome [3]. Using HMMER3.0 [4] we found a significant match (E-value=0.004) in region aa 579-607 of the 619 amino acid-protein encoded by ORF TTHERM_00245660. Significance improved further (E=0.00034) when using an extended C-terminal alignment of PF04824 seed sequences with similarity to aa 556-617 of TTHERM_00245660p in proteome-wide profile searches.

Independent confirmation of the similarity of TTHERM_00245660 protein to the Rad21/Rec8/Scc1 family was obtained by reciprocal profile-profile searches against the Pfam database using alignments of the N- and C-terminal domains of *T. thermophila* TTHERM_00245660 protein and its homologue of the related ciliate *Ichthyophthirius multifiliis*. A HHpred search [5] performed with the N-terminal domain (corresponding to aa 30-130 in the *Tetrahymena* protein) yielded PF04825 as the top hit with E-value=0.038. A search with the C-terminal domain (aa 570-619) obtained as top hits PF04824 (E=0.0033) and the bacterial kleisin domain PF02616 (E=0.1).

Another protein, TTHERM_00219160p was found as a significant and second best hit in a HMMER3.0 profile search [4] with the extended C-terminal kleisin seed alignment versus the *T. thermophila* proteome (E=0.0013). The similarity was confirmed by profile searches of the C-terminal domain of TTHERM_00219160p orthologs from *Tetrahymena malaccensis*, *T. elliotti*, *T. borealis* and *T. thermophila*) versus the *T. thermophila* proteome, which, in addition to TTHERM_00219160p itself, also identify TTHERM_00245660p (Rec8) as the second best significant hit (E=0.00035). Similarly, a HHpred search [5] with the same TTHERM_00219160-based alignment versus Pfam (pfam.sanger.ac.uk) obtained the Rec8/Rad21 domain (PF04824) as the top hit with a borderline significance of E=0.061.

**Search for Scc3 homologs**

The domain architecture of Scc3 orthologs is dominated by well conserved helical repeats that might form elongated structural elements [6]. The set of *S. cerevisiae* Scc3 (YIL026C) orthologous proteins as defined in the inparanoid database (http://inparanoid.sbc.su.se/ - [7]) includes representatives in plants, a wide range of fungi/metazoa and amoebozoa. These Scc3 orthologs were aligned and the region of best sequence conservation corresponding to aa 134-1067 in yeast Scc3 (NP_012238.1) was used in profile-based searches against the *Tetrahymena* proteome. TTHERM_00225630p emerged as the only significant hit and the most likely SCC3 ortholog in this species (HMMsearch3: E=4.7e-06; HMMsearch2: 0.0064). HHpred search with the N-terminal 400 aa of TTHERM_00225630 (XP_001014194) confirmed the similarity (KOG2011, STAG/IRR1/SCC3 E=5.4e-05).


1. Schleiffer A, Kaitna S, Maurer-Stroh S, Glotzer M, Nasmyth K, et al. (2003) Kleisins: a superfamily of bacterial and eukaryotic SMC protein partners. Mol Cell 11: 571-575.
2. Haering CH, Schoffnegger D, Nishino T, Helmhart W, Nasmyth K, et al. (2004) Structure and stability of cohesin´s Smc1-kleisin interaction. Mol Cell 15: 951-964.
3. Eisen JA, Coyne RS, Wu M, Wu D, Thiagarajan M, et al. (2006) Macronuclear genome sequence of the ciliate *Tetrahymena thermophila,* a model eukaryote. PLoS Biol 4: 1621-1642.
4. Finn RD, Clements J, Eddy SR (2011) HMMER web server: interactive sequence similarity searching. Nucl Acids Res 39 Web Server Issue: W29-W37.
5. Söding J, Biegert A, Lupas AN (2005) The HHpred interactive server for protein homology detection and structure prediction. Nucl Acids Res 33: W244-W248.
6. Haering CH, Löwe J, Hochwagen A, Nasmyth K (2002) Molecular architecture of SMC proteins and the yeast cohesin complex. Mol Cell 9: 773-788.
7. Ostlund G, Schmitt T, Forslund K, Köstler T, Messina DN, et al. (2010) InParanoid 7: new algorithms and tools for eukaryotic orthology analysis. Nucl Acids Res 38 Database Issue: D196-D203.
